# Modeling PFAS Sorption in Soils Using Machine Learning

**DOI:** 10.1021/acs.est.4c13284

**Published:** 2025-04-11

**Authors:** Joel Fabregat-Palau, Amirhossein Ershadi, Michael Finkel, Anna Rigol, Miquel Vidal, Peter Grathwohl

**Affiliations:** † Department of Geosciences, University of Tübingen, Schnarrenbergstraße 94-96, Tübingen 72076, Germany; ‡ Department of Chemical Engineering and Analytical Chemistry, University of Barcelona, Martí i Franquès 1-11, Barcelona 08028, Spain; § Institut de Recerca de l’Aigua (IdRA), Universitat de Barcelona, Martí i Franquès 1-11, Barcelona 08028, Spain

**Keywords:** speciation, stacking model, data set, *K*_d_ sensitivity, spatial map

## Abstract

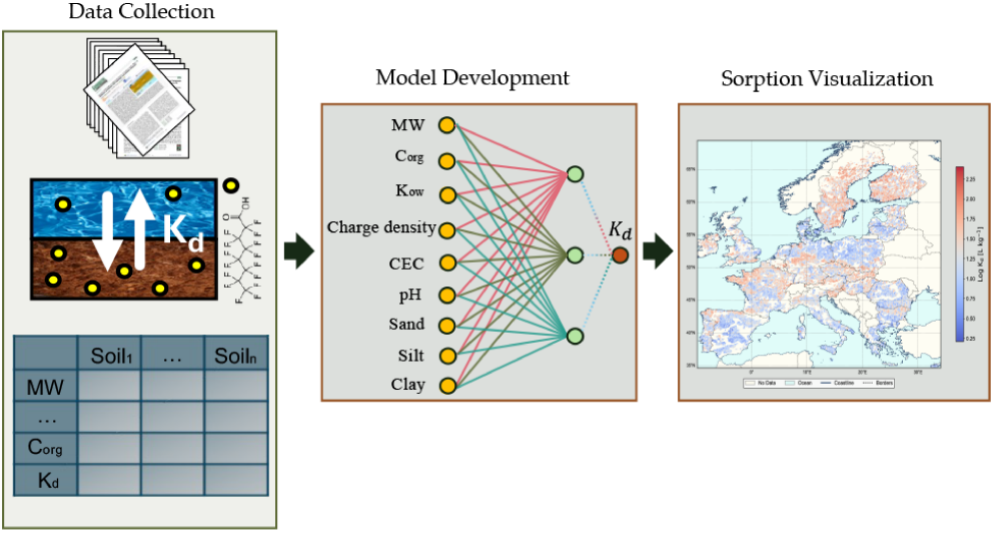

In this study, we introduce PFASorptionML, a novel machine learning (ML) tool developed to predict solid–liquid distribution coefficients (*K*_d_) for per- and polyfluoroalkyl substances (PFAS) in soils. Leveraging a data set of 1,274 *K*_d_ entries for PFAS in soils and sediments, including compounds such as trifluoroacetate, cationic, and zwitterionic PFAS, and neutral fluorotelomer alcohols, the model incorporates PFAS-specific properties such as molecular weight, hydrophobicity, and p*K*_a_, alongside soil characteristics like pH, texture, organic carbon content, and cation exchange capacity. Sensitivity analysis reveals that molecular weight, hydrophobicity, and organic carbon content are the most significant factors influencing sorption behavior, while charge density and mineral soil fraction have comparatively minor effects. The model demonstrates high predictive performance, with RPD values exceeding 3.16 across validation data sets, outperforming existing tools in accuracy and scope. Notably, PFAS chain length and functional group variability significantly influence *K*_d_, with longer chain lengths and higher hydrophobicity positively correlating with *K*_d_. By integrating location-specific soil repository data, the model enables the generation of spatial *K*_d_ maps for selected PFAS species. These capabilities are implemented in the online platform PFASorptionML, providing researchers and practitioners with a valuable resource for conducting environmental risk assessments of PFAS contamination in soils.

## Abstract 2

A comprehensive dataset of PFAS solid−liquid distribution coefficients (*K*_d_) in soils was used to develop a machine learning model, offering *K*_d_ (PFAS) predictions that are useful for environmental management.

## Introduction

Per- and polyfluoroalkyl substances (PFAS) are anthropogenic compounds characterized by high environmental persistence due to their strong C–F bonds.^1^ Currently, PFAS are defined as substances that contain at least one fully fluorinated methyl or methylene carbon atom (without any H/Cl/Br/I atom attached to it).^2^ These compounds are broadly classified as perfluorinated, where all hydrogen atoms are replaced by fluorine, or polyfluorinated, where only some hydrogens are replaced. PFAS can also be categorized into short- and long-chained compounds based on the number of fluorinated carbons, with short-chain perfluorosulfonic acids (PFSA) and perfluorocarboxylic acids (PFCA) having less than six and seven fluorinated carbons, respectively.^3^ PFAS represent a diverse chemical class with extensive industrial and consumer applications, including aqueous fire-fighting foams (AFFF), electronics, construction materials, and coatings for paper products.^4,5^ Anionic-type PFAS such as PFCA and PFSA have been widely studied for their occurrence, sorption, and toxicity,^6−8^ but neutral, cationic, and/or zwitterionic PFAS may constitute the majority of PFAS at certain AFFF-contaminated sites.^9,10^ The sorption behavior of some of these PFAS has been overlooked, albeit current regulatory restrictions consider PFAS as a whole class.^11^

PFAS contamination is widespread, with concentrations in soils ranging from a few pg g^–1^ in remote areas to hundreds of μg g^–1^ at impacted sites.^7,12^ Soils act as both filters and sources for PFAS contamination in groundwater.^13^ Sorption parameters, such as the solid–liquid distribution coefficient (*K*_d_), are essential for modeling contaminant transport under saturated and unsaturated conditions. In unsaturated soils, however, the air–water distribution coefficient (*K*_aw_) should also be considered, as it may lead to the retention of contaminants at the air–water interfaces.^14,15^ Therefore, understanding PFAS sorption in environmental matrices is essential for mitigating associated environmental concerns by informing risk assessment, remediation strategies, and regulatory decision-making. *K*_d_ values may be estimated from organic-carbon-normalized sorption coefficients (*K*_OC_) or by prediction models that account for certain soil and PFAS properties.^16−21^ These tools have limitations, such as their narrow PFAS range and reliance on oversimplified assumptions that overlook complex PFAS–soil interactions. Moreover, studies assessing the predictive performance of existing models, as well as a systematic analysis of PFAS features affecting sorption (e.g., the effect of PFAS chain length and functional groups), are lacking.

The compilation of *K*_d_ data sets enriched with both PFAS and soil characteristics by examining literature studies is critical for modeling purposes.^21−23^ Machine learning (ML) has emerged as a powerful method for modeling complex, nonlinear relationships within large, multidimensional data sets to uncover patterns and correlations that conventional models might overlook.^23−26^ Recently, Xie and coworkers developed an ML model on a literature-based *K*_d_ (PFAS) data set consisting of 2,328 entries for 26 different anionic PFAS.^21^ Acceptance data criteria considered *K*_d_ values for the same PFAS/soil pairs at different pH conditions, especially those originating from a single study.^27^ However, the speciation of ionizable PFAS with p*K*_a_ values within environmentally relevant pH ranges (e.g., perfluorosulfonamides (FOSA), p*K*_a_ ≈ 6)^28^ was not considered.

In this study, we systematically compiled a comprehensive literature-based *K*_d_ PFAS data set, incorporating 1,274 entries for 51 different PFAS, including anionic, neutral, cationic, and zwitterionic species. Using ML algorithms, we developed a novel model that integrates both PFAS- (i.e., molecular weight (MW), hydrophobicity, p*K*_a_) and soil-specific (i.e., pH, texture, cation exchange capacity (CEC), organic carbon content (*C*_org_)) descriptors. The model demonstrated superior predictive performance compared to other existing tools and, when combined with location-specific soil data (e.g., EU LUCAS topsoil repository), enabled the generation of spatial *K_d_* maps. The data set and model are available through the online platform PFASorptionML (https://hydrogeochem.geo.uni-tuebingen.de/pfas).

## Materials and Methods

### PFAS Considered in This Work

**Figure 1:**
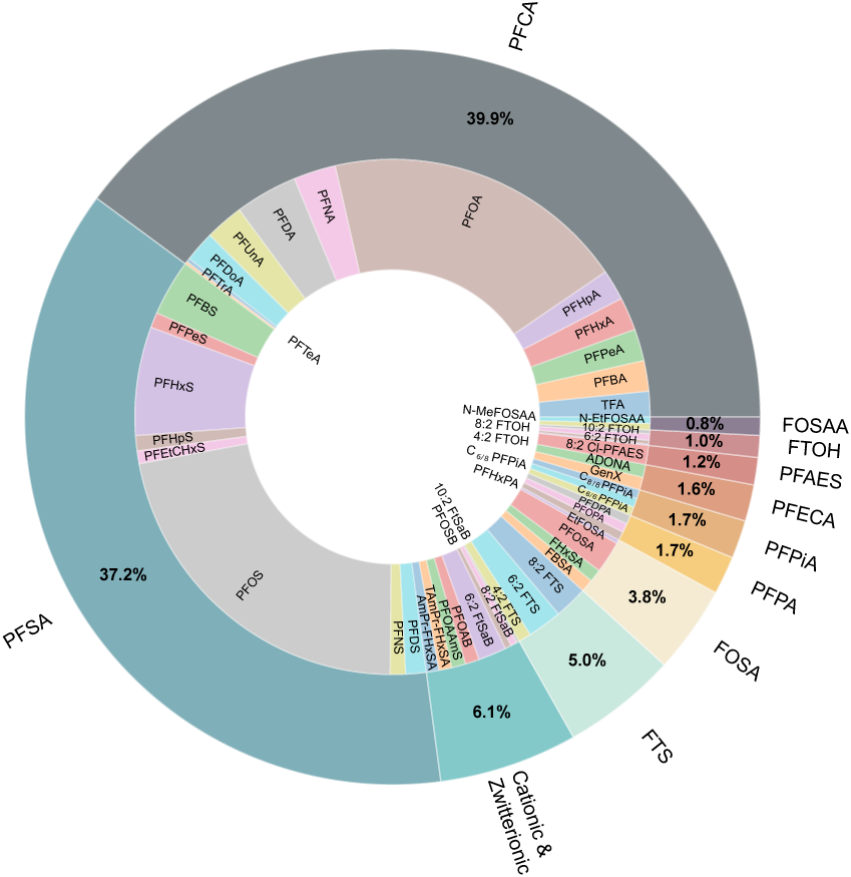
Relative distribution of PFAS subfamilies and respective compounds in the *K*_d_ (PFAS) data set, including perfluorocarboxylic acids (PFCA), perfluorosulfonamides (FOSA), perfluorosulfonic acids (PFSA), fluorotelomer sulfonates (FTS), perfluorophosphonic acids (PFPA), fluorotelomer alcohols (FTOH), perfluoroalkyl ether carboxylic acids (PFECA), perfluorooctane sulfonamidoacetic acids (FOSAA), chlorinated polyfluoroalkyl ether sulfonates (PFAES), perfluorophosphinic acids (PFPiA), and cationic and zwitterionic PFAS.

Sorption data for a total of 51 PFAS were included in the data set. These data comprise C_2_–C_14_ PFCA (i.e., TFA, PFBA, PFPeA, PFHxA, PFHpA, PFOA, PFNA, PFDA, PFUnA, PFDoA, PFTeA and PFTrA) and C_4_–C_10_ PFSA, (i.e., PFBS, PFPeS, PFHxS, PFHpS, PFOS, PFNS, and PFDS), including a cyclic (i.e., PFEtCHxS) species. PFCA and PFSA have been widely detected in soils and water at varying concentrations,^7,29^ and they are known for their resistance to biodegradation. Particular attention was given to TFA due to its high volatility and low sorption affinity to soils, resulting in elevated levels in the atmosphere (≤7 ng m^–3^), water (≤3 μg L^–1^), and soils (≤2 ng g^–1^).^30,31^ The data set also includes C_6_–C_10_ perfluorophosphonic acids (PFPA, i.e., PFHxPA, PFOPA, PFDPA) and C_12_–C_16_ perfluorophosphinic acids (C_*x*/*y*_ PFPiA, i.e., C_6/6_ PFPiA, C_6/8_ PFPiA and C_8/8_ PFPiA). Both PFPA and PFPiA have been detected in water bodies and human serum samples, with concentrations ranging from 0.1 to 3.7 ng L^–1^ and 4 to 38 ng L^–1^, respectively.^29,32^ Additionally, the data set includes C_4_–C_8_ FOSA (i.e., FBSA, FHxSA, PFOSA, and Et-FOSA), as well as *N*-methyl and *N*-ethyl perfluorooctane sulfonamidoacetic acids (FOSAA, i.e., *N*-MeFOSAA and *N*-EtFOSAA) species. These PFAS have been detected in water bodies at concentrations ranging 2.5–5.8 ng L^–1^.^33^ Emerging PFAS classes were also included, such as perfluoroalkyl ether carboxylic acids (PFECA, i.e., GenX and ADONA) and chlorinated polyfluoroalkyl ether sulfonate (PFAES, i.e., 8:2 Cl-PFAES). GenX and 6:2 Cl-PFAES have been found in water bodies at high concentrations (<5 and ≤112 μg L^–1^) close to fluorochemical facilities and wastewater treatment plant effluents, respectively.^29,34^ Furthermore, data for C_4_–C_10_*n*:2 fluorotelomer alcohols (FTOH, i.e., 4:2, 6:2, 8:2 and 10:2 FTOH) and C_4_–C_8_*n*:2 fluorotelomer sulfonates (FTS, i.e., 4:2, 6:2 and 8:2 FTS) were included. FTOH are known degradation products of side-chain fluorinated polymers and have been found in air samples at ≤0.3 μg m^–3^,^35,36^ whereas FTS are compounds present in AFFF-formulations and have been found in impacted sites at ≈4 ng g^–1^.^7^ Additionally, we included data for C_6_–C_10_ cationic and zwitterionic PFAS (i.e., 6:2, 8:2 and 10:2 FtSaB, 6:2 FtSaAm, PFOSB, PFOAAmS, PFOAB, AmPr-FHxSA and TAmPr-FHxSA), which are predominant species in AFFF-formulations and have been found in both AFFF-impacted soil and groundwaters.^9,10^ The relative distribution of PFAS subfamilies and compounds in the data set is illustrated in Figure 1, while detailed information on their chemical structures, CAS numbers, and physicochemical properties is provided in Section S1.

Physicochemical properties of the PFAS were obtained from available data or estimated using EPISuite and the PubChem repository. Specifically, EPISuite (KOWWIN method versions 1.68 and 2.00) was used to estimate water solubility (*S*) and organic carbon-normalized sorption coefficients (*K*_OC_), respectively. Octanol–water partition coefficients (*K*_OW_) were obtained from PubChem’s XLogP3 (version 3.0, released 2021.10.14). Sorption of charged compounds may be highly dependent on the amount and type of charged species.^22^ To include PFAS speciation in the model, acidity dissociation constants (i.e., p*K*_a_) were compiled. Although commonly studied PFCA and PFSA compounds have low p*K*_a_ values (p*K*_a_ ≈ 1), resulting in their presence as anionic species under most environmental conditions, some other PFAS compounds such as FOSA and some betaines exhibit p*K*_a_ values more aligned with environmental conditions (i.e., p*K*_a_ ≈ 6).^28,37^ The pH-speciation diagrams for representative PFAS are detailed in Section S2.

### Criteria for Data Compilation

To be considered in the data set, sorption data for PFAS must originate from a batch test following official guidelines meant to study sorption under saturated conditions at room temperature (i.e., 20–25 °C)^38^ with slight variations (e.g., selection of contact solution) permitted. Data originated from other sources (e.g., *K*_d_ distribution originating from ratios of site-measured concentrations in both solids and liquid matrices) were not considered.^6^ The data set covers information on the data source and PFAS properties (i.e., MW, empirical formula, *S*, *K*_OW_, and p*K*_a_). We accepted sorption data reported on both soil and sediments, assuming analogous sorption behavior among these matrices in batch tests, but we excluded sorption data in pure organic substances (e.g., humic acids) or mineral (e.g., goethite, kaolinite) phases. The main physicochemical properties of the solids (i.e., pH, Fe and Al contents, CEC, *C*_org_, and soil texture information (i.e., sand, silt, and clay contents)), as well as experimental details of the batch experiment (i.e., nature of the contact solution, solid-to-liquid ratio, initial PFAS concentration range) were additionally compiled for better understanding of sorption behavior.

*K*_d_ (PFAS) values were compiled as a representative sorption parameter. Straightforward reported *K*_d_ values, either originating from a low single-concentration spike sorption experiment^17,27^ or derived from the lower concentration range of the sorption isotherm,^39,40^ were considered. If several *K*_d_ (PFAS) values were reported from nonlinear isotherm data at varying concentrations,^41^ the *K*_d_ value reported at the lowest concentration was preferred, as a better representative of PFAS environmental concentrations.^7,29^*K*_d_ (PFAS) values were similarly derived when needed from reported *K*_OC_ data.^42,43^ Contrarily to other studies that accepted *K*_d_ (PFAS) values for the same PFAS/sorbent pair at different pH and/or ionic strength conditions,^21^ we adopted here stricter selection criteria. Only *K*_d_ values measured under conditions closely matching the original soil pH or at the lowest ionic strength were included.^27,44^ While this approach reduced the number of *K*_d_ entries in the data set, it ensured a better representation of the original soil properties in the model.

In some cases, isotherm fitting parameters were reported, but no *K*_d_ (PFAS) values. Although some authors have estimated *K*_d_ values from isotherm parameters at a certain arbitrary concentration within the low isotherm range,^22,45^ in this study, we derived *K*_d_ (PFAS) data from isotherm fitted parameters (mainly resulting from Freundlich and, to a lesser extent, Langmuir fits) at the same PFAS concentration in water relative to the reported PFAS solubility (i.e., 10%), in agreement with other previous work.^46^ Further details on the derivation of *K*_d_ (PFAS) values from literature studies are provided in Section S3. Overall, 94 and 184 *K*_d_ (PFAS) entries originated from *K*_OC_ and isotherm fitted data (7% and 14% of the total entries, respectively). During the data set construction, expert judgment was applied to identify individual entries that exhibited potential outlier behavior, defined here as unexpectedly high or low *K*_d_ (PFAS) values, particularly identified by examining individual *K*_d_ vs *f*_OC_ relationships (see Supplementary File). Subsequently, we assessed entire PFAS compound classes for potential outlier trends, particularly by examining log *K*_OC_ vs log *K*_OW_ relationships. Section S4 provides a summary of the number of entries and publications contributing data for each PFAS, while the complete data set is available in Supplementary File. Based on our assessment, 47 out of the 1,274 *K*_d_ (PFAS) entries (4% of the total) were classified as outliers, leaving a final data set of 1,227 entries representative of 47 PFAS spanning 451 soil and sediment types across 47 studies.

### Model Development

#### Feature Engineering

In developing the ML model, we carefully selected and engineered both PFAS- and soil-specific features. The soil-specific properties used in the model include soil pH [-], CEC [cmol_+_ kg^–1^], *C*_org_ [%], and textural (i.e., sand [%], silt [%], and clay [%] contents) information. These features were chosen based on their availability and established influence on PFAS sorption. Other soil properties, such as iron (Fe) and aluminum (Al) contents, were excluded due to limited data availability (i.e., only 45% of the entries included available data), along with contradictory findings in the literature regarding their relevance to PFAS sorption.^27,47^ Regarding soil pH, sorption of PFAS has been shown to decrease when increasing the pH of the batch contact solution for a given soil type.^27^ CEC is mainly the result of the soil organic matter content and, to some extent, of clay minerals.^48^ While sorption of neutral and anionic PFAS in soils is known to be highly affected by *C*_org_,^17^ sorption of cationic and zwitterionic organic compounds has been better related to CEC.^22,41^ Soil textural information was also included in the model, as clay minerals may impact PFAS sorption in scenarios of low *C*_org_.^17,49^

Since the *K*_d_ (PFAS) data set we compiled from literature contained gaps in soil physicochemical properties (i.e., pH, CEC, and soil texture; see Supplementary File), we constructed an additional data set of selected soil properties (i.e., pH, CEC, *C*_org_, and soil texture; see Supplementary File) using the SoilGrids repository.^50^ This soil data set consists of 2,039 entries distributed worldwide and was used to develop a K-nearest neighbor (KNN) imputer model,^51^ which allowed prediction of the soil property data gaps in the *K*_d_ (PFAS) data set. This approach to addressing data gaps is an upgrade compared to other studies, where *K*_d_ modeling was restricted to the availability of specific soil properties,^22,45^ and has the potential to be implemented in further studies. Information on the KNN imputer model construction and implementation is provided in Section S5.

PFAS-specific properties included in the model are MW [g mol^–1^], log *K*_OW_ [-], and, for the first time, the molar net charge over MW ratio (i.e., charge density [C g^–1^]). MW was selected as a general descriptor of PFAS properties. Whereas *K*_OW_ was chosen as a good representative of the hydrophobic interaction between the PFAS alkyl chain and soil organic matter,^17^ the charge density is indicative of the electrostatic interaction between the PFAS functionalities and the charged soil surfaces. The net sign of this novel feature allowed inclusion of PFAS speciation in the model and differentiation of PFAS among cationic (charge density >0), neutral or zwitterionic (charge density ≈0), and anionic (charge density <0) species at the particular pH condition, as well as differentiation of the charge density among PFAS of the same subfamily (e.g., anionic PFBA and PFOA species). The abundance of each PFAS species was calculated according to the Henderson–Hasselbalch equation, ultimately providing information on the net charge of PFAS molecules in the system.^22^ For PFAS compounds containing a single ionizable group, the relative abundance of each species (*A*_*r*_(PFAS)_*i*_) at a given soil pH was calculated using
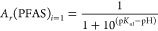
1
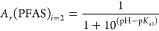
2where *i* denotes the different PFAS species at a certain pH condition (see Section S2). For those PFAS that contained two ionizable groups, *A*_*r*_(PFAS)_*i*_ was calculated as
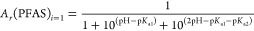
3
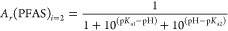
4
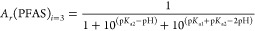
5

Whether these fractions were cationic, zwitterionic, anionic, or neutral was dependent on each PFAS pH-speciation diagram (see Section S2). The molar net charge of each PFAS species (*z*(PFAS)_*i*_ [-]) was also dependent on the pH-speciation diagram (see Section S2). Due to PFAS speciation under specific pH conditions, MW was recalculated to reflect the relative abundances of different species (e.g., accounting for protonated and deprotonated forms), thus defining an effective molecular weight at the specific pH condition. The charge density [C g^–1^] was then determined as
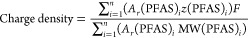
6where *F* is the Faraday constant [C mol^–1^].

The ranges of PFAS and soil properties used in the training set ultimately set the applicability boundaries of the model. A histogram of the distribution of entry values for each feature included in the model is shown in Figure S2. The imputed soil property values follow the same distribution pattern as the original (i.e., raw) *K*_d_ (PFAS) data set. MW of the selected PFAS ranged from 114 to770 g mol^–1^, representative of PFAS with a fluorinated load (%F) ranging from 43 to 72% w/w. The charge density ranged from −854 to 188 C g^–1^. Soil pH ranged from 2.8 to 9.0, and *C*_org_ ranged from 0.03 to 54%, and was skewed to those soils with low *C*_org_ (i.e., < 2%). Individual examinations of the relative entry distribution across different *C*_org_ ranges were assessed for some PFAS with *N* > 10 entries (see Figure S3) and indicated a lack of sorption data in organic soils. Soil textural fractions ranged from 0 to 100% for sand and silt, and 0 to 69% for clay, indicating that the soils cover a wide range of textural characteristics (see Figure S4). CEC ranged from 0.1 to 140 cmol_+_ kg^–1^, and log *K*_d_ (PFAS) ranged from −1.4 to 3.95, thus spanning over 5 orders of magnitude.

#### *K*_d_ (PFAS) Model Construction and Validation

To ensure that all features contributed equally to the learning process, each feature was normalized using min-max scaling, thus enabling a balanced contribution in the model^52^ (see details in Section S6). Later, the data was randomly split using the holdout method (see details in Section S6), with 80% allocated for model training and 20% allocated for testing. This split ensures that the testing set remains independent from the training set, allowing for a better assessment of the model’s performance.^53^

The predictive model for *K*_d_ (PFAS) was then constructed using a stacking ensemble approach,^54^ a two-layer framework that combines the strengths of multiple ML algorithms. The resulting model was termed as PFAS Sorption Stacking Model (PSSM). In the first layer, four base models-Ridge,^55^ Random Forest,^56^ Extremely Randomized Trees,^57^ and Gradient Boosting^58^ were trained on the input features **X**, denoted as (*h*_*m*_(**X**)|*m* = 1, 2, ..., *M* = 4). These models generated a set of predictions *ŷ*_*m*_ = *h*_*m*_(**X**), which served as intermediate outputs for the subsequent layer. These predictions from the base models were then combined into a new data set **Z**, where **Z** = {*ŷ*_1_, *ŷ*_2_, ..., *ŷ*_*M*_}, to be used as input for the second layer (i.e., meta-model). In this study, the meta-model was a multi-layer perceptron.^59,60^ The meta-model learned to optimally combine the predictions from the base models, leveraging their collective strengths and generating the final output (*ŷ*_final_) as shown in eq 7:
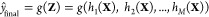
7

The predictive performance of the PSSM was evaluated using the normalized root mean squared error (NRMSE), the k-fold cross-validated normalized root-mean-square error (CV-NRMSE), and the ratio of performance to deviation (RPD) (see Section S7 for additional information on these metrics).

### Sensitivity Analysis for *K*_d_ (PFAS) Model

Understanding the sensitivity of a model to different input features provides insights into the robustness and reliability of the model and helps to explain model outputs. Two primary methods for sensitivity analysis in ML are SHapley Additive exPlanations (SHAP)^61^ and Partial Dependence (PD).^62^ SHAP is a popular method based on cooperative game theory that provides model-agnostic, locally accurate explanations for individual predictions.^61^ SHAP values quantify each feature’s contribution by estimating the average marginal impact across all possible feature combinations.^63^ This approach ensures consistent, fair evaluations of each feature’s influence across different combinations.^64^ The SHAP value (ϕ*_i_*) for feature *i,* is computed as:
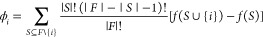
8where *F* represents the full set of features, *S* is a subset of features excluding feature *i*, |*S*| denotes the number of features in subset *S*, *f*(*S*) represents the model prediction when only features in subset *S* are used, *f*(*S* ∪ {*i*}) is the model prediction when feature *i* is added to subset *S*, and the term  is a weighting factor that considers all possible permutations of feature subsets, ensuring that each feature’s contribution is fairly evaluated across different combinations.

PD, in contrast, provides a global view of the relationship between a specific feature and the predicted outcome by averaging out the influence of all other features.^58^ PD analysis helps reveal the general trend of a feature’s impact on predictions across the data set. The PD of a feature *x*_*j*_ on the predicted outcome *ŷ* (i.e., log *K*_*d*_ values) is calculated as:
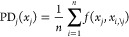
9where *f*(*x*_*j*_, *x*_*i*,\*j*_) is the model’s prediction when feature *x*_*j*_ is set to a specific value, while all other features *x*_*i*,\*j*_ remain as in each observation *i*, *n* is the number of observations, *x*_*j*_ is the feature for which PD is computed, and *x*_*i*,\ *j*_ represents the remaining features in each observation, excluding *x*_*j*_.

## Results and Discussion

### Effect of Chain Length in PFAS Sorption

For each entry in the data set, *K*_d_ was converted to *K*_OC_ to account for variability caused by the differing *C*_org_ values of the soil.^17^ Then, log *K*_OC_ was used for assessing the role of PFAS chain length and functional group in sorption. Statistical tests were conducted to group the different log *K*_OC_ populations (see details in Section S8). The mean log *K*_OC_ data for PFCA ranged from 1.18 to 5.25 and increased with the number of fluorinated carbons, in line with other observations (Figure S6).^17,27^ The *K*_OC_ populations for PFCA with fewer than six fluorinated carbons were statistically equal and generally increased for each additional CF_2_ moiety. Similar outcomes were observed for PFSA (Figure S7), FOSA (Figure S8), FTOH (Figure S9), FTS (Figure S10), and PFPA (Figure S11), but this trend was not evident for the cationic and zwitterionic PFAS (Figure S12) and PFPiA (Figure S13) subfamilies. The minor dependence of *K*_OC_ on the chain lengths for short PFAS is evident in our data set, but it is not entirely clear how potential experimental artifacts may contribute to this (e.g., reliable determination of very small *K*_d_ values at large liquid-to-solid ratios and thus potential overestimation). Overall, the log *K*_OC_ for specific PFAS subfamilies generally increased with increasing chain length, especially for PFAS with six or more fluorinated carbons, likely due to the more effective hydrophobic interaction with soil organic matter.^17,27^

### Effect of Functional Group in PFAS Sorption

Log *K*_OC_ populations were also assessed to elucidate the effect of the PFAS functional group for those compounds with a certain number of fluorinated carbons (i.e., 4, 6, 8, and 10; see Figures S14–S17). Log *K*_OC_ populations were generally similar regardless of the functional group for those PFAS with less than seven fluorinated carbons (Figures S14 and S15), but a significant effect of the functional group was noted for PFAS with more than six fluorinated carbons (Figures S16 and S17). The sorption affinity trend followed: PFPA ≲ PFCA ≲ PFSA ≈ FTS < FOSA ≲ FTOH ≲ cationic and zwitterionic PFAS. The lower sorption observed for PFPA could be attributed to their double negatively charged phosphonate group, in contrast to the single negative charge of the carboxylate group of PFCA, leading to a more effective electrostatic repulsion with the negatively charged surfaces, thereby decreasing its sorption.^65^ Higher sorption observed for PFSA compared to that of PFCA agreed with previous observations^17^ and may result from the greater hydrophobicity provided by the sulfonate moiety. In this line, log *K*_OC_ values for telomer sulfonate were higher than those for sulfonate groups, likely due to the additional hydrophobicity provided by the −CH_2_–CH_2_– moiety. Log *K*_OC_ values for sulfonamide and, especially, telomer alcohol functional groups were significantly higher than others, likely due to their generally uncharged nature, which prevents electrostatic repulsions with negatively charged particles, thereby increasing its sorption.^65^ Log *K*_OC_ values for the cationic and zwitterionic PFAS were the highest, likely a result of the overestimation of *K*_OC_ due to attraction between the positively charged PFAS species and the negatively charged clay surfaces, thereby increasing sorption.^22^ Similarly, log *K*_OC_ data for PFAES was the highest observed, likely as a result of the overestimation of *K*_OC_ due to data originating from mineral soils (i.e., *C*_org_ < 2.7%).^17,27^

### Identification of Outliers and Anomalous Trends

**Figure 2:**
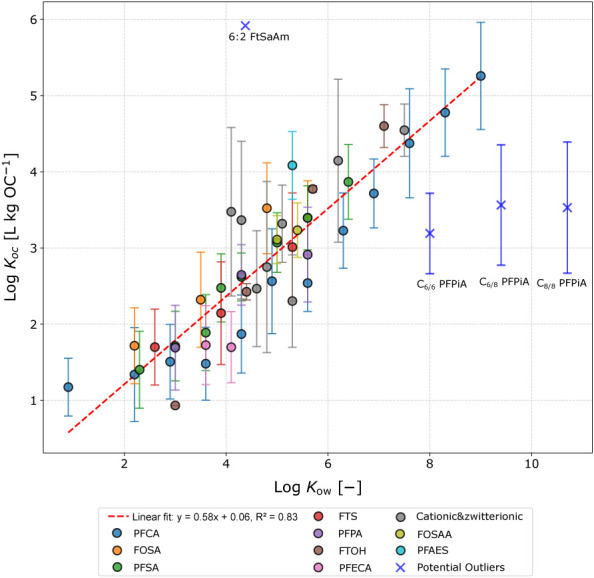
Trend between log *K*_OC_ and log *K*_OW_ for different PFAS species, including perfluorocarboxylic acids (PFCA), perfluorosulfonamides (FOSA), perfluorosulfonic acids (PFSA), fluorotelomer sulfonates (FTS), perfluorophosphonic acids (PFPA), fluorotelomer alcohols (FTOH), perfluoroalkyl ether carboxylic acids (PFECA), perfluorooctane sulfonamidoacetic acids (FOSAA), chlorinated polyfluoroalkyl ether sulfonate (PFAES), perfluorophosphinic acids (PFPiA), and cationic and zwitterionic PFAS. Identified PFAS outliers are marked as “X”. The dashed line shows a regression of the overall data, excluding outliers. Whiskers represent the standard deviation of the original data.

PFAS hydrophobicity (i.e., *K*_OW_) increases with the number of fluorinated carbons of the PFAS. According to preliminary data explorations, a positive correlation between *K*_OC_ and *K*_OW_ was anticipated due to hydrophobic interaction playing an important role in sorption.^17^ Average log *K*_OC_ data was therefore screened against log *K*_OW_, obtaining a positive linear relationship regardless of the subfamily PFAS type (see Figure 2). Nonetheless, four PFAS species (i.e., 6:2 FtSaAm, C_6/6_ PFPiA, C_6/8_ PFPiA, and C_8/8_ PFPiA) exhibited a noted discrepant trend. To the best of our knowledge, sorption of C_*x*/*y*_ PFPiA has only been evaluated in a single study,^66^ which concluded that sorption of these species did not increase with organic content, contrary to observations for other PFCA and PFSA.^17^ The obtention of new experimental data supported by additional spectroscopic or computational evidence will be beneficial to confirm if sorption of C_*x*/*y*_ PFPiA is driven by other unknown sorption mechanisms. On the other hand, sorption of 6:2 FtSaAm originated from a single soil sample with low organic content (i.e., 0.1%),^41^ suggesting the overestimation of *K*_OC_ due to sorption originating mainly from mineral components.^22^ The *K*_*d*_ data for these four PFAS were considered as outliers for further model assessments. The relationship between log *K*_OC_ and log *K*_OW_ for PFAS (log *K*_OC_ = 0.58 log *K*_OW_ + 0.06; *n* = 47; *r*^2^ = 0.83; Figure 2) differs from linear correlations observed for other organic pollutants, such as polycyclic aromatic hydrocarbons (PAH, log *K*_OC_ = 0.97 log *K*_OW_ + 0.12; *n* = 106; *r*^2^ = 0.93) and several nonpolar hydrophobic organic compounds (HOC, log *K*_OC_ = 1.10 log *K*_OW_ + 0.99; *n* = 418; *r*^2^ = 0.95),^67^ likely due to the charged nature of most PFAS considered in this work.

#### Model Performance and Sensitivity Analysis

**Figure 3:**
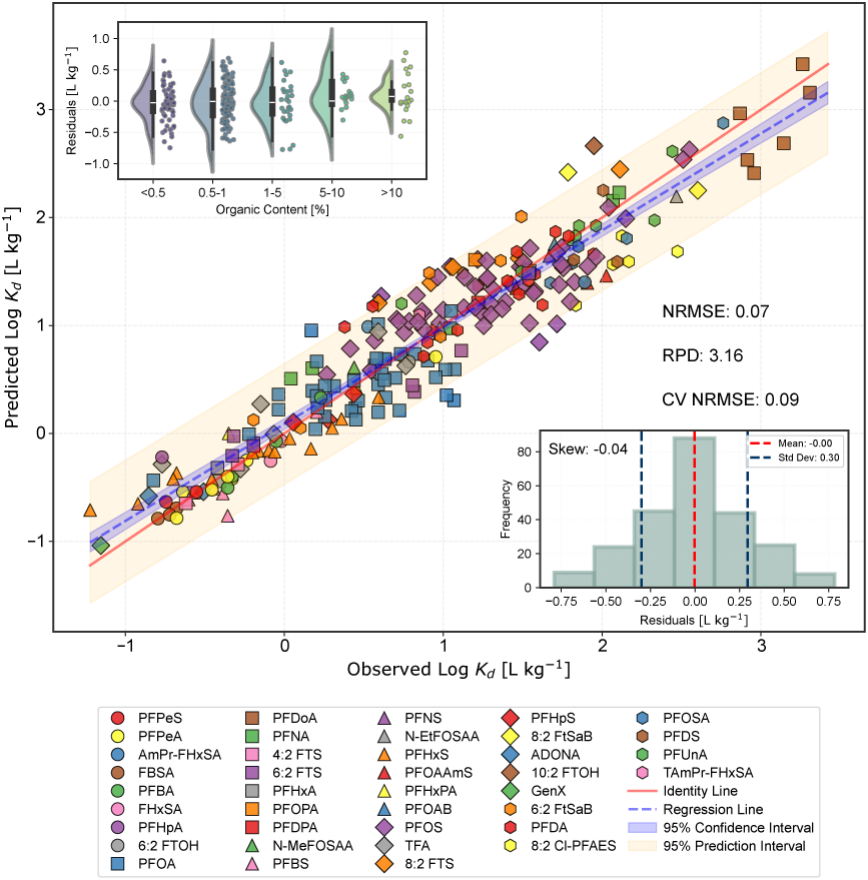
Scatter plot comparing predicted and measured log *K*_d_ values for various PFAS species. The lower-right subplot illustrates the distribution of residuals, with a mean of approximately zero log *K*_d_ units, a standard deviation of 0.30, and a skewness of −0.04 log *K*_d_ units. The upper-left subplot represents the residual distribution stratified by different levels of soil organic content.

For all the PFAS species considered, the predicted log *K*_d_ values are in excellent agreement with the observed ones (Figure 3), with a slope close to one (i.e., 0.90) and a *y*-intercept close to zero (i.e., 0.09). NRMSE and RPD values of 0.07 and 3.16, respectively, and a 10 *k*-fold CV-NRMSE value of 0.09 indicate an excellent prediction ability of the model. Residual evaluations show a residual mean of approximately null log *K*_d_ units, evidencing no general under- or overpredictions, and a standard deviation of 0.30. The skewness of the residual distribution was −0.04 log *K*_d_ units, with no evident bias along soil *C*_org_ (Figure 3). Additional modeling attempts, based on the same test set, excluding the charge density feature, resulted in worse predictions of *K*_d_ (PFAS), with an NRMSE of 0.08 and an RPD of 2.59.

The PSSM shows better prediction performance compared to other currently available *K*_d_ (PFAS) prediction tools, such as those derived from models only considering both organic and mineral sorption sites^16,17^ (see Section S9). In addition to the good performance for commonly predicted PFCA and PFSA, the model successfully predicts *K*_d_ values for TFA in two soils of contrasting characteristics, as well as *K*_d_ values for different cationic and zwitterionic PFAS such as PFOAB, PFOAAmS, AmPr-FHxSA, TAmPr-FHxSA, and 6:2 FtSaB (Figure 3).

Although sorption of ionizable compounds can vary greatly depending on pH due to different speciation,^22^ the accurate prediction for cationic, zwitterionic and FOSA species for soils with differing pH confirms the need for incorporating PFAS speciation into the model. Good predictions were also observed for PFPA, FOSA, FOSAA, PFECA, PFAES, FTS, and FTOH groups. Overall, the successful validation and the high metric qualities of the model highlight its potential use in risk assessment studies aiming to evaluate PFAS mobility in contaminated sites. Nonetheless, while the model demonstrates strong predictive capability for sorption under saturated conditions and effectively incorporates PFAS speciation, its applicability is limited to compounds and soils within the predefined property ranges, as ML struggles to accurately extrapolate predictions to other scenarios. Additionally, our model can be extended by incorporating other physical factors that influence PFAS transport, such as the air–water interface under unsaturated conditions, which is ultimately governed by soil moisture and, consequently, subject to seasonal variations. Regarding seasonal variations, the model so far does not account for temperature-induced changes in *K*_d_ values for PFAS (according to previous studies, *K*_d_ may increase by up to 60% if temperatures rise from 15 to 35 °C).^68^

**Figure 4:**
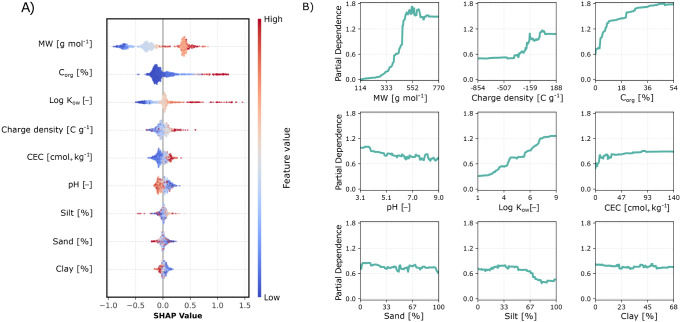
(A) SHAP summary plot illustrating the influence of individual features on *K*_d_ predictions. Features are ranked from top to bottom by their importance on model’s output, with each dot representing a single sample. The color gradient from blue (low) to red (high) indicates the feature value, while the horizontal position reflects the model’s output change, where negative SHAP values correspond to lower output values and positive SHAP values indicate higher output values. (B) Partial dependence (PD) showing the marginal effect of each input feature on *K*_d_, averaged across all samples.

The sensitivity analysis (Figure 4) reveals that MW is the most sensitive feature, particularly distinguishing PFAS species. Higher MW values correlate with increased log *K*_d_, as shown by both SHAP values (Figure 4A) and PD analysis (Figure 4B). Unlike the findings of Xie and coworkers,^21^ who identified pH as the second most influential feature, our analysis shows *C*_org_ as the predominant soil property influencing sorption. This result aligns with the strong affinity of PFAS for organic matter, indicating that the key influence of pH in Xie’s model may originate from including *K*_d_ values for the same PFAS/soil combinations at different pH levels. In our model, *C*_org_ contributes positively to log *K*_d_, suggesting organic carbon content as a key soil driver of PFAS sorption, as confirmed by both SHAP and PD analyses. Log *K*_OW_ emerged as the third most important feature, with both SHAP and PD indicating that high log *K*_OW_ values correlate with high log *K*_d_ (also supported by findings in Figure 2). The influence of both *C*_org_ and *K*_OW_ supports the hypothesis that hydrophobic interactions are a central mechanism for PFAS sorption.^17^ Additionally, charge density was identified as the fourth most influential feature, suggesting minimal impact of electrostatic interaction on PFAS sorption. CEC ranked fifth in influence, with sorption increasing alongside higher CEC values, potentially due to indirect associations with higher *C*_org_ contents and increased availability of exchangeable sorption sites, especially suitable to sorb cationic and zwitterionic species.^41^ Conversely, increasing pH generally decreased *K*_d_ (PFAS), likely due to promoting a higher abundance of negatively charged PFAS species and an increasing number of negative charges in clay (pH_ZPC_ ≈ 3) and *C*_org_ (pH_ZPC_ ≈ 8) fractions,^22^ thus increasing electrostatic repulsions with these domains. Finally, soil texture had a limited effect on the model’s predictions, suggesting that grain sizes play a minor role in PFAS sorption compared to *C*_org_. However, including these variables may still enhance prediction accuracy in low *C*_org_ scenarios.^17^

## Environmental Implications

**Figure 5:**
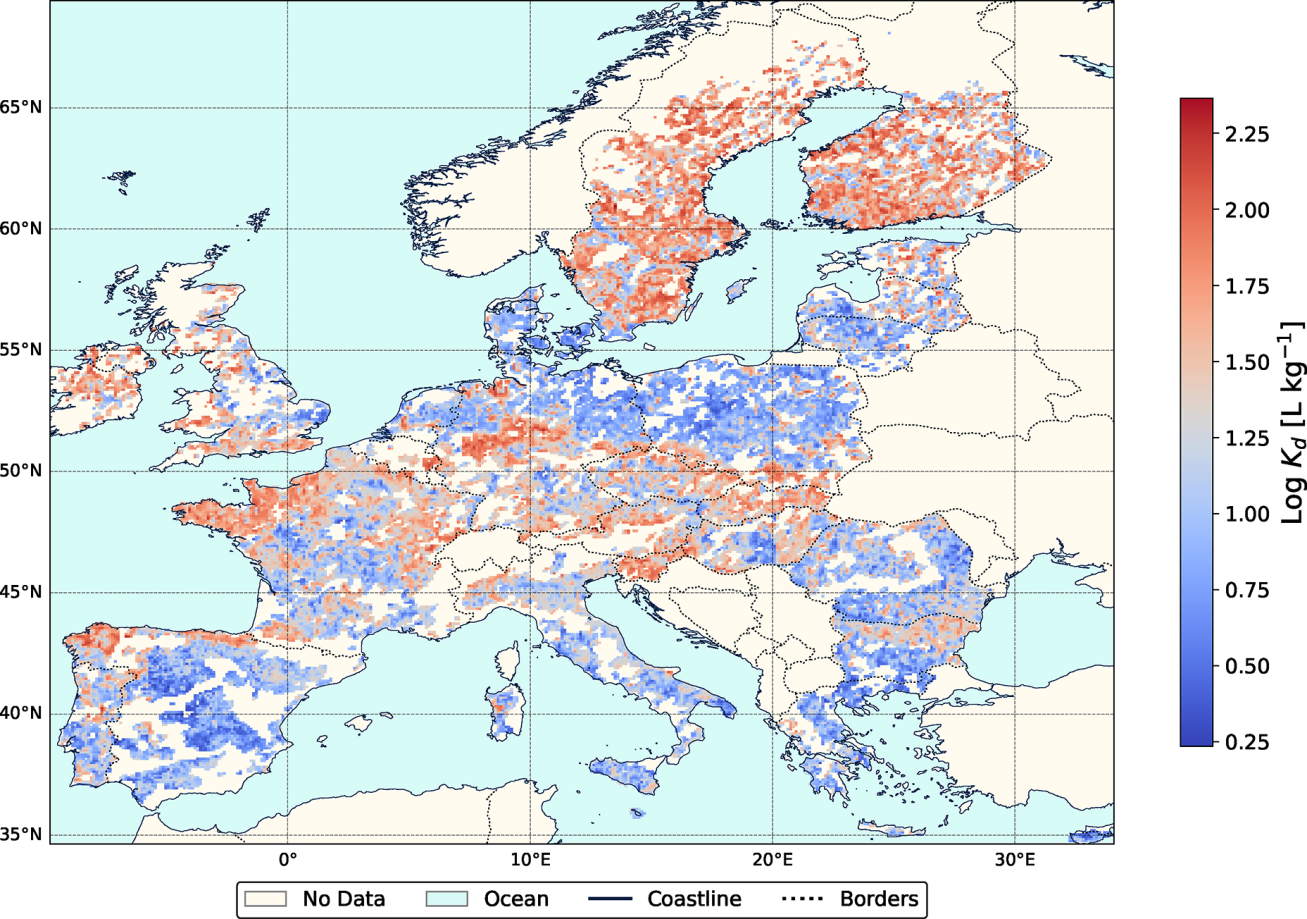
Predicted log *K*_d_ values for PFOSB across Europe using the stacking model based on soil properties from the LUCAS 2009 repository. Regions without available soil data are indicated as ″No Data″.

The developed model (i.e., PSSM) offers broad applicability to predict *K*_d_ for 47 PFAS by leveraging both PFAS-specific and soil-dependent input parameters. With this flexibility, the model can be applied to spatial soil property data repositories at any scale and resolution to generate *K*_d_ (PFAS) prediction maps. To give an example, we applied the PSSM to the soil LUCAS 2009 repository data for European soils (European Soil Data Centre: ESDAC). Figure 5 shows the *K*_d_ map for PFOSB, while additional information and *K*_d_ maps for other PFAS species (i.e., TFA, PFOA, PFOS) are available in Section S10. Overall, the model allows the identification of geographical zones with potentially low *K*_d_ values (i.e., locations where PFAS may have higher mobility), thus facilitating its transport to the groundwater table. Therefore, the combination of our model output with other PFAS topsoil concentration data and groundwater information can be used in global PFAS transport studies across the saturated zone.^14,24^ However, these large-scale assessments may overlook local heterogeneities in soil properties, potentially introducing additional uncertainty in *K*_d_ predictions unless high-resolution geospatial data is available.

To overcome these soil spatial heterogeneity limitations, we provide an online platform (PFASorptionML) for end-users to predict site-specific *K*_d_ (PFAS) values. The platform is free to use at https://hydrogeochem.geo.uni-tuebingen.de/pfas. Input parameters required are the PFAS to be assessed (i.e., CAS number, full name or abbreviation of the PFAS) and specific soil properties (i.e., pH, *C*_org_, CEC, soil texture). In case users lack some of these soil properties, our KNN imputer model developed in Section S5 facilitates their prediction. Using this information, PFAS-specific pH-speciation plots are generated and *K*_d_ predictions are produced. Furthermore, the platform allows the generation of *K*_d_ maps at any scale (e.g., EU) for all 47 PFAS considered in this study. Further assessments of the model toward other PFAS that currently lack sorption data are of interest and subject to further work. Upgrades of the platform are planned, including the generation of additional geographical *K*_d_ maps (e.g., Worldwide) and the consideration of additional transport parameters such as specific air–water interfaces and *K*_aw_ in unsaturated conditions. In addition to topsoil properties, subsoils can be included to allow more realistic transport modeling across the vadose zone into groundwater. The findings of this study, considering the limitations of the PSSM model, can be integrated into risk assessment evaluations to assess the mobility of PFAS in soils and aquifers.

## Supporting Information Available

Physicochemical properties and pH speciation diagram for PFAS; derivation of *K*_d_ and *K*_OC_ data from literature studies; KNN imputer model; data preprocessing; model metrics and performance comparison; statistical analyses of *K*_OC_ distributions; and geospatial maps (PDF)
*K*_d_ PFAS data set, including PFAS compounds, soil and batch properties, and reported sorption parameters (XLSX)
