# Supplementary material for: Modeling PFAS Sorption in Soils Using Machine Learning
Source: Environ Sci Technol. 2025 Apr 11;59(15):7678–87. doi: 10.1021/acs.est.4c13284 (PMC12020356; doi:10.1021/acs.est.4c13284)
Supplement: Supplementary file 1 — es4c13284_si_001.pdf [file es4c13284_si_001.pdf]

Supporting information

for

Modeling PFAS Sorption in Soils Using Machine Learning

Joel Fabregat-Palau [Θ, a, *], Amirhossein Ershadi [Θ, a, *], Michael Finkel [a], Miquel Vidal [b], Anna Rigol [b, c],

Peter Grathwohl [a]

[a] *Department of Geosciences, University of Tübingen, Schnarrenbergstraße 94-96, 72076 Tübingen, Germany*

[b] *Department of Chemical Engineering and Analytical Chemistry, University of Barcelona, Martí i Franquès 1-11, Barcelona 08028, Spain*

[c] *Institut de Recerca de l'Aigua (IdRA), Universitat de Barcelona, Martí i Franquès 1-11, Barcelona 08028, Spain*

[Θ] These authors share first authorship

* Corresponding author email: joel.fabregat-palau@uni-tuebingen.de; amirhossein.ershadi@uni-tuebingen.de

Number of Pages: 60

Number of Figures: 23

Number of Tables: 3

Table of contents

Section S1: Chemical structures and physicochemical properties of the PFAS — Page S3
Section S2: pH-speciation diagram for some representative PFAS species — Page S11
Section S3: Additional information on the derivation of K_d data from literature studies — Page S27
Section S4: Number of entries and references used to derive log K_{OC} values — Page S30
Section S5: Construction of a ML soil property imputer model based on KNN — Page S31
Section S6: Additional information on data preprocessing — Page S36
Section S7: Additional information on the model metrics to assess predictive performance — Page S37
Section S8: Statistical tests to assess log K_{OC} distributions across different PFAS subfamilies — Page S39
Section S9: Comparison of model performance with other available tools — Page S47
Section S10: Additional geospatial K_d (PFAS) maps — Page S51
Section S11: References — Page S57

List of Figures

Figure S1. Spatial distribution of the soil samples for developing KNN model — Page S31
Figure S2. Soil and PFAS properties ranges considered in the model — Page S33
Figure S3. Entry distribution over organic content ranges for some selected PFAS species — Page S34
Figure S4. Visualization of soil pH, C_{ORG} and texture information for training and validation sets — Page S35
Figure S5. Additional information on the statistical test flowchart used to assess log K_{OC} populations — Page S39
Figure S6. Distribution of K_{OC} data for different PFCA — Page S41
Figure S7. Distribution of K_{OC} data for different PFSA — Page S41
Figure S8. Distribution of K_{OC} data for different FOSA — Page S42
Figure S9. Distribution of K_{OC} data for different FTOH — Page S42
Figure S10. Distribution of K_{OC} data for different FTS — Page S43
Figure S11. Distribution of K_{OC} data for different PFPA — Page S43
Figure S12. Distribution of K_{OC} data for different Cationic and Zwitterionic PFAS — Page S44
Figure S13. Distribution of K_{OC} data for different PFPiA — Page S44
Figure S14. Distribution of K_{OC} data for different PFAS species containing four fluorinated carbons — Page S45
Figure S15. Distribution of K_{OC} data for different PFAS species containing six fluorinated carbons — Page S45
Figure S16. Distribution of K_{OC} data for different PFAS species containing eight fluorinated carbons — Page S46
Figure S17. Distribution of K_{OC} data for different PFAS species containing ten fluorinated carbons — Page S46
Figure S18. Visualization of the comparative prediction accuracy of available K_d prediction tools — Page S50
Figure S19. Spatial distribution of selected soil properties within the LUCAS soil repository — Page S52
Figure S20. Geospatial K_d (TFA) information — Page S53
Figure S21. Geospatial K_d (PFOA) information — Page S54
Figure S22. Geospatial K_d (PFOS) information — Page S55
Figure S23. Geospatial K_d (PFOSB) information — Page S56

List of Tables

Table S1. Summary of PFAS compounds, their properties, and chemical structures — Page S3
Table S2. Number of entries and references used to derive log K_{OC} values — Page S30
Table S3. Comparison of performance metrics across different prediction tools — Page S49

S1: Chemical structures and physicochemical properties of the PFAS

Table S1. Summary of PFAS compounds, their properties, and chemical structures.

**PFAS acronym**	**CAS number**	**Subfamily**	**Chemical structure**	**Log K_{OW}**<br>**Log S**<br>**Log K_{OC}**	**pK_{a1}**<br>**pK_{a2}**
6:2 FtSaB	34455-29-3	Zwitterionic PFAS (Betaine)	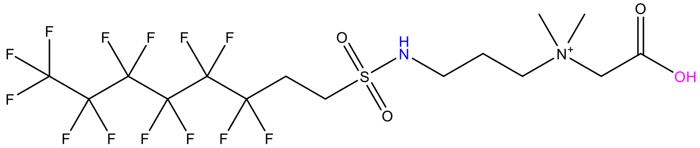	Log K_{OW} = 4.80<br>Log S = 3.30<br>Log K_{OC} = 1.81	pK_{a1} = 2.3 [1]<br>pK_{a2} = 11.1 [1]
8:2 FtSaB	34455-21-5	Zwitterionic PFAS (Betaine)	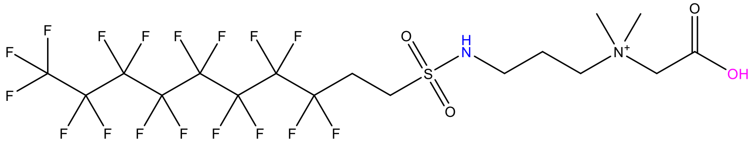	Log K_{OW} = 6.20<br>Log S = 1.50<br>Log K_{OC} = 2.54	pK_{a1} = 2.3 *<br>pK_{a2} = 11.1 *
10:2 FtSaB	34455-35-1	Zwitterionic PFAS (Betaine)	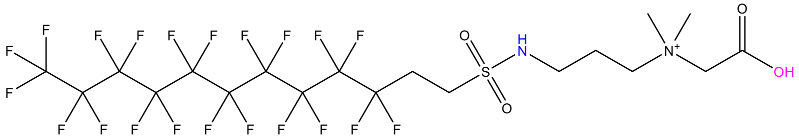	Log K_{OW} = 7.50<br>Log S = -0.31<br>Log K_{OC} = 3.30	pK_{a1} = 2.3 *<br>pK_{a2} = 11.1 *
6:2 FtSaAm	1383438-86-5	Cationic PFAS	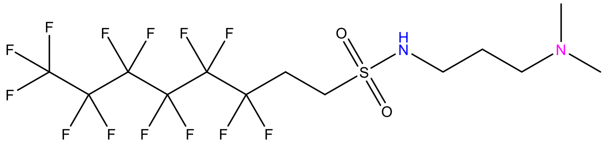	Log K_{OW} = 4.60<br>Log S = 3.12<br>Log K_{OC} = 2.71	pK_{a1} = 9.3 [2]<br>pK_{a2} = 10.7 [2]
PFOSB	75046-16-1	Zwitterionic PFAS (Betaine)	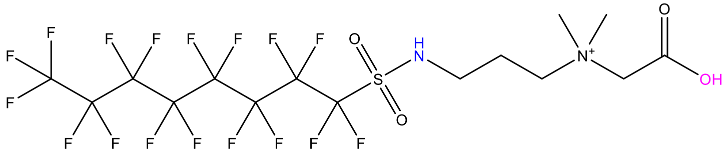	Log K_{OW} = 4.60<br>Log S = 1.26<br>Log K_{OC} = 2.83	pK_{a1} = 2.3 [1]<br>pK_{a2} = 6.8 [1]
PFOAAmS	45305-66-6	Cationic PFAS	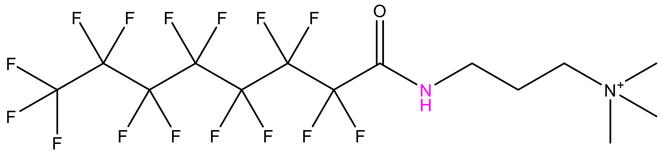	Log K_{OW} = 5.10<br>Log S = 2.45<br>Log K_{OC} = 2.96	pK_{a1} = 7.7 [1]<br>pK_{a2} = N.A.
PFOAB	90179-39-8	Zwitterionic PFAS (Betaine)	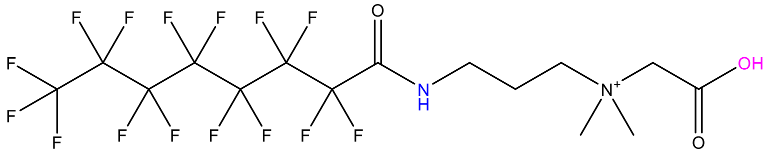	Log K_{OW} = 5.30<br>Log S = 2.80<br>Log K_{OC} = 1.78	pK_{a1} = 2.3 [1]<br>pK_{a2} = 7.8 [1]

PFAS acronym	CAS number	Subfamily	Chemical structure	Log K_{OW} Log S Log K_{OC}	pKa_1 pKa_2
AmPr-FHxSA	50598-28-2	Cationic PFAS	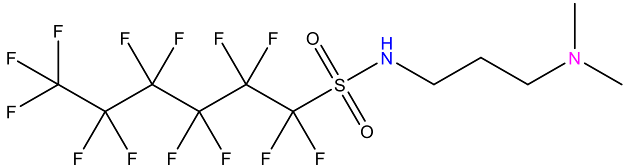	Log K_{OW} = 4.30 Log S = 2.80 Log K_{OC} = 3.83	pK_{a1} = 9.2 *[,1] pK_{a2} = 6.8 [1]
TAmPr-FHxSA	38850-51-0	Cationic PFAS	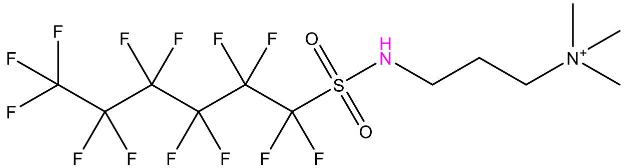	Log K_{OW} = 4.10 Log S = 2.31 Log K_{OC} = 3.27	pK_{a1} = 6.8 *[,1] pK_{a2} = N.A.
4:2 FTOH	2043-47-2	n:2 Telomer Alcohol (FTOH)	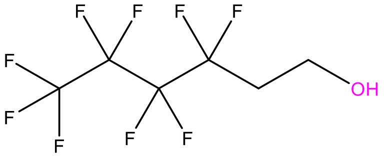	Log K_{OW} = 3.00 Log S = 4.99 Log K_{OC} = 2.34	pK_{a1} = 14.2 * pK_{a2} = N.A.
6:2 FTOH	647-42-7	n:2 Telomer Alcohol (FTOH)	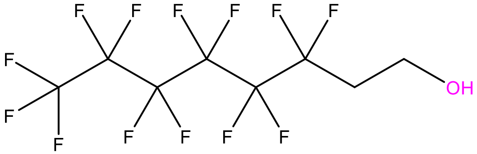	Log K_{OW} = 4.40 Log S = 3.34 Log K_{OC} = 3.02	pK_{a1} = 14.2 * pK_{a2} = N.A.
8:2 FTOH	678-39-7	n:2 Telomer Alcohol (FTOH)	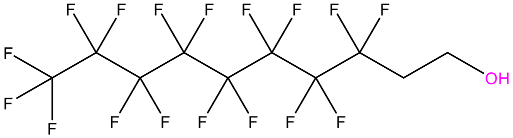	Log K_{OW} = 5.70 Log S = 1.83 Log K_{OC} = 3.60	pK_{a1} = 14.2 [3] pK_{a2} = N.A.
10:2 FTOH	865-86-1	n:2 Telomer Alcohol (FTOH)	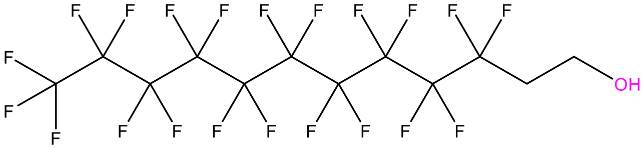	Log K_{OW} = 7.10 Log S = -0.10 Log K_{OC} = 4.43	pK_{a1} = 14.2 * pK_{a2} = N.A.
TFA	76-05-1	Carboxyl Acid (PFCA)	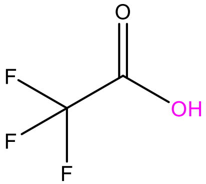	Log K_{OW} = 0.90 Log S = 7.99 Log K_{OC} = 0.43	pK_{a1} = - 0.2 * pK_{a2} = N.A.

PFAS acronym	CAS number	Subfamily	Chemical structure	Log K_{OW}<br>Log S<br>Log K_{OC}	pKa_1<br>pKa_2
PFBA	375-22-4	Carboxyl Acid (PFCA)	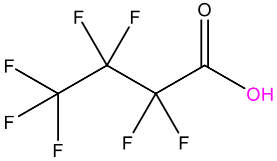	Log K_{OW} = 2.20<br>Log S = 6.14<br>Log K_{OC} = 1.34	$pK_{a1} = -0.2$ *<br>pK_{a2} = N.A.
PFPeA	2706-90-3	Carboxyl Acid (PFCA)	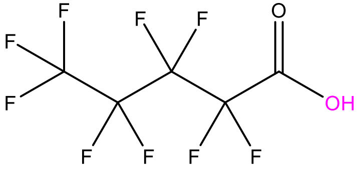	Log K_{OW} = 2.90<br>Log S = 5.29<br>Log K_{OC} = 1.71	$pK_{a1} = -0.2$ [4]<br>pK_{a2} = N.A.
PFHxA	307-24-4	Carboxyl Acid (PFCA)	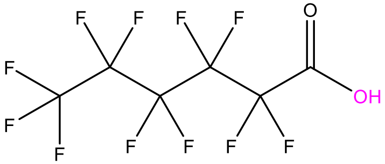	Log K_{OW} = 3.60<br>Log S = 4.43<br>Log K_{OC} = 2.08	$pK_{a1} = -0.2$ [4]<br>pK_{a2} = N.A.
PFHpA	375-85-9	Carboxyl Acid (PFCA)	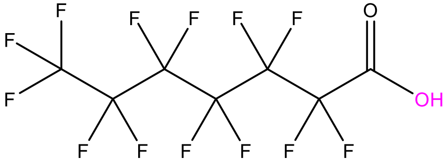	Log K_{OW} = 4.30<br>Log S = 3.56<br>Log K_{OC} = 2.45	$pK_{a1} = -0.2$ [4]<br>pK_{a2} = N.A.
PFOA	335-67-1	Carboxyl Acid (PFCA)	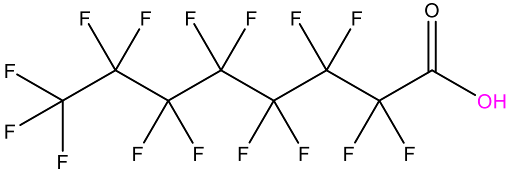	Log K_{OW} = 4.90<br>Log S = 2.68<br>Log K_{OC} = 2.82	$pK_{a1} = -0.2$ [4]<br>pK_{a2} = N.A.
PFNA	375-95-1	Carboxyl Acid (PFCA)	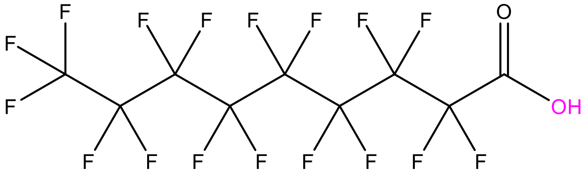	Log K_{OW} = 5.60<br>Log S = 1.80<br>Log K_{OC} = 3.19	$pK_{a1} = -0.2$ [4]<br>pK_{a2} = N.A.

PFAS acronym	CAS number	Subfamily	Chemical structure	Log K_{OW}<br>Log S<br>Log K_{OC}	pKa_1<br>pKa_2
PFDA	335-76-2	Carboxyl Acid (PFCA)	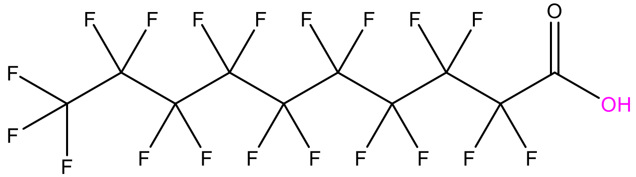	Log K_{OW} = 6.30<br>Log S = 0.90<br>Log K_{OC} = 3.56	pK_{a1} = - 0.2 [4]<br>pK_{a2} = N.A.
PFUnA	2058-94-8	Carboxyl Acid (PFCA)	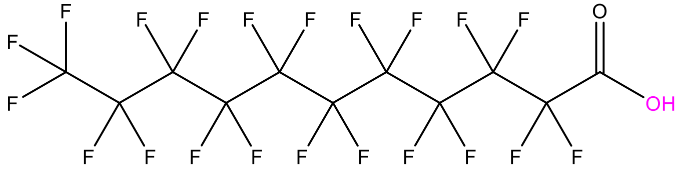	Log K_{OW} = 6.90<br>Log S = 0.01<br>Log K_{OC} = 3.93	pK_{a1} = - 0.2 [4]<br>pK_{a2} = N.A.
PFDoA	307-55-1	Carboxyl Acid (PFCA)	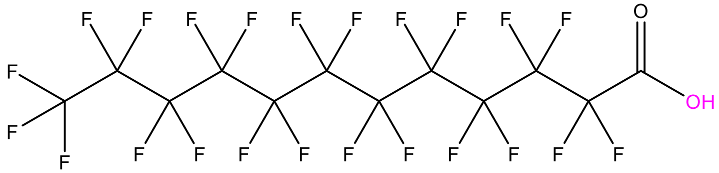	Log K_{OW} = 7.60<br>Log S = -0.89<br>Log K_{OC} = 4.30	pK_{a1} = - 0.2 [4]<br>pK_{a2} = N.A.
PFTrA	72629-94-8	Carboxyl Acid (PFCA)	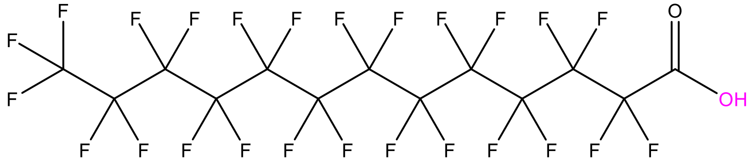	Log K_{OW} = 8.30<br>Log S = -1.79<br>Log K_{OC} = 4.67	pK_{a1} = - 0.2 [4]<br>pK_{a2} = N.A.
PFTeA	376-06-7	Carboxyl Acid (PFCA)	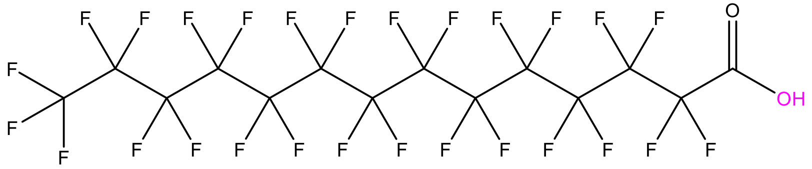	Log K_{OW} = 9.00<br>Log S = -2.69<br>Log K_{OC} = 5.04	pK_{a1} = - 0.2 [4]<br>pK_{a2} = N.A.
GenX	13252-13-6	Ether Carboxyl Acid (PFECA)	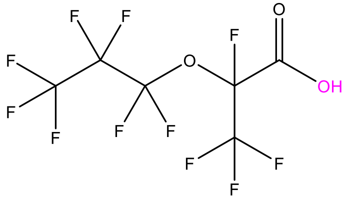	Log K_{OW} = 3.60<br>Log S = 4.43<br>Log K_{OC} = 1.92	pK_{a1} = - 0.1 [5]<br>pK_{a2} = N.A.

**PFAS acronym**	**CAS number**	**Subfamily**	**Chemical structure**	**Log K_{OW}**<br>**Log S**<br>**Log K_{OC}**	**pKa_1**<br>**pKa_2**
ADONA	919005-14-4	Ether Carboxyl Acid (PFECA)	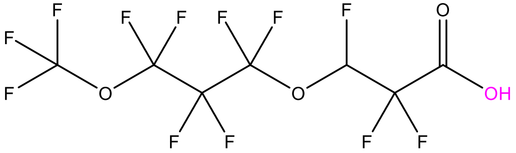	Log K_{OW} = 4.10<br>Log S = 3.97<br>Log K_{OC} = 1.94	pK_{a1} = 1.2 [6]<br>pK_{a2} = N.A.
PFBS	375-73-5	Sulfonic Acid (PFSA)	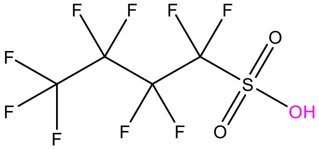	Log K_{OW} = 2.30<br>Log S = 5.54<br>Log K_{OC} = 1.93	pK_{a1} = 0.1 [4]<br>pK_{a2} = N.A.
PFPeS	2706-91-4	Sulfonic Acid (PFSA)	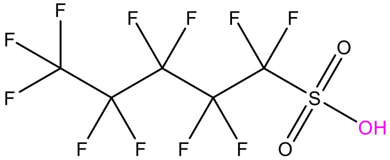	Log K_{OW} = 3.00<br>Log S = 4.67<br>Log K_{OC} = 2.30	pK_{a1} = 0.1 *<br>pK_{a2} = N.A.
PFHxS	355-46-4	Sulfonic Acid (PFSA)	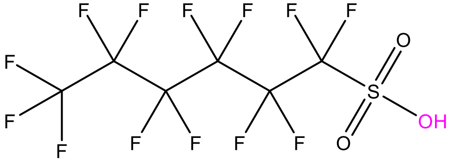	Log K_{OW} = 3.60<br>Log S = 3.79<br>Log K_{OC} = 2.67	pK_{a1} = 0.1 [4]<br>pK_{a2} = N.A.
PFHpS	375-92-8	Sulfonic Acid (PFSA)	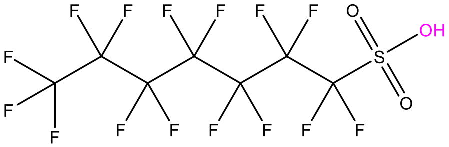	Log K_{OW} = 4.30<br>Log S = 2.91<br>Log K_{OC} = 3.04	pK_{a1} = 0.1 *<br>pK_{a2} = N.A.
PFOS	1763-23-1	Sulfonic Acid (PFSA)	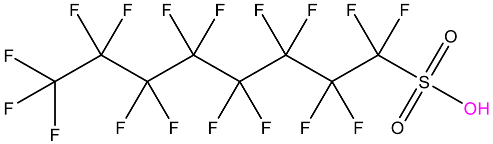	Log K_{OW} = 5.00<br>Log S = 2.91<br>Log K_{OC} = 3.41	pK_{a1} = 0.1 [4]<br>pK_{a2} = N.A.
PFNS	68259-12-1	Sulfonic Acid (PFSA)	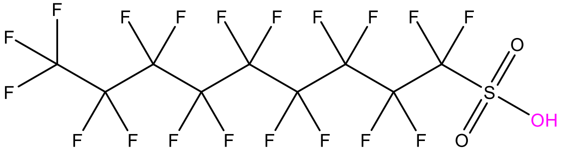	Log K_{OW} = 5.60<br>Log S = 1.12<br>Log K_{OC} = 3.78	pK_{a1} = 0.1 *<br>pK_{a2} = N.A.

PFAS acronym	CAS number	Subfamily	Chemical structure	Log K_{OW}<br>Log S<br>Log K_{OC}	pK_{a1}<br>pK_{a2}
PFDS	335-77-3	Sulfonic Acid (PFSA)	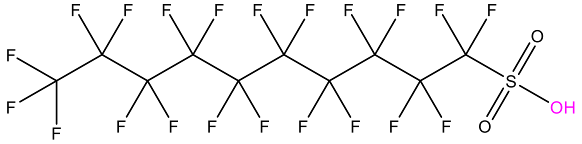	Log K_{OW} = 6.40<br>Log S = 0.22<br>Log K_{OC} = 4.15	pK_{a1} = 0.1 [4]<br>pK_{a2} = N.A.
PFEtCHxS	646-83-3	Cyclic Sulfonic Acid (PFSA)	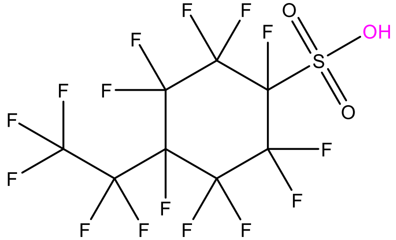	Log K_{OW} = 3.90<br>Log S = 2.32<br>Log K_{OC} = 3.37	pK_{a1} = 0.1 [*,5]<br>pK_{a2} = N.A.
8:2 Cl-PFAES	756426-58-1	Chlorinated Ether Sulfonic Acid (PFAES)	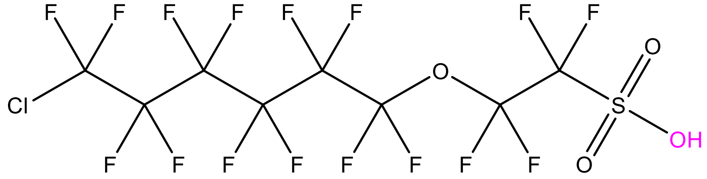	Log K_{OW} = 5.30<br>Log S = 1.17<br>Log K_{OC} = 3.73	pK_{a1} = 0.1 [5]<br>pK_{a2} = N.A.
4:2 FTS	757124-72-4	Telomer Sulfonic Acid (FTS)	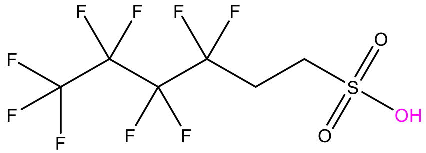	Log K_{OW} = 2.60<br>Log S = 5.79<br>Log K_{OC} = 1.66	pK_{a1} = 0.4 [4]<br>pK_{a2} = N.A.
6:2 FTS	27619-97-2	Telomer Sulfonic Acid (FTS)	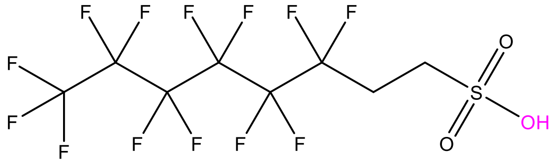	Log K_{OW} = 3.90<br>Log S = 4.04<br>Log K_{OC} = 2.40	pK_{a1} = 0.4 [4]<br>pK_{a2} = N.A.
8:2 FTS	39108-34-4	Telomer Sulfonic Acid (FTS)	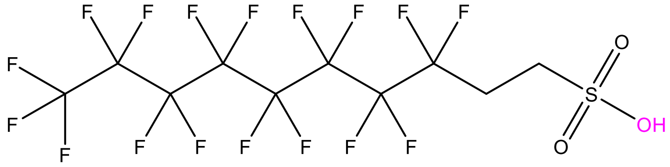	Log K_{OW} = 5.30<br>Log S = 2.26<br>Log K_{OC} = 3.14	pK_{a1} = 0.4 [4]<br>pK_{a2} = N.A.

PFAS acronym	CAS number	Subfamily	Chemical structure	Log K_{OW}<br>Log S<br>Log K_{OC}	pKa_1<br>pKa_2
FBSA	30334-69-1	Sulfonamide (FOSA)	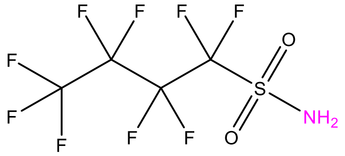	Log K_{OW} = 2.20<br>Log S = 4.43<br>Log K_{OC} = 2.82	pK_{a1} = 6.2 *<br>pK_{a2} = N.A.
FHxSA	41997-13-1	Sulfonamide (FOSA)	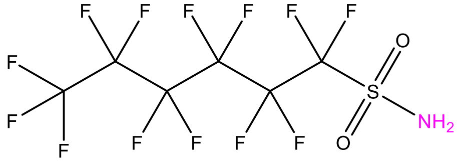	Log K_{OW} = 3.50<br>Log S = 2.68<br>Log K_{OC} = 3.55	pK_{a1} = 6.2 *<br>pK_{a2} = N.A.
PFOSA	754-91-6	Sulfonamide (FOSA)	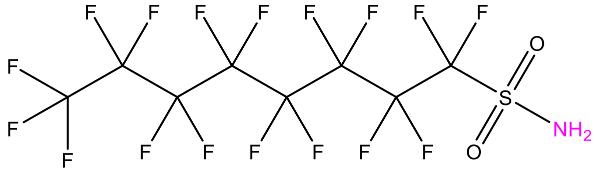	Log K_{OW} = 4.80<br>Log S = 0.90<br>Log K_{OC} = 4.30	pK_{a1} = 6.2 [7]<br>pK_{a2} = N.A.
EtFOSA	4151-50-2	Sulfonamide (FOSA)	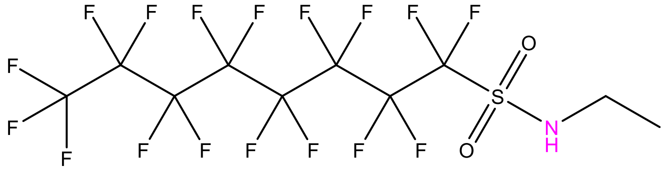	Log K_{OW} = 5.60<br>Log S = -0.10<br>Log K_{OC} = 4.80	pK_{a1} = 9.0 [7]<br>pK_{a2} = N.A.
MeFOSAA	2355-31-9	Sulfonamido Acetic acid (FOSAA)	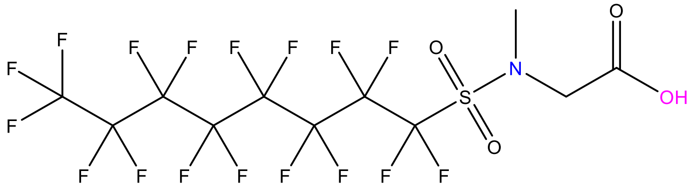	Log K_{OW} = 5.00<br>Log S = 0.89<br>Log K_{OC} = 3.44	pK_{a1} = 3.9 *<br>pK_{a2} = N.A.
EtFOSAA	2991-50-6	Sulfonamido Acetic acid (FOSAA)	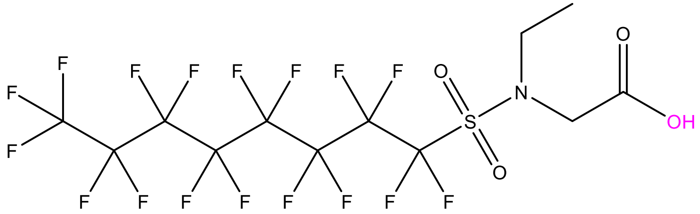	Log K_{OW} = 5.40<br>Log S = 0.38<br>Log K_{OC} = 3.71	pK_{a1} = 3.9 [7]<br>pK_{a2} = N.A.

**PFAS acronym**	**CAS number**	**Subfamily**	**Chemical structure**	**Log K_{OW}**<br>**Log *S***<br>**Log K_{OC}**	**pKa_1**<br>**pKa_2**
PFHxPA	40143-76-8	Phosphonate (PFPA)	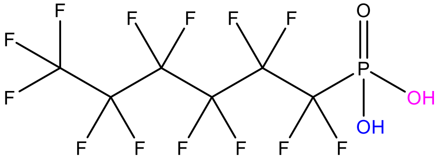	Log K_{OW} = 3.00<br>Log S = 3.87<br>Log K_{OC} = 2.72	pK_{a1} = 2.1 [8]<br>pK_{a2} = 2.4 [8]
PFOPA	40143-78-0	Phosphonate (PFPA)	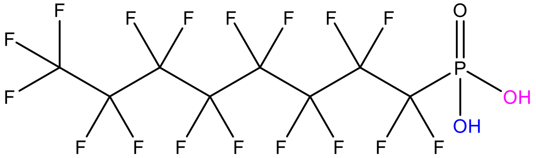	Log K_{OW} = 4.30<br>Log S = 2.10<br>Log K_{OC} = 3.46	pK_{a1} = 2.4 [8]<br>pK_{a2} = 4.5 [8]
PFDPA	52299-26-0	Phosphonate (PFPA)	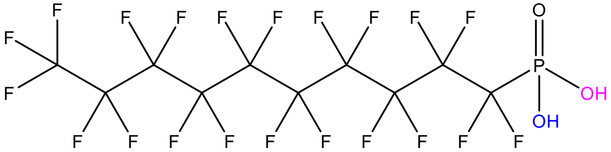	Log K_{OW} = 5.60<br>Log S = 0.30<br>Log K_{OC} = 4.20	pK_{a1} = 3.4 [8]<br>pK_{a2} = 5.6 [8]
C6/6 PFPiA	40143-77-9	Phosphinate (PFPiA)	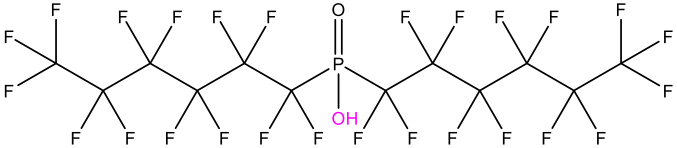	Log K_{OW} = 8.00<br>Log S = -1.41<br>Log K_{OC} = 4.88	pK_{a1} = 0.9 [9]<br>pK_{a2} = N.A.
C6/8 PFPiA	158986-67-5	Phosphinate (PFPiA)	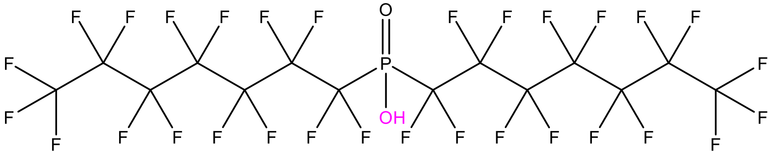	Log K_{OW} = 9.40<br>Log S = -3.23<br>Log K_{OC} = 5.62	pK_{a1} = 0.9 *<br>pK_{a2} = N.A.
C8/8 PFPiA	40143-79-1	Phosphinate (PFPiA)	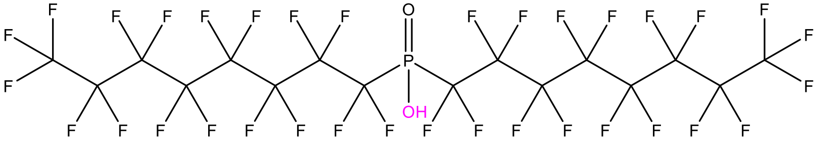	Log K_{OW} = 10.7<br>Log S = -5.05<br>Log K_{OC} = 6.36	pK_{a1} = 0.9 *<br>pK_{a2} = N.A.

Note: K_{OW}: octanol-water partition coefficient [-]; *S*: PFAS solubility [μg L^{-1}] resulting from EPISuite; K_{OC} = organic-carbon normalized sorption coefficient; *: based on other similar chemical structures.

S2: pH-speciation diagrams for some representative PFAS species

6:2 FTAB

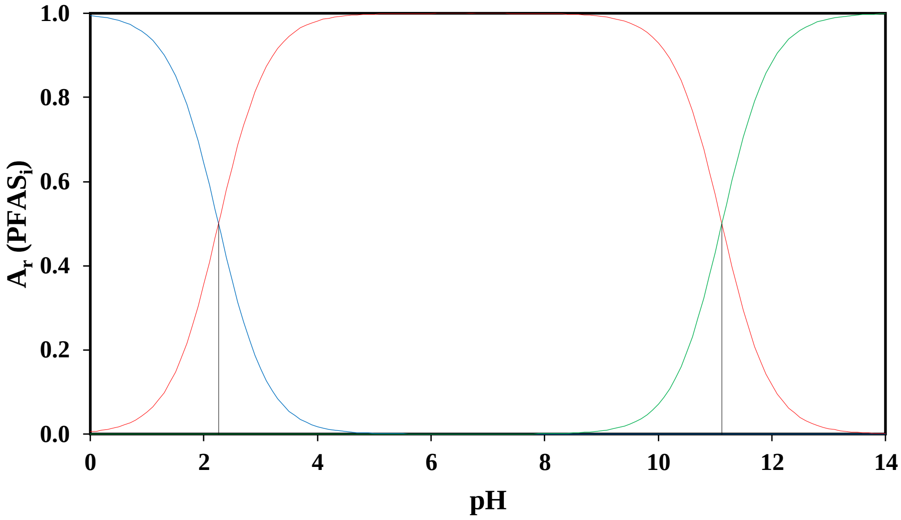

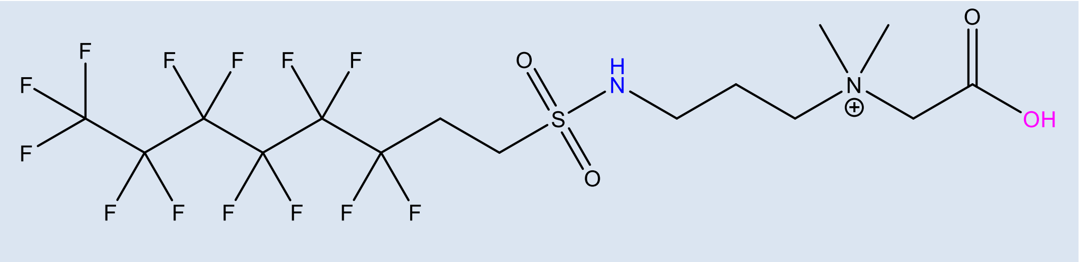

Fraction f (+)
z = +1

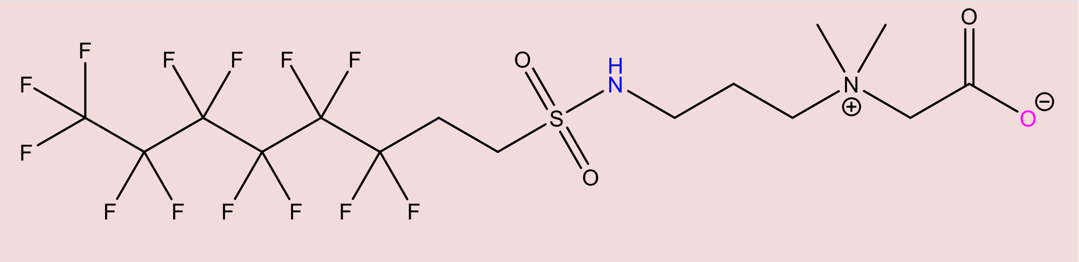

Fraction f (+/-)
z = 0

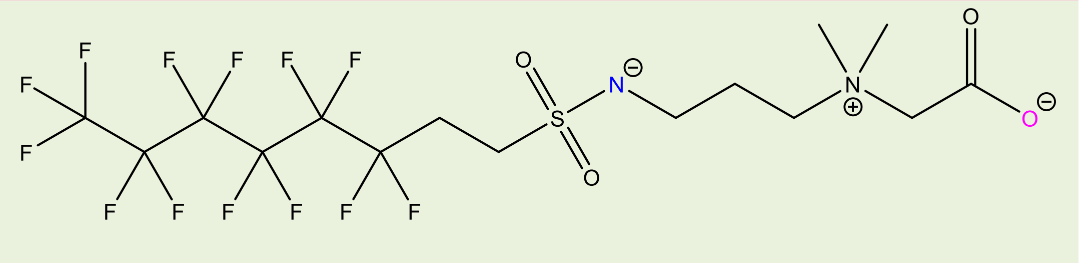

Fraction f (-/+/-)
z = -1

Z indicates molar net charge [-]; A_r *($PFAS_i$)* indicate the relative abundance of each PFAS specie *i*

6:2 FtSaAm

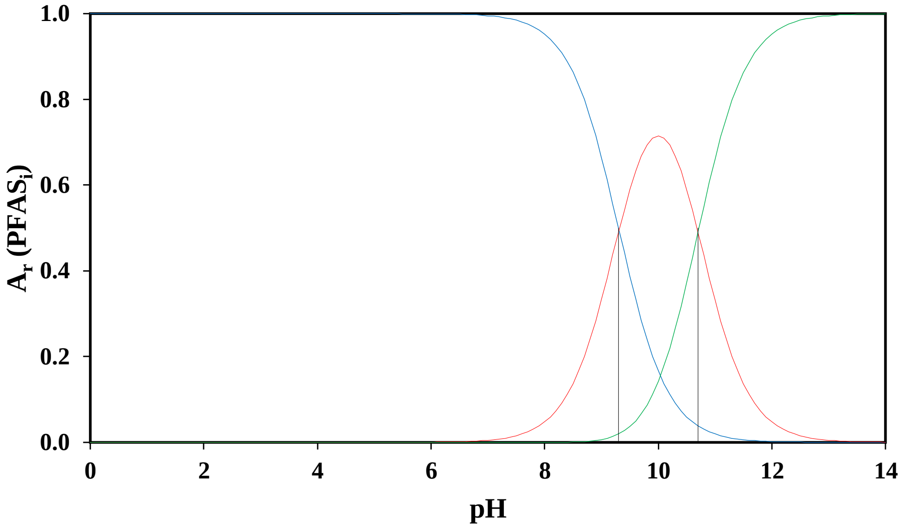

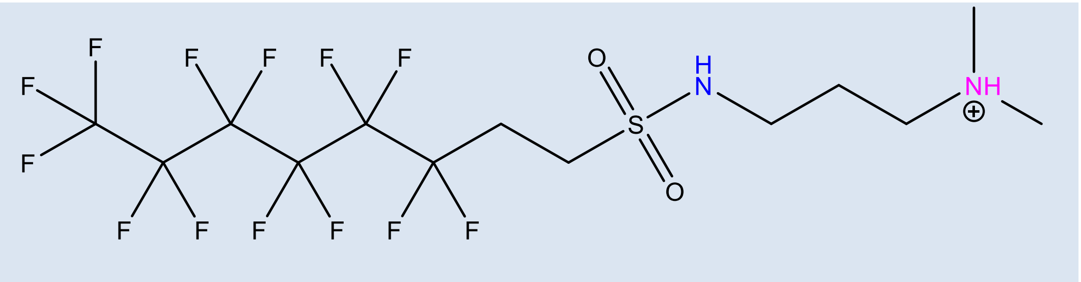

Fraction f (+)
z = +1

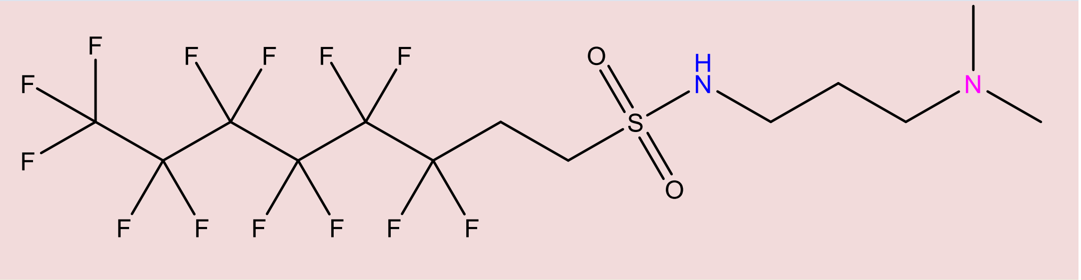

Fraction f (+/-)
z = 0

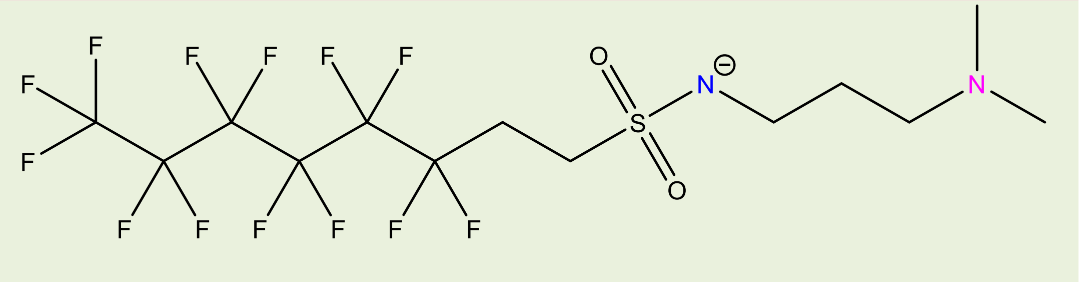

Fraction f (-/+/-)
z = -1

Z indicates molar net charge [-]; A_r *(PFAS$_i$)* indicate the relative abundance of each PFAS specie *i*

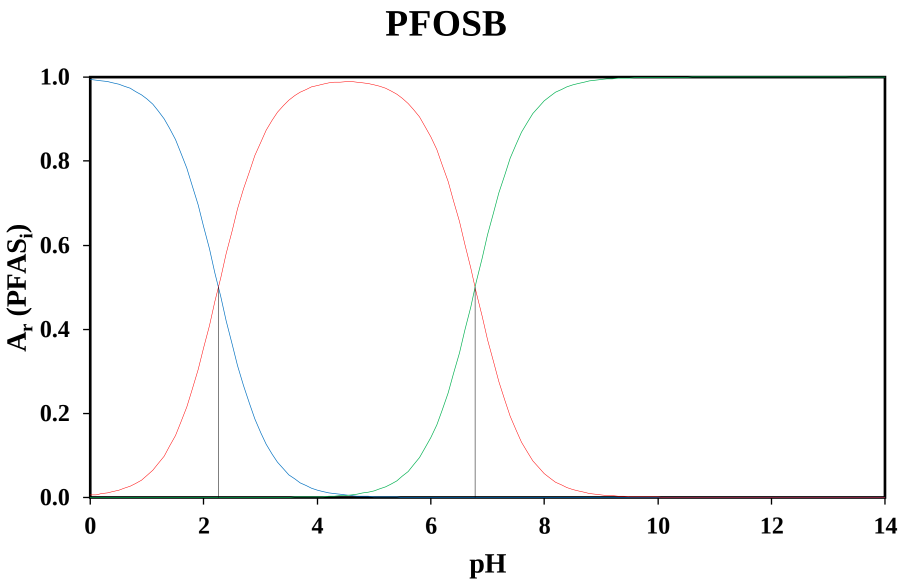

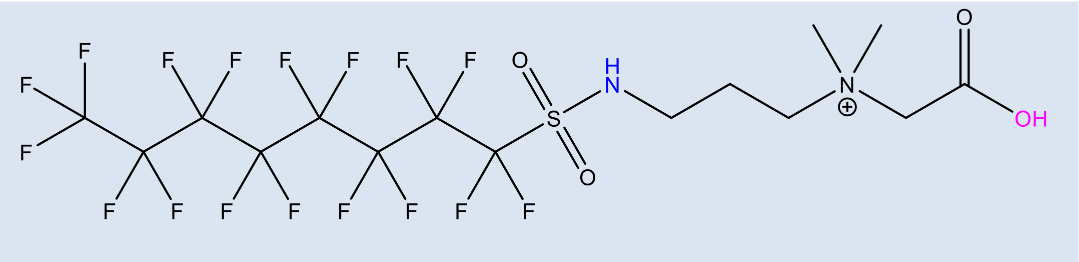

Fraction f (+)
z = +1

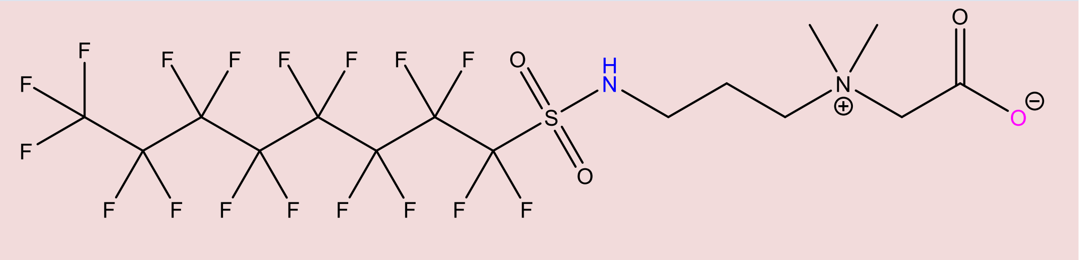

Fraction f (+/-)
z = 0

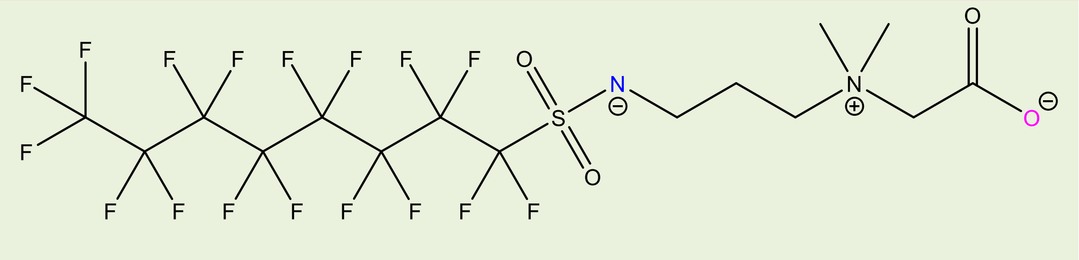

Fraction f (-/+/-)
z = -1

Z indicates molar net charge [-]; A_r *($PFAS_i$)* indicate the relative abundance of each PFAS specie *i*

PFOAAmS

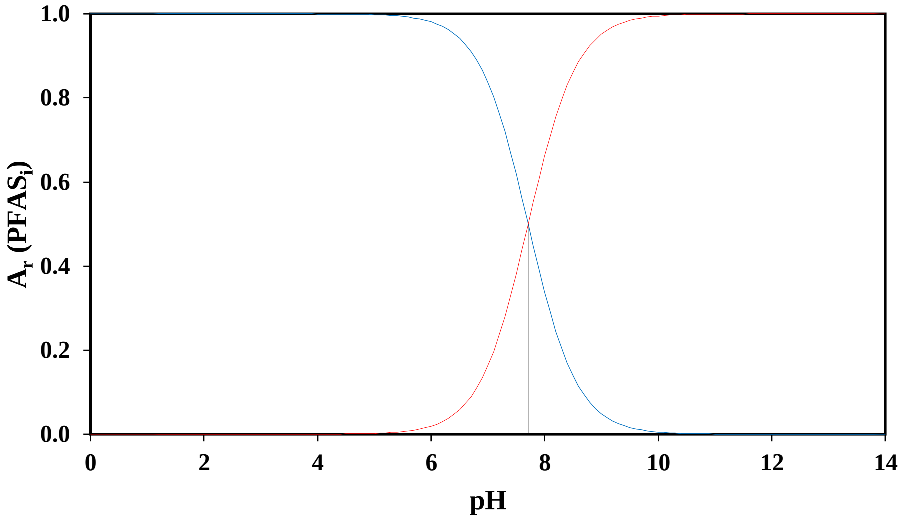

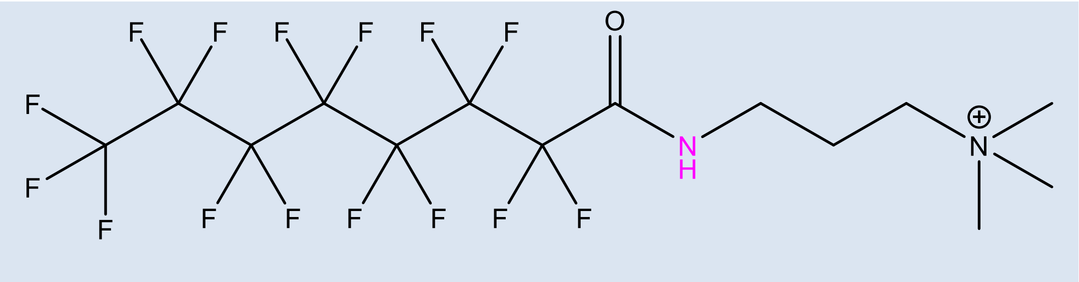

Fraction f (+)
z = +1

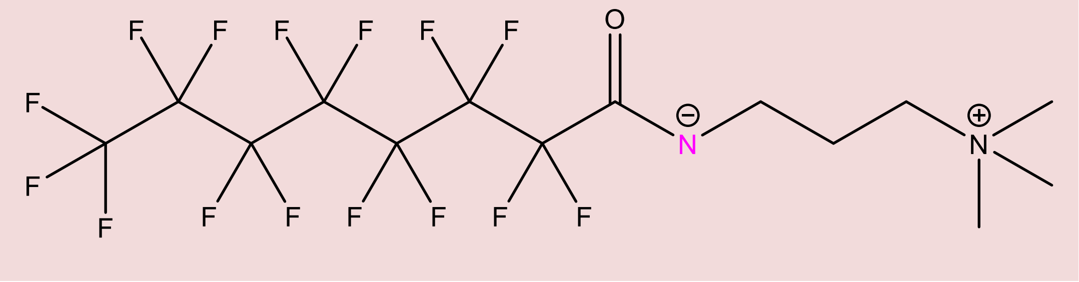

Fraction f (+/-)
z = 0

Z indicates molar net charge [-]; A_r *(PFAS$_i$)* indicate the relative abundance of each PFAS specie *i*

PFOAB

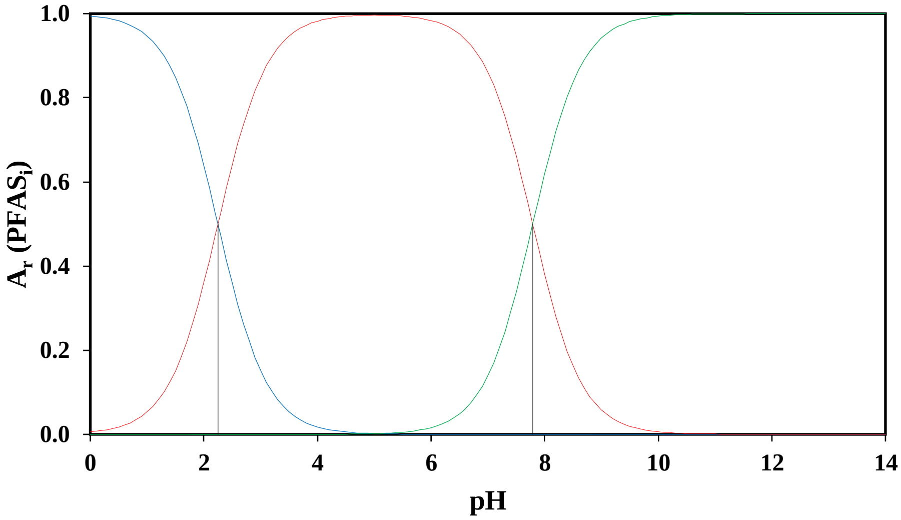

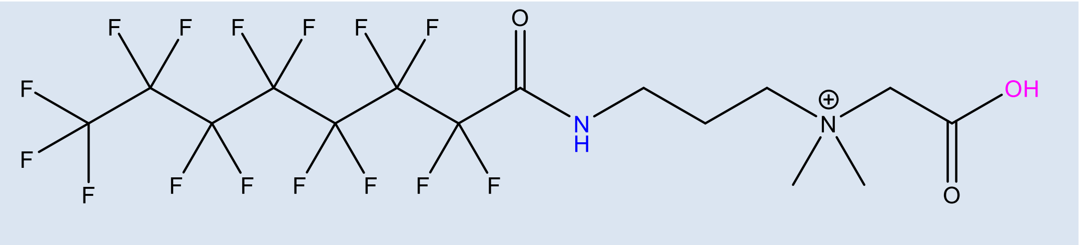

Fraction f (+)
z = +1

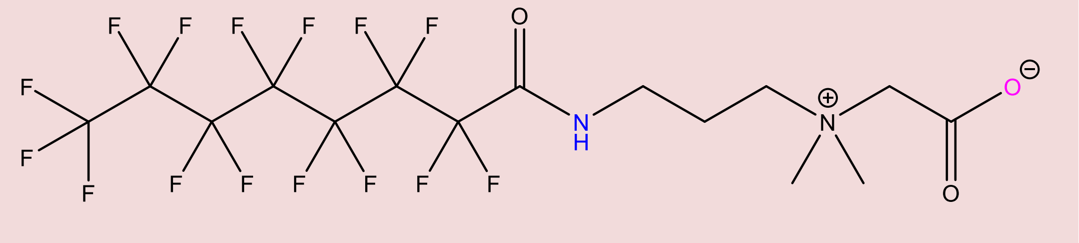

Fraction f (+/-)
z = 0

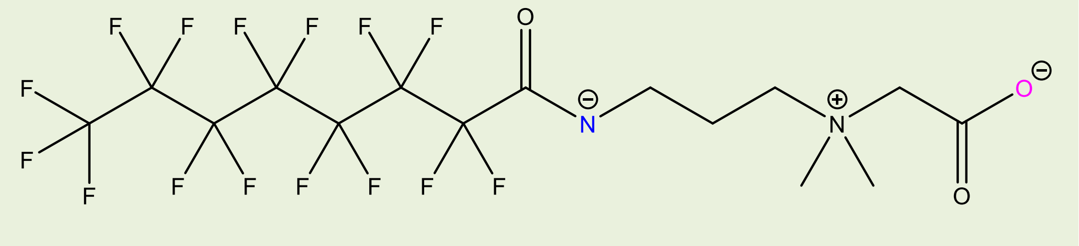

Fraction f (-/+/-)
z = -1

Z indicates molar net charge [-]; A_r *(PFAS$_i$)* indicate the relative abundance of each PFAS specie *i*

AmPr-FHxSA

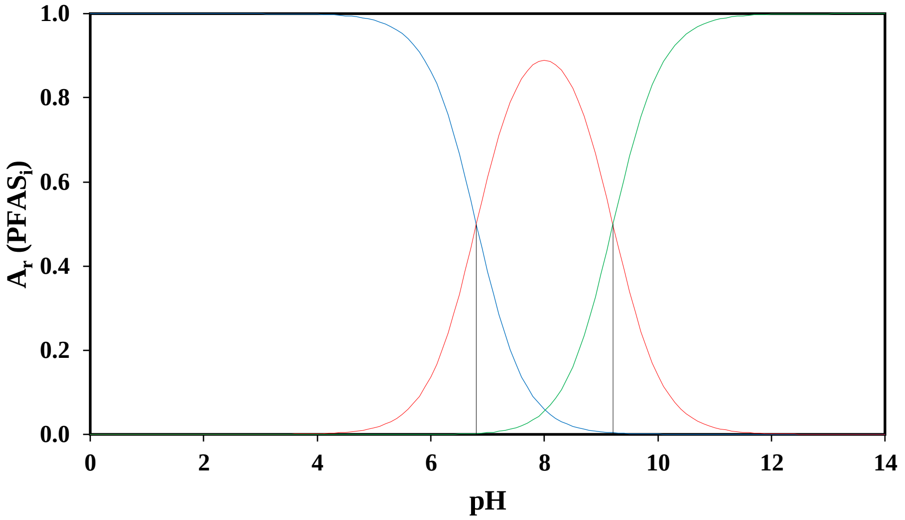

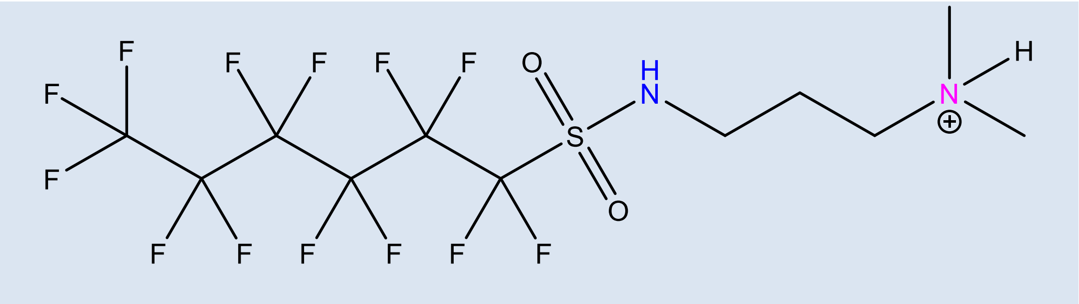

Fraction f (+)
z = +1

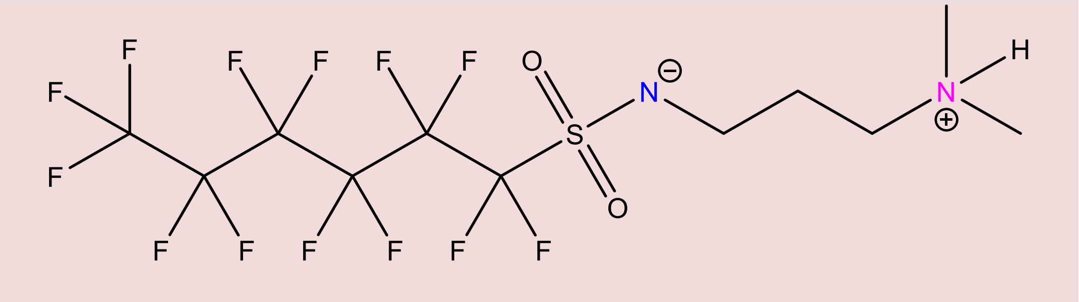

Fraction f (+/-)
z = 0

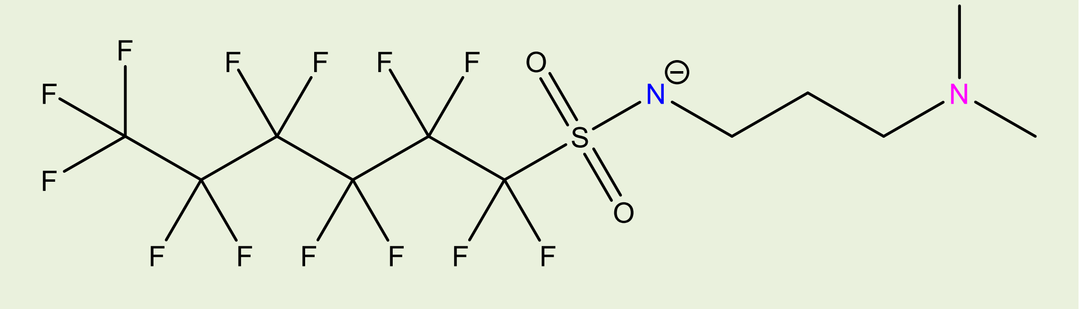

Fraction f (-)
z = -1

Z indicates molar net charge [-]; A_r *(PFAS$_i$)* indicate the relative abundance of each PFAS specie *i*

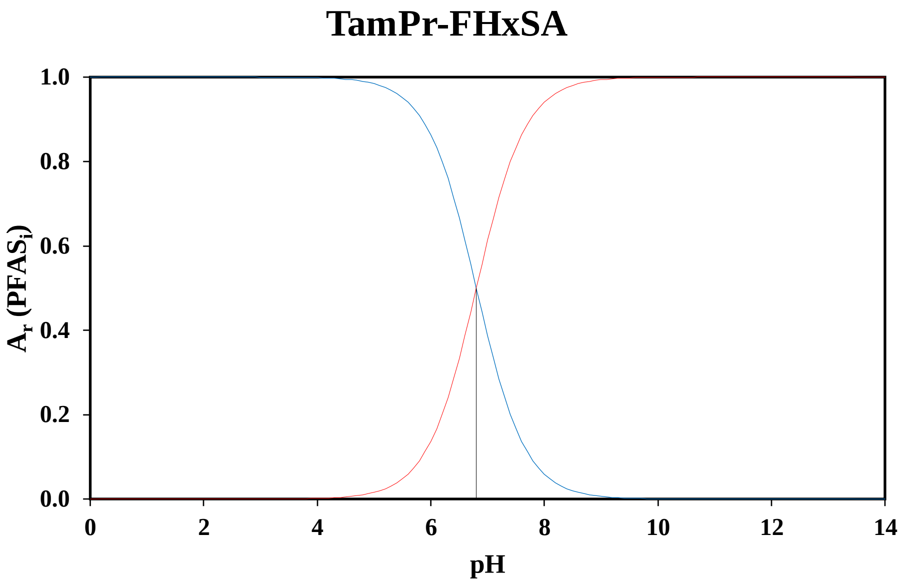

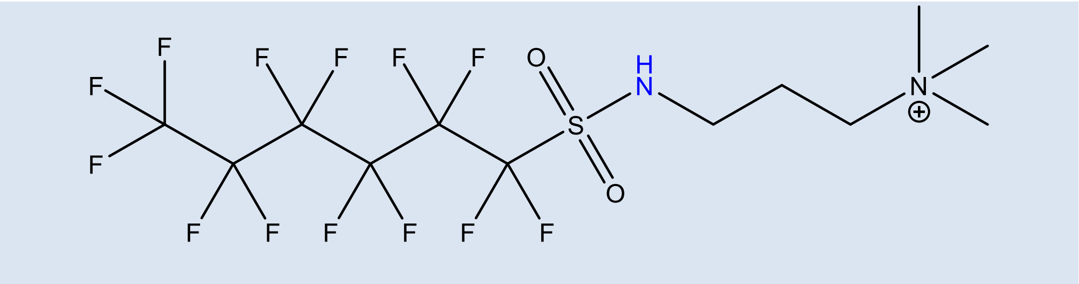

Fraction f (+)
z = +1

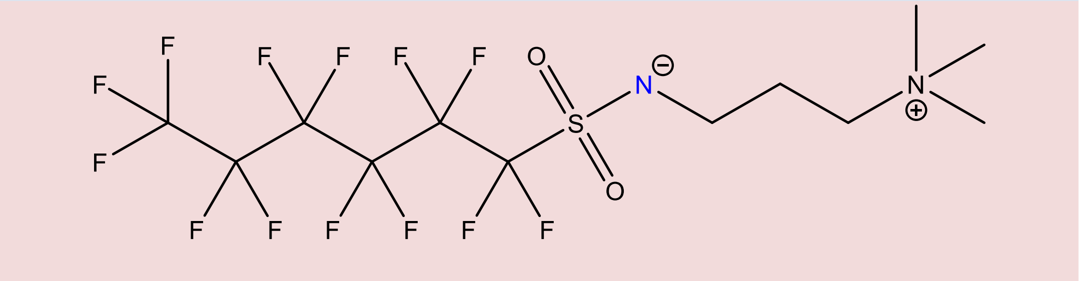

Fraction f (+/-)
z = 0

Z indicates molar net charge [-]; A_r *(PFAS$_i$)* indicate the relative abundance of each PFAS specie *i*

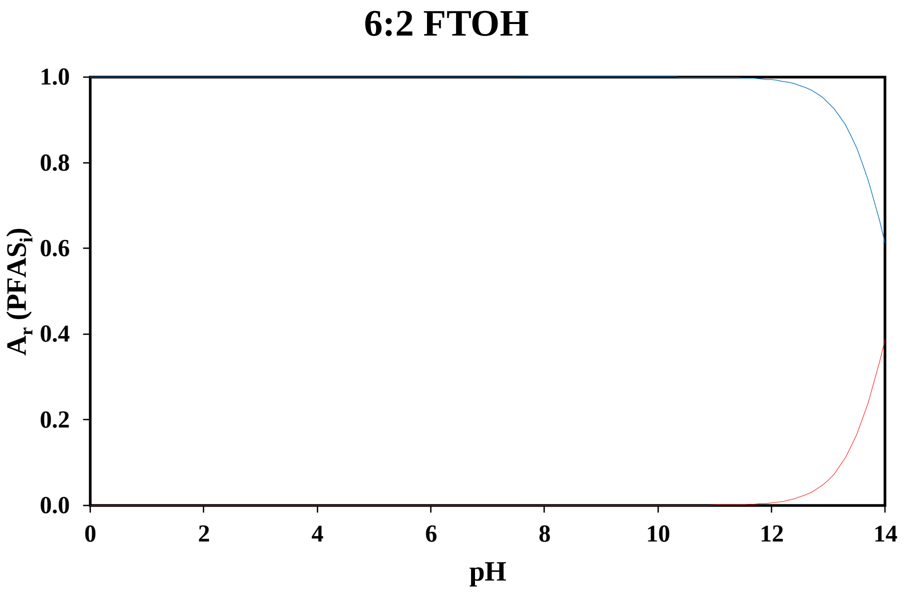

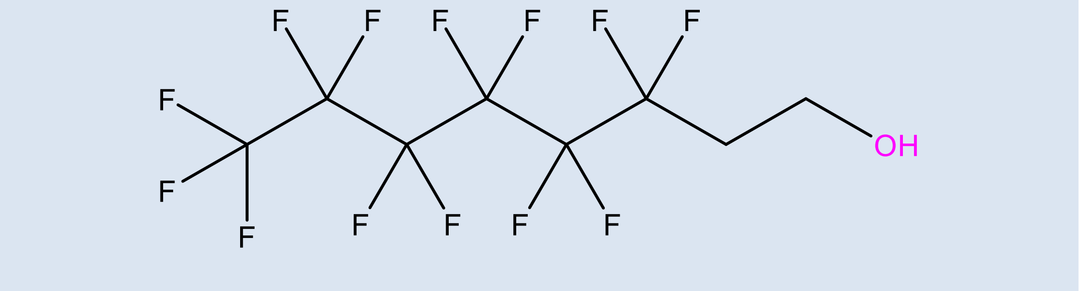

Fraction f (0)
z = 0

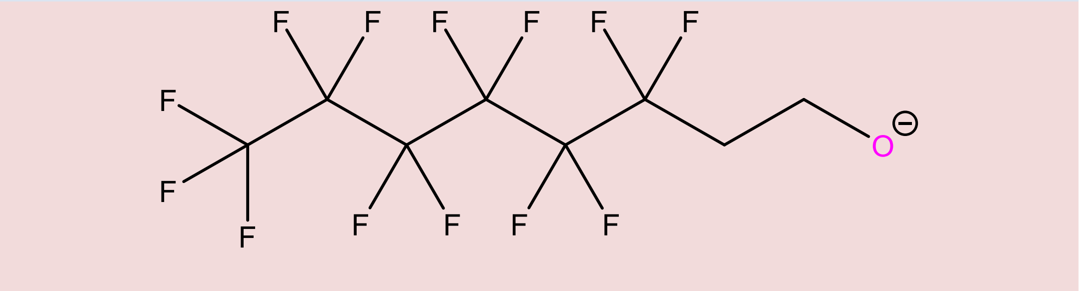

Fraction f (-)
z = -1

Z indicates molar net charge [-]; A_r *($PFAS_i$)* indicate the relative abundance of each PFAS specie *i*

PFOA

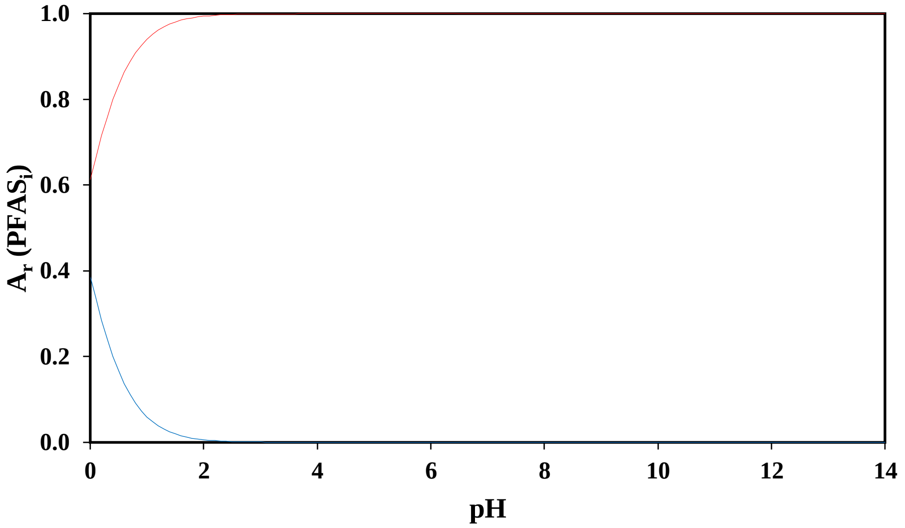

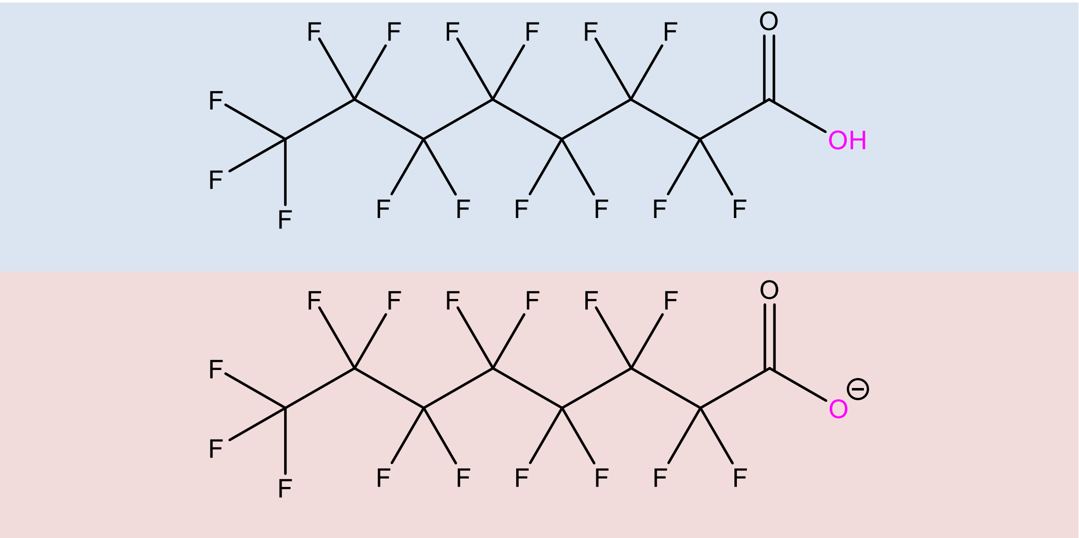

Fraction f (0)
z = 0

Fraction f (-)
z = -1

Z indicates molar net charge [-]; A_r *($PFAS_i$)* indicate the relative abundance of each PFAS specie *i*

PFOS

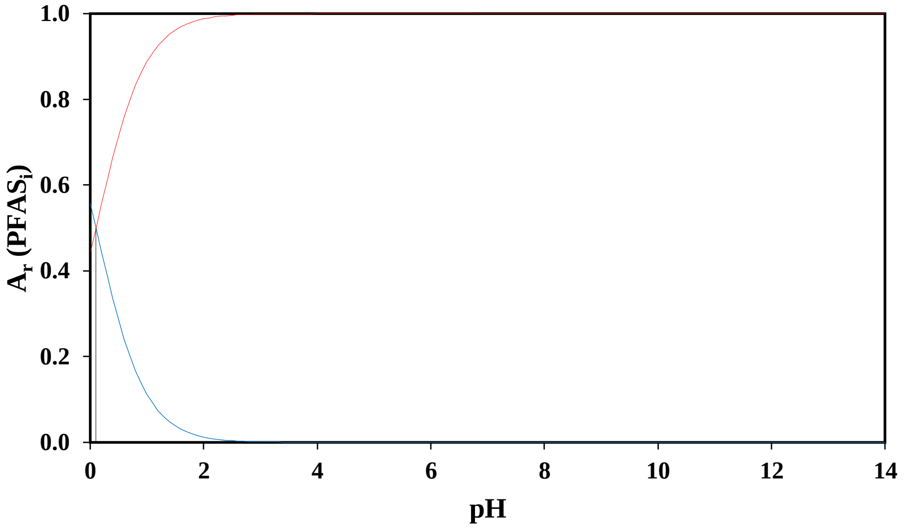

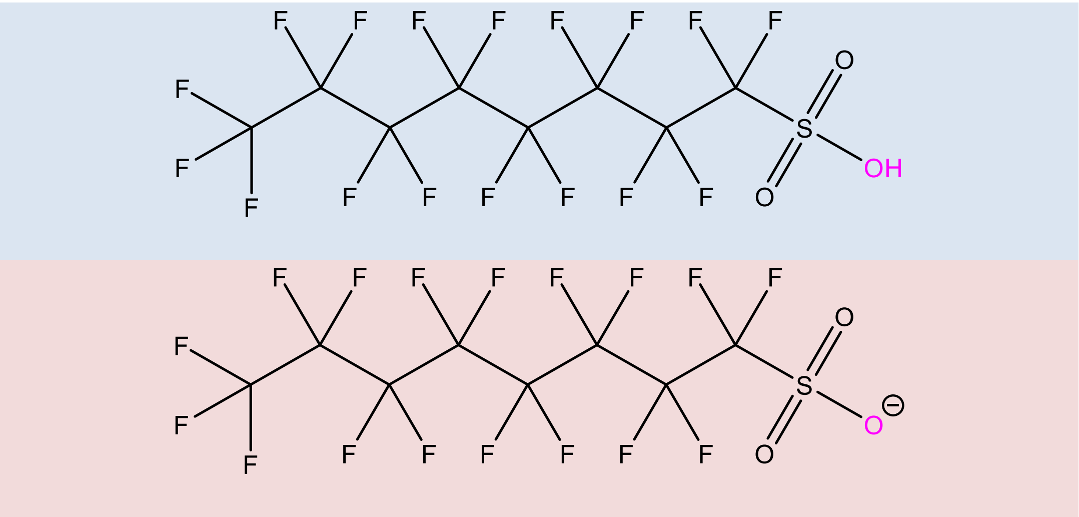

Fraction f (0)
z = 0

Fraction f (-)
z = -1

Z indicates molar net charge [-]; A_r *($PFAS_i$)* indicate the relative abundance of each PFAS specie *i*

6:2 FTS

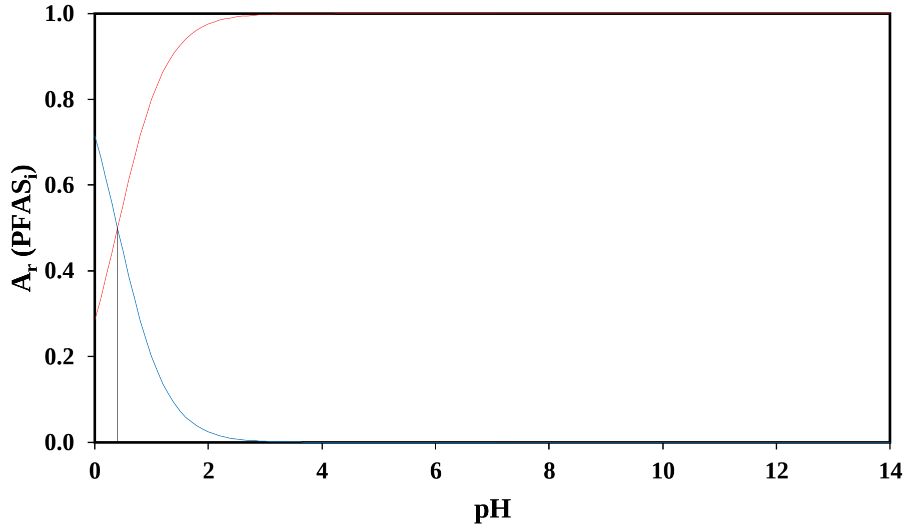

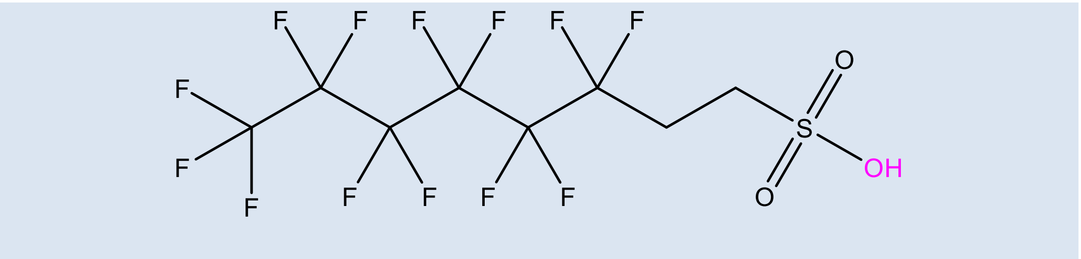

Fraction f (0)
z = 0

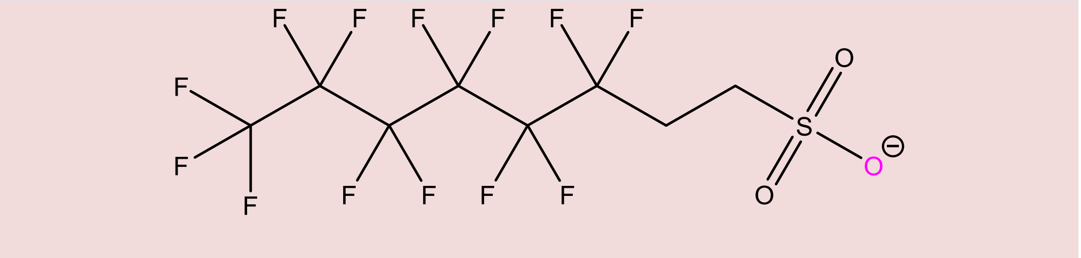

Fraction f (-)
z = -1

Z indicates molar net charge [-]; A_r *(PFAS$_i$)* indicate the relative abundance of each PFAS specie *i*

PFOSA

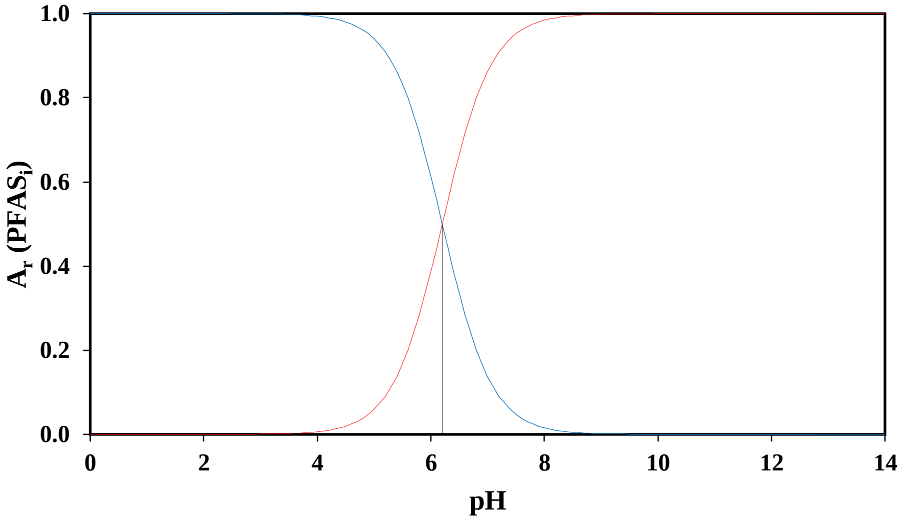

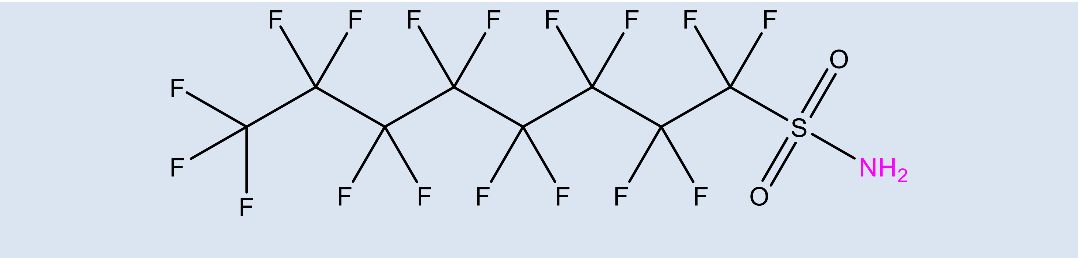

Fraction f (0)
z = 0

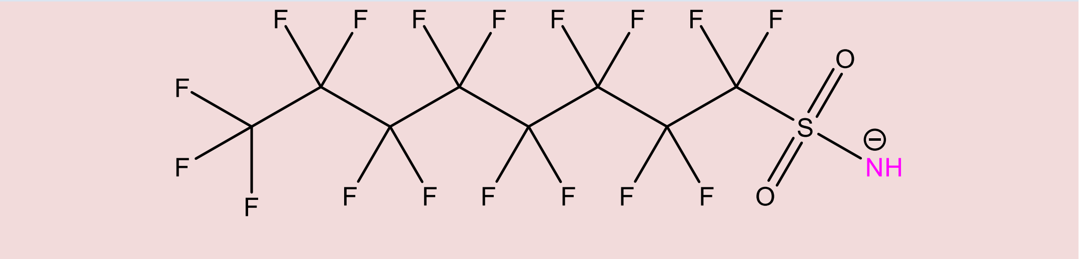

Fraction f (-)
z = -1

Z indicates molar net charge [-]; A_r *($PFAS_i$)* indicate the relative abundance of each PFAS specie *i*

EtFOSA

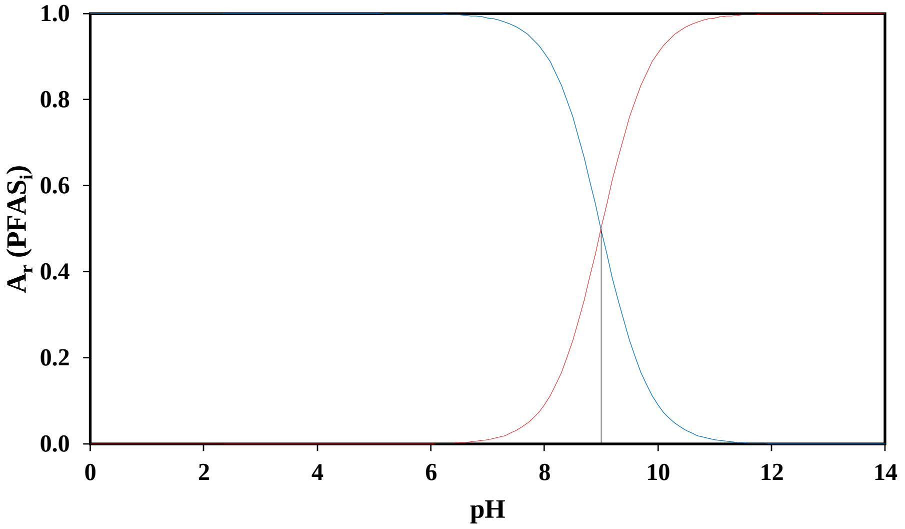

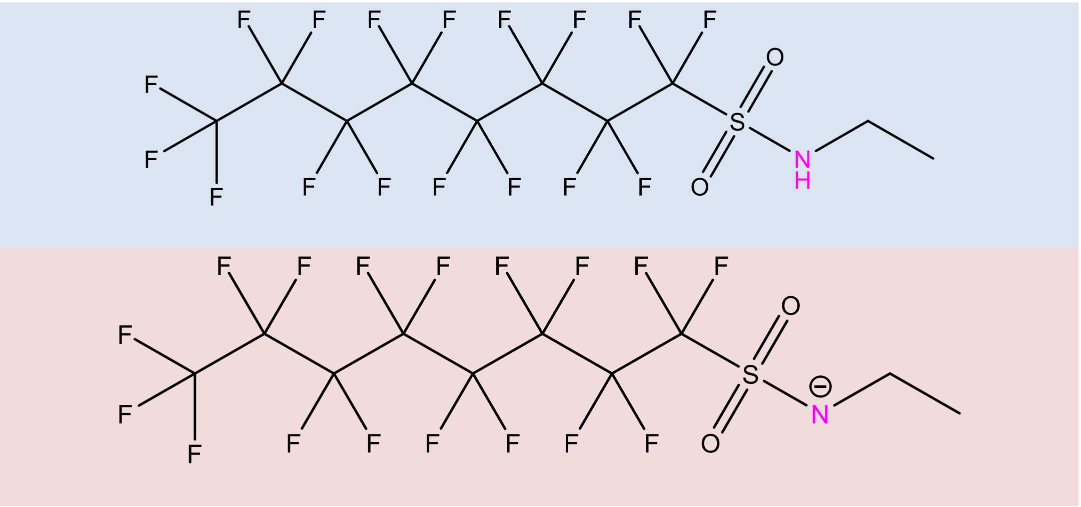

Fraction f (0)
z = 0

Fraction f (-)
z = -1

Z indicates molar net charge [-]; A_r *($PFAS_i$)* indicate the relative abundance of each PFAS specie *i*

N-EtFOSAA

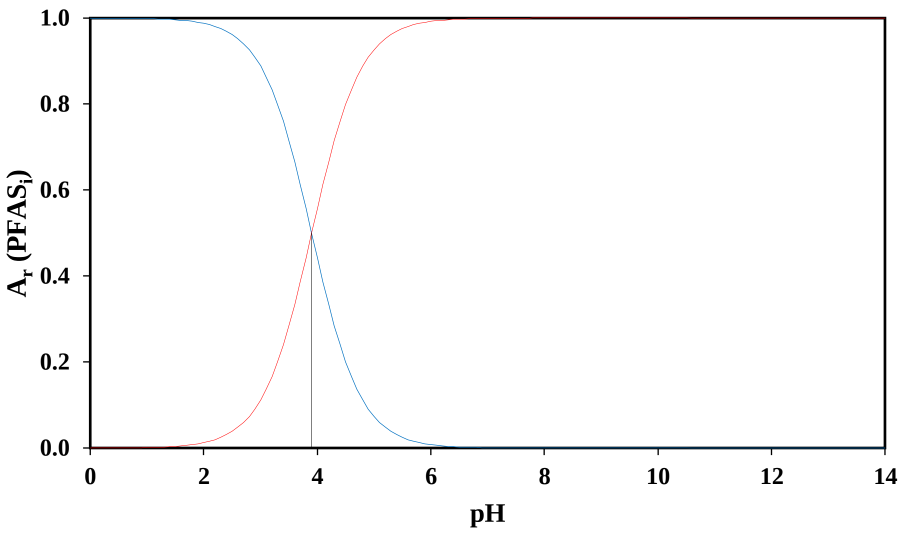

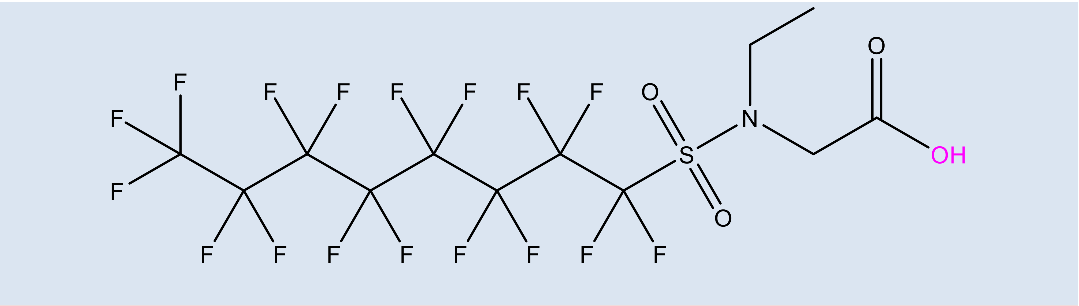

Fraction f (0)
z = 0

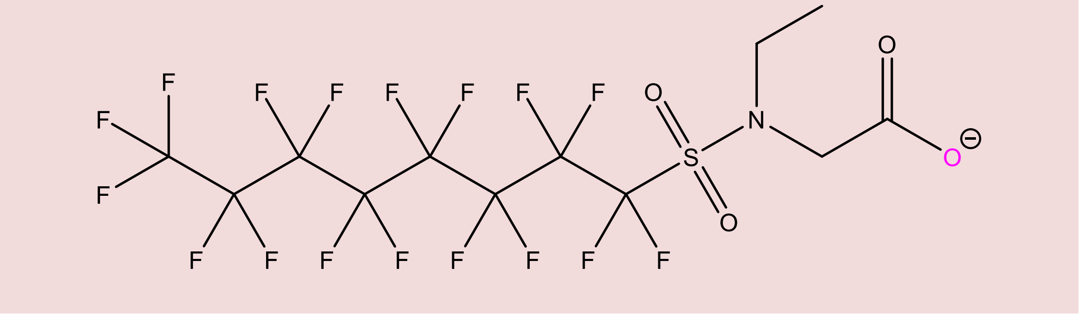

Fraction f (-)
z = -1

Z indicates molar net charge [-]; A_r *(PFAS$_i$)* indicate the relative abundance of each PFAS specie *i*

PFOPA

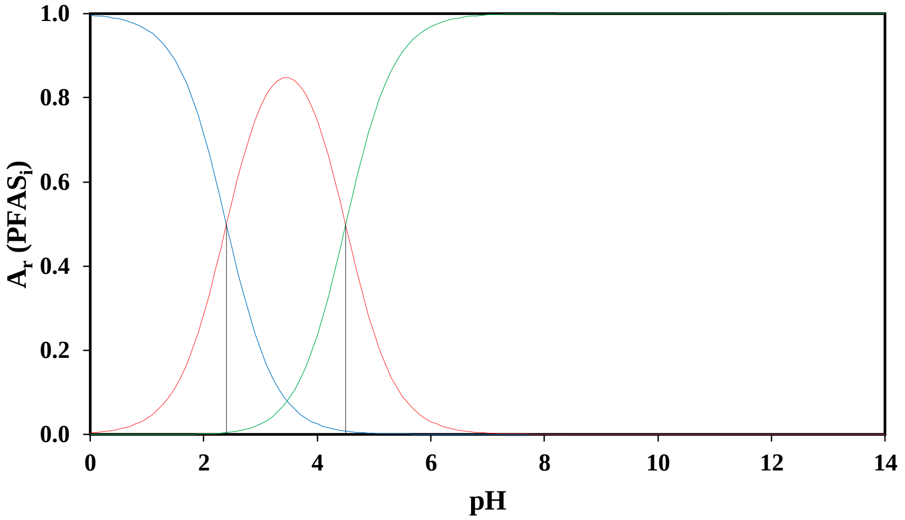

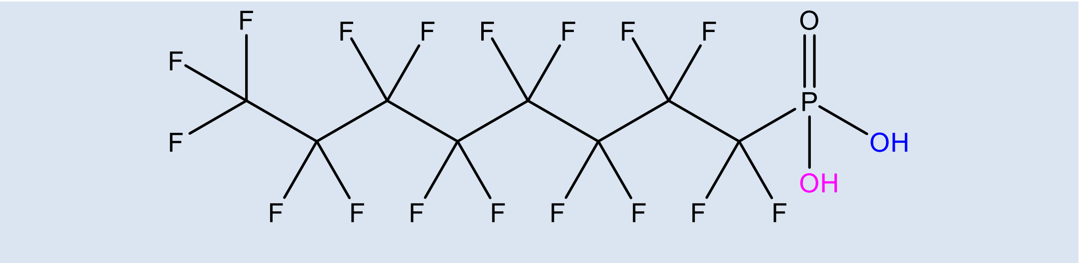

Fraction f (0)
z = 0

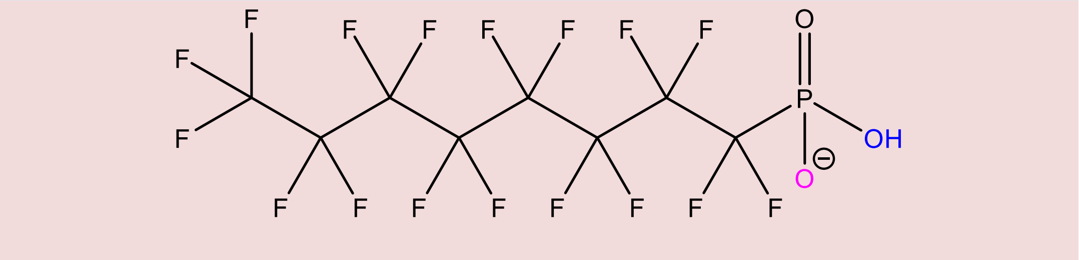

Fraction f (-)
z = -1

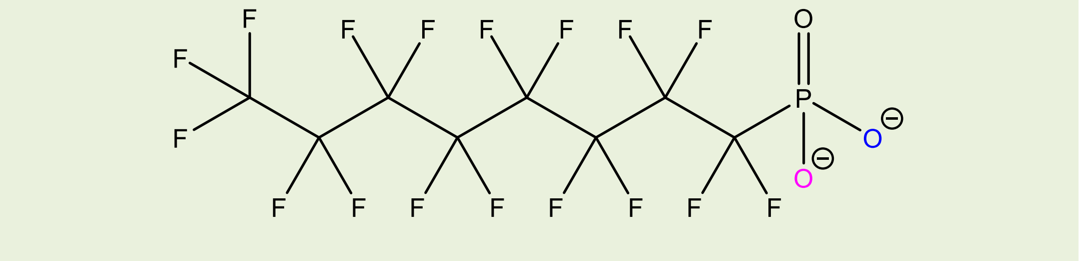

Fraction f (-/-)
z = -2

Z indicates molar net charge [-]; A_r *(PFAS$_i$)* indicate the relative abundance of each PFAS specie *i*

$C_{6/6}$ PFPiA

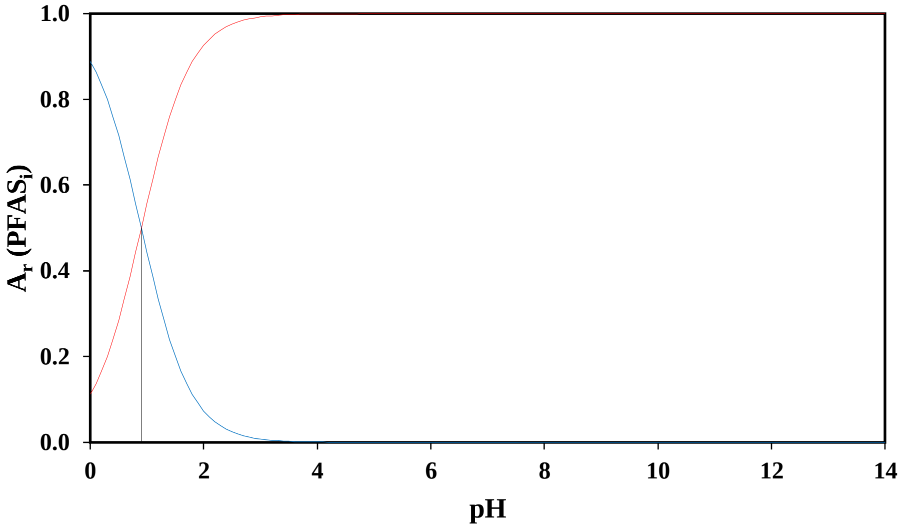

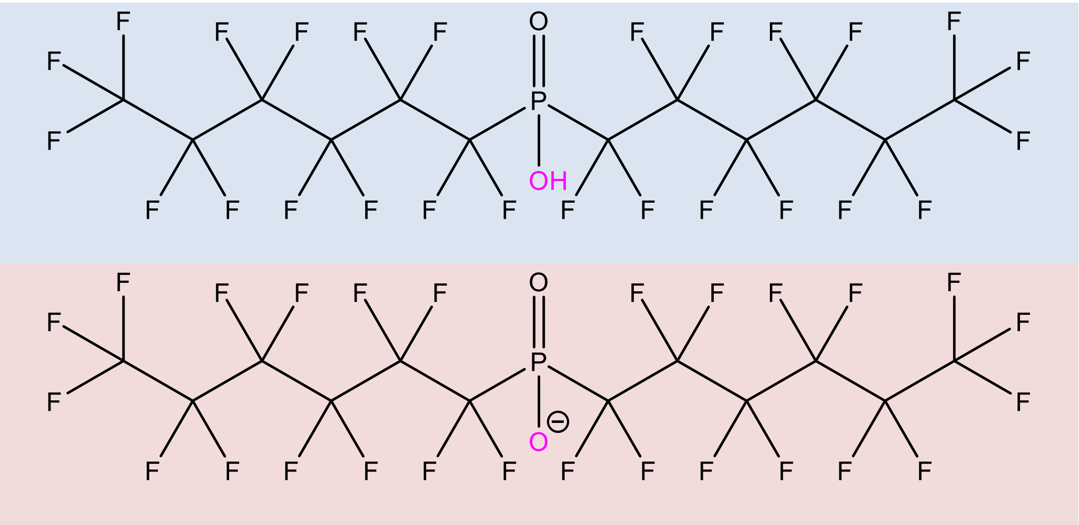

Fraction f (0)
z = 0

Fraction f (-)
z = -1

Z indicates molar net charge [-]; A_r *($PFAS_i$)* indicate the relative abundance of each PFAS specie *i*

S3: Additional information on the derivation of K_d from literature

The solid-liquid distribution coefficient K_d [L kg^{-1}] is defined as the ratio between the sorbed pollutant concentration in the solid phase C_S [mg kg^{-1}] to the non-sorbed pollutant concentration in the water phase C_W [mg L^{-1}] under equilibrium conditions, as shown in Equation S1:

$$K_d = \frac{C_S}{C_W} \quad \text{(S1)}$$

K_d is therefore a parameter suitable to assess the concurrent equilibria for pollutants between solid and liquid phases in the environment, and a relevant input parameter used in contaminant transport modelling [10], with higher K_d values attributed to higher sorption to soil particles and therefore, a higher retardation on contaminant transport to groundwater. Specifically, for PFAS studies assessing sorption in soils, K_d values have a wide range of variability. For instance, K_d values ranging from < 0.1 to 10 L kg^{-1} have been reported for short-chained PFAS such as TFA and PFBA [11,12], while K_d values ranging from 90 to 5,700 L kg^{-1} have been reported for long-chained PFAS such as PFDoA [11,13]. K_d values reported for PFOA range from < 1 to 130 L kg^{-1} [11,14]. These discrepancies in observed K_d values are a result of the different soil properties, especially the differing organic carbon (C_{ORG}) content. As such, K_d values are usually normalized to the fraction of soil organic carbon (f_{OC}), thus defining the organic-carbon normalized sorption coefficient K_{OC} [L kg OC^{-1}] as in Equation S2:

$$K_{OC} = \frac{K_d}{f_{OC}} \quad \text{(S2)}$$

The normalization of K_d to K_{OC} is suitable to reduce K_d variability for compounds whose main sorption domain is C_{ORG} [14]. In the case of PFAS, however, low but significant sorption is observed in pure inorganic minerals such as kaolinite and soils with low C_{ORG} [15,16] which may lead to a plausible overestimation of K_{OC} in low C_{ORG} scenarios. Although these overestimations may be overcome by the inclusion of a term in Equation S2 representative of the sorption in reactive mineral

components [14], in this work, we derived individual PFAS/sorbent K_{OC} values for each entry in the dataset of Supplementary File 1 according to Equation S2 to assess the role of PFAS chain length and functional group on sorption.

Sorption isotherms describe the relationship between C_S and C_W over a certain concentration range. The resulting isotherm shape may be linear (where the slope, *i.e.*, K_d, is independent of the tested concentration) or non-linear (where the slope, *i.e.*, K_d, is dependent on the tested concentration). Non-linear isotherms are often described by the Freundlich model (Equation S3):

$$C_S = K_F \, C_W^{1/n} \tag{S3}$$

Where K_F is the Freundlich constant [(mg kg^{-1}) / (mg L^{-1})$^{1/n}$], representative of the sorbent-sorbate sorption affinity, and *1/n* being representative of a non-linearity term. For those sorbents were the sorption site availability is limited in respect to the number of pollutant molecules in the system, often a non-linearity term with $1/n < 1$ is observed, and K_d decrease with increasing pollutant concentration. On the other hand, cooperative sorption (*i.e.*, additional sorption sites provided by sorbed pollutants due to pollutant – pollutant interactions) may result in non-linear isotherms with $1/n > 1$, where K_d increase with increasing pollutant concentration. Regarding PFAS, all linear (*i.e.,* $1/n = 1$) and non-linear isotherm *(i.e.,* $1/n < 1$ and $1/n > 1$) have been observed [1,17,18].

Non-linear sorption isotherms may also be described by the Langmuir model (Equation S4) when a complete saturation of sorption sites occurs. The model allows to derive the Langmuir constant K_L [L mg^{-1}], representative of the sorbent-sorbate sorption affinity, and the maximum loading capacity Q_{MAX} [mg kg^{-1}], representative of the total number of sorption sites beard by the sorbent.

$$C_S = \frac{K_L \, Q_{MAX} \, C_W}{1 + K_L \, C_W} \tag{S4}$$

Regarding PFAS, only a few studies observed sorption isotherms well described by the Langmuir model [19].

To extend the pool of K_d (PFAS) data in our dataset, we carefully examined literature studies reporting fitted isotherm data but not providing K_d. Similar approaches have been applied elsewhere [20–24]. In some of these works [20–22], K_d values were derived from both Freundlich and Langmuir models at a certain arbitrary C_W that fell in the low concentration range of the sorption isotherm, aiming to represent low environmental concentrations [25] and assuming that K_d data fall in the linear range of the sorption isotherm. On the other hand, recent studies have derived K_d data from literature studies resulting from fitted isotherm parameters at the same C_W for all PFAS, allowing a FAIR comparison [23]. Nonetheless, this approach results in the derivation of K_d values for PFAS at very different activities in water, given the differences in solubility values for short and long-chained PFAS (see Table S1). To address this, some other studies have derived K_d data from literature resulting from fitted isotherm parameters at the same PFAS activity in water (i.e., C_W = 10% of PFAS solubility), which allowed a better comparison of K_d values among sorbates [24]. In this work, we applied this latter approach, deriving K_d values from reported isotherm data fitted to both the Freundlich and Langmuir models at different C_W but at the same PFAS activity in water (*i.e.*, C_W = 10% of PFAS solubility). Care was taken to consistently derive solubility values from the same source (*i.e.*, EPISuite) [26]. The assessment of whether these values differed among experimental [27] or other modeled (e.g., COSMOtherm) [28] data was out of the scope of this work.

S4: Number of entries and references used to derive log K_{OC} values

Table S2. Summary of the total number of entries and references for each PFAS compound included in the dataset, along with respective averaged log K_{OC} values calculated as Equation S2. Values in brackets are the standard deviation; values with * are considered as outliers (and therefore not considered in the training set). N.A.: Not Applicable.

PFAS	Subfamily	Number of entries	Number of references	Average log K_{OC}
TFA	PFCA	20	1	1.18 (0.37)
PFBA	PFCA	24	7	1.33 (0.61)
PFPeA	PFCA	19	6	1.48 (0.49)
PFHxA	PFCA	26	7	1.47 (0.47)
PFHpA	PFCA	24	9	1.91 (0.46)
PFOA	PFCA	187	26	2.57 (0.70)
PFNA	PFCA	34	12	2.55 (0.37)
PFDA	PFCA	30	9	3.14 (0.52)
PFUnA	PFCA	20	6	3.71 (0.48)
PFDoA	PFCA	21	5	4.50 (0.67)
PFTrA	PFCA	3	2	4.77 (0.57)
PFTeA	PFCA	2	1	5.25 (0.70)
GenX	PFECA	10	1	1.71 (0.53)
ADONA	PFECA	10	1	1.69 (0.46)
PFBS	PFSA	34	11	1.43 (0.47)
PFPeS	PFSA	12	2	1.71 (0.45)
PFHxS	PFSA	72	11	1.82 (0.46)
PFHpS	PFSA	12	3	2.62 (0.31)
PFOS	PFSA	238	32	3.07 (0.39)
PFNS	PFSA	12	2	3.39 (0.42)
PFDS	PFSA	16	3	3.86 (0.49)
PFEtCHxS	PFSA	10	1	2.47 (0.44)
9Cl-PF3ONS	PFAES	10	1	4.05 (0.51)
FBSA	FOSA	10	1	1.71 (0.49)
FHXSA	FOSA	10	1	2.32 (0.62)
PFOSA	FOSA	16	3	3.37 (0.61)
EtFOSA	FOSA	3	1	3.39 (0.48)
N-MeFOSAA	FOSAA	5	1	3.10 (0.31)
N-EtFOSAA	FOSAA	5	1	3.33 (0.35)
4:2 FTOH	FTOH	1	1	0.93 (N.A.)
6:2 FTOH	FTOH	3	1	2.42 (0.10)
8:2 FTOH	FTOH	5	1	3.77 (0.04)
10:2 FTOH	FTOH	3	1	4.60 (0.28)
4:2 FTS	FTS	10	1	1.69 (0.49)
6:2 FTS	FTS	27	6	2.14 (0.67)
8:2 FTS	FTS	26	5	3.01 (0.71)
PFHxPA	PFPA	7	1	1.69 (0.55)
PFOPA	PFPA	7	1	2.64 (0.39)
PFDPA	PFPA	7	1	2.77 (0.48)
C6/6 PFPiA	PFPiA	7	1	3.19 (0.49) *
C6/8 PFPiA	PFPiA	7	1	3.56 (0.73) *
C8/8 PFPiA	PFPiA	7	1	2.53 (0.80) *
6:2 FtSaB	Zwitterionic	22	4	2.75 (1.12)
8:2 FtSaB	Zwitterionic	5	1	4.14 (1.06)
10:2 FtSaB	Zwitterionic	2	1	4.54 (0.34)
6:2 FtSaAm	Cationic	1	1	5.93 (N.A.) *
PFOSB	Zwitterionic	5	1	2.46 (0.76)
PFOAAmS	Cationic	11	3	3.31 (0.50)
PFOAB	Zwitterionic	11	3	2.30 (0.60)
AmPr-FHSA	Cationic	10	1	3.26 (0.88)
TAmPr-FHSA	Cationic	10	1	3.47 (1.10)

S5: Construction of a soil property imputer model based on KNN

To address gaps in our dataset for soil properties, needed to predict PFAS sorption, we used data from SoilGrids250m v2.0 (https://soilgrids.org/) [29] to develop a K-nearest neighbor (KNN) imputation model. The number of soils considered was 2,039, with information on soil pH, C_{ORG}, CEC, and soil texture for each sampling coordinate. The soils were distributed worldwide, with a higher number of data entries for USA, South America, and African soils (Figure S1).

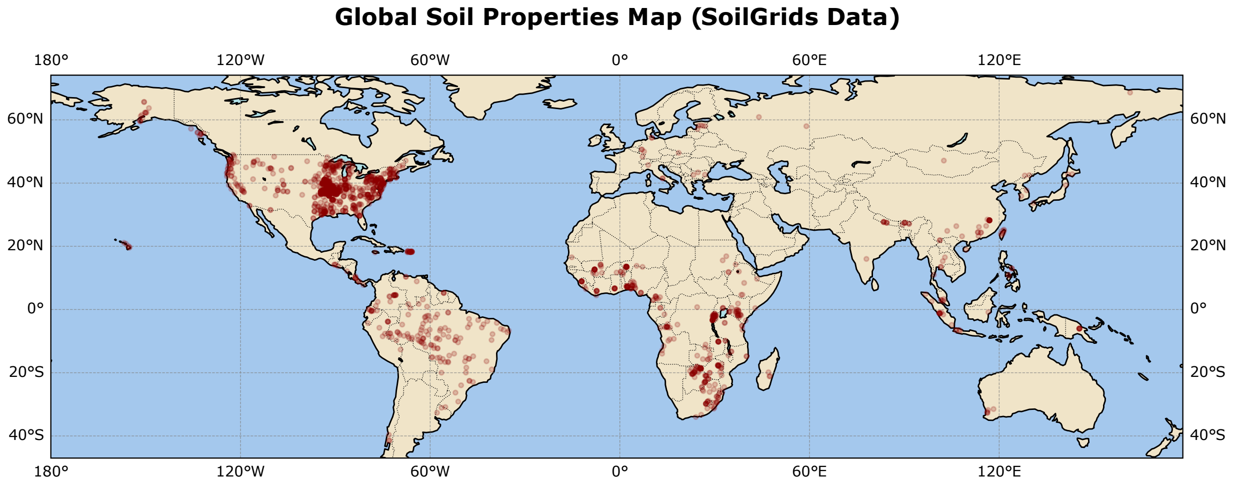

Figure S1. Distribution of the soil sampling coordinates resulting from the SoilGrids dataset.

The KNN imputer identifies the *k* most similar instances (neighbors) to a data point with missing values.[30] It computes the similarity between data points based on the distance in feature space, typically using Euclidean distance. For each missing value, the imputer finds the *k*-nearest data points with known values for that feature and imputes the missing value as the weighted average (or median) of the neighbors' values. The Euclidean distance d(x_i x_j) between two data points x_i, x_j, each represented by a set of features, is calculated as Equation S5:

$$d(x_i, x_j) = \sqrt{\sum_{f=1}^{n} (x_{if} - x_{jf})^2} \quad \text{(S5)}$$

where x_i, x_j are the values of the feature f for the data points x_i, x_j, and n is the total number of features. Once these distances are calculated, the k nearest neighbors are selected. The imputed value for the missing feature $v_{missing}$ is then estimated as Equation S6:

$$v_{missing} = \frac{1}{k}\sum_{i=1}^{k} v_i \qquad \text{(S6)}$$

where v_i represents the values of the feature from the k nearest neighbors. This approach ensures that imputed values reflect the patterns and relationships present in the surrounding data, leveraging the inherent spatial and feature-based correlations in soil properties. By employing KNN imputation, we preserved the structure and variability of the original dataset while minimizing bias that could arise from removing incomplete data [30].

The KNN imputer model allowed us to predict certain soil physicochemical properties that were lacking in the K_d (PFAS) dataset, especially soil pH (≈ 4% of the total data was imputed), CEC (≈ 20% of the total data was imputed), sand, silt, and clay (≈ 8% of the total data was imputed) information. C_{ORG} was available for all accepted entries in Supplementary File 1.

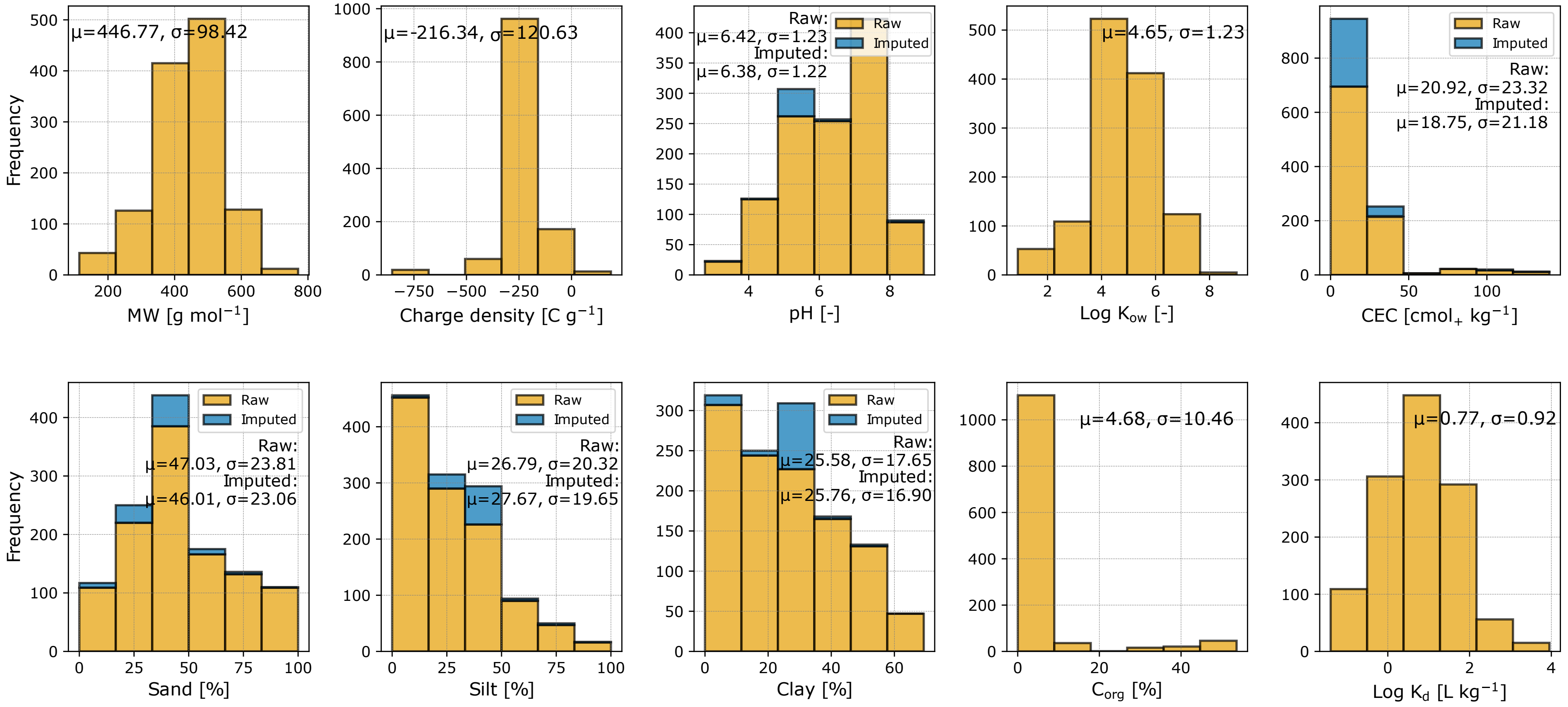

Figure S2. Imputed soil- and PFAS-specific property ranges used for the model construction. The histograms illustrate the distributions of various soil and PFAS properties, comparing raw (orange) and imputed (blue) data. Statistical parameters (i.e., mean (μ) and standard deviation (σ) of the populations) are displayed.

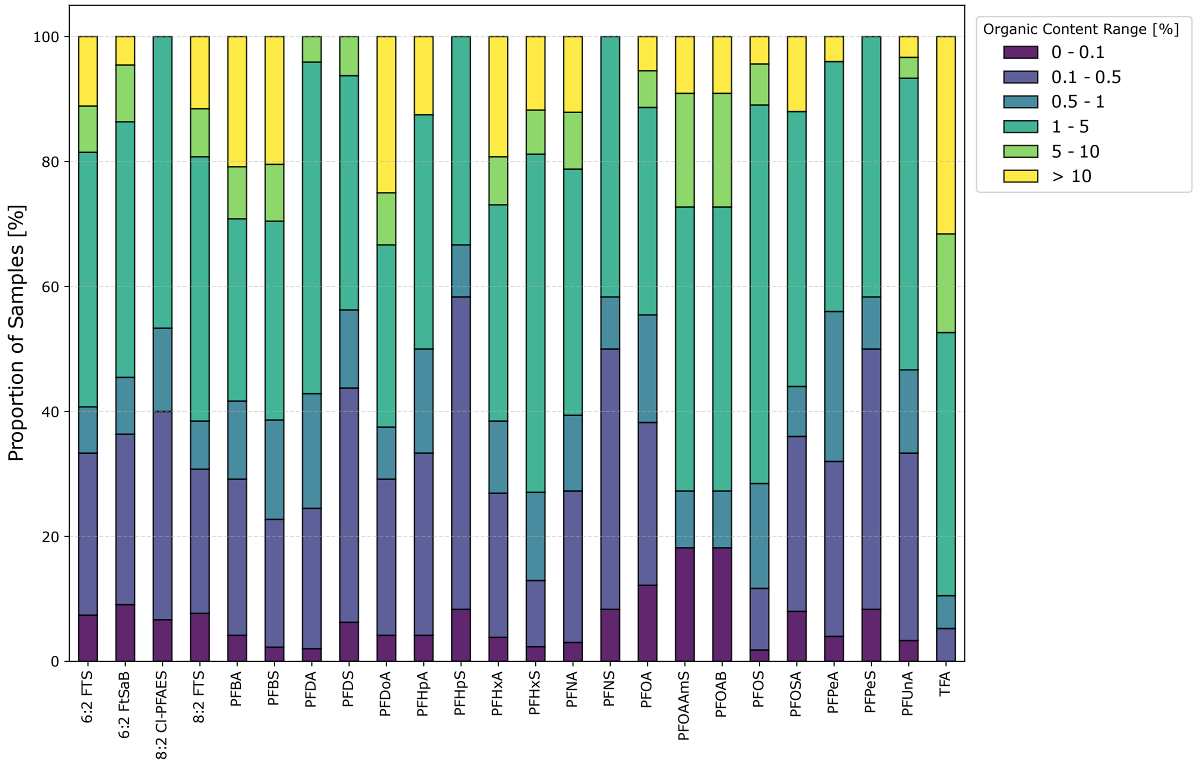

Figure S3. Distribution of PFAS entries for several organic carbon content (C_{ORG}) ranges. The stacked bar plot shows the proportion of soil samples categorized into six C_{ORG} ranges: 0 – 0.1%; 0.1 – 0.5%; 0.5 – 1%; 1 – 5%; 5 – 10%; >10%. The height of each bar represents the proportion of samples within each C_{ORG} range.

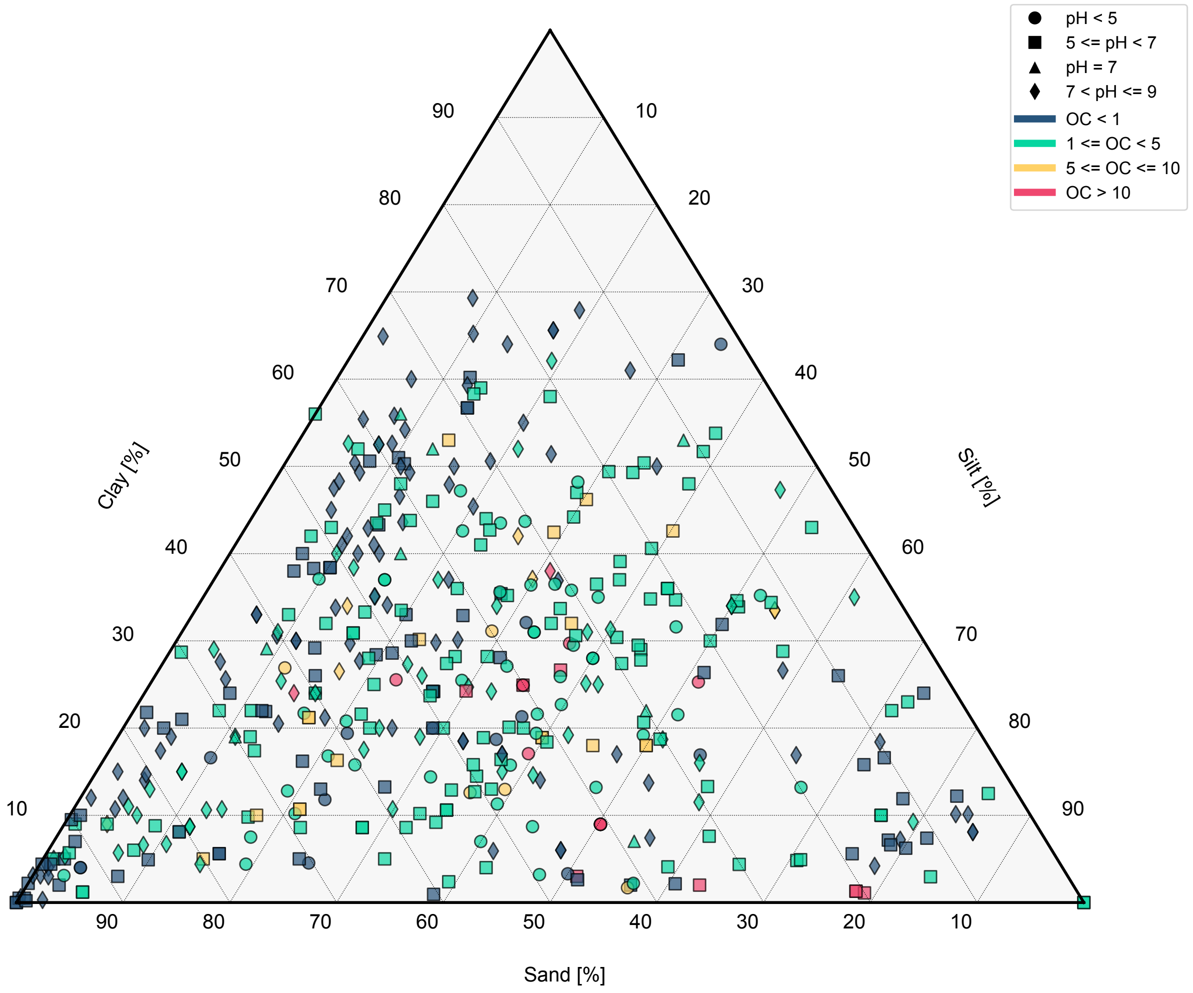

Figure S4. Soil texture visualization illustrating the distribution of soil samples based on relative sand, silt, and clay contents, with overlaid data points representing different pH (marker shape, •: pH < 5; ■: 5 ≤ pH < 7; ▲: pH = 7; ♦: 7 < pH ≤ 9) and C_{ORG} (%, marker color, blue: C_{ORG} < 1%; green: 1 ≤ C_{ORG} < 5%; yellow: 5 ≤ C_{ORG} ≤ 10%; red: C_{ORG} > 10%) levels.

S6: Additional information on data preprocessing

Min-Max Scaling

Min-Max scaling is a normalization technique that adjusts feature values to fall within a predefined range, commonly [0, 1]. Each feature x is transformed using Equation S7:

$$x' = \frac{x - \min(x)}{\max(x) - \min(x)} \tag{S7}$$

where x' represents the scaled value, $\min(x)$ is the minimum value, and $\max(x)$ is the maximum value of the feature. This method preserves the relative distribution of the data while making all features comparable in magnitude.

Random Holdout Split

The holdout split is a method for dividing a dataset into two subsets: one for training the model and another for testing its performance. This method ensures that the model's evaluation reflects its ability to generalize to unseen data. Typically, a split ratio of 80:20 is used, where 80% of the data is used for training the model, and 20% is used for testing the predictive performance of the model.

The splitting process is performed using a random sampling approach, controlled by a pseudo-random number generator. To ensure reproducibility, a fixed random seed is specified, which guarantees that the same split is produced across different runs. Random shuffling of the data is applied prior to splitting to remove any inherent ordering bias, ensuring that both subsets are representative of the overall dataset distribution.

S7: Additional information on predictive performance metrics

Normalized Root Mean Squared Error (NRMSE)

The NRMSE was calculated to evaluate the prediction accuracy by normalizing the Root Mean Squared Error (RMSE) with respect to the range of observed values (Equation S8). This metric provides an understanding of the prediction error relative to the data's scale by allowing comparison of errors across different variables:

$$NRMSE = \frac{\sqrt{\frac{1}{n}\sum_{i=1}^{n}(y_i - \hat{y}_i)^2}}{y_{\max} - y_{\min}} \quad \text{(S8)}$$

where y_i is the measured log K_d value, $\hat{y}_i$ is the predicted log K_d value, y_{max} and y_{min} are the maximum and minimum measured log K_d values, and n is the total number of data entries.

Ratio of Performance to Deviation (RPD)

The RPD is a metric representative of the log K_d predictive performance that compares the standard deviation of the measured data to the RMSE of predictions (Equation S9). This is used to evaluate how well the model log K_d predictions approximate the variation in the measured log K_d data. A higher RPD is indicative of better model performance:

$$RPD = \frac{\sigma_{\text{measured}}}{RMSE} \quad \text{(S9)}$$

where σ measured is the standard deviation of the measured log K_d values, and RMSE is the root mean square error. For environmental analysis, RPD < 1.5 values indicate poor performance, while RPD values ranging from 1.5 to 2.0 indicate acceptable quality. RPD values > 2.0 indicate good quality, especially those with RPD ≥ 3.0, which are considered as analytical quality [16].

K-fold Cross-validated Normalized Root Mean Square Error (CVNRMSE)

CV-NRMSE is a metric used to assess the model's performance across different folds during cross-validation. By normalizing the RMSE based on the data's range, this metric provides an estimate of how well the model generalizes to unseen data. It is calculated similarly to NRMSE, but is specifically applied during the cross-validation process (Equation S10):

$$CV\ NRMSE = \frac{\sqrt{\frac{1}{k}\sum_{j=1}^{k}\left(\frac{1}{n_j}\sum_{i=1}^{n_j}\left(y_i^{(j)} - \hat{y}_i^{(j)}\right)^2\right)}}{y_{\max} - y_{\min}} \qquad \text{(S10)}$$

where k is the number of cross-validation folds, n_j is the number of samples in fold j, and $y_i^{(j)}$ and $\hat{y}_i^{(j)}$ are the measured and predicted log K_d values for fold j. The CVNRMSE helps ensure that the model's error estimates are reliable and not overfitted to a particular training set.

Determination Coefficient (R^2)

This metric evaluates the amount of variance in the measured data explained by the model's predictions based on simple or multiple linear regression. It provides a measure of the model's explanatory power, where a value close to 1 indicates a high proportion of variance explained, and a value close to 0 indicates a low proportion of variance explained (Equation S11):

$$R^2 = 1 - \frac{\sum_{i=1}^{n}(y_i - \hat{y}_i)^2}{\sum_{i=1}^{n}(y_i - \bar{y})^2} \qquad \text{(S11)}$$

where y_i is the measured log K_d value, $\hat{y}_i$ is the predicted log K_d value, $\bar{y}$ is the mean of the measured log K_d values, and n is the total number of data entries.

S8: Statistical assessment of log K_{OC} distributions

A comprehensive statistical analysis was conducted to evaluate differences in log K_{OC} populations across various PFAS species, either by assessing the effect of the number of fluorinated carbons or the PFAS functional group. The statistical workflow was designed to ensure robust and precise comparisons, adhering to assumptions of normality and variance homogeneity wherever applicable. The flowchart of the statistical test selection process prior to assessing the log K_{OC} distributions is depicted in Figure S5.

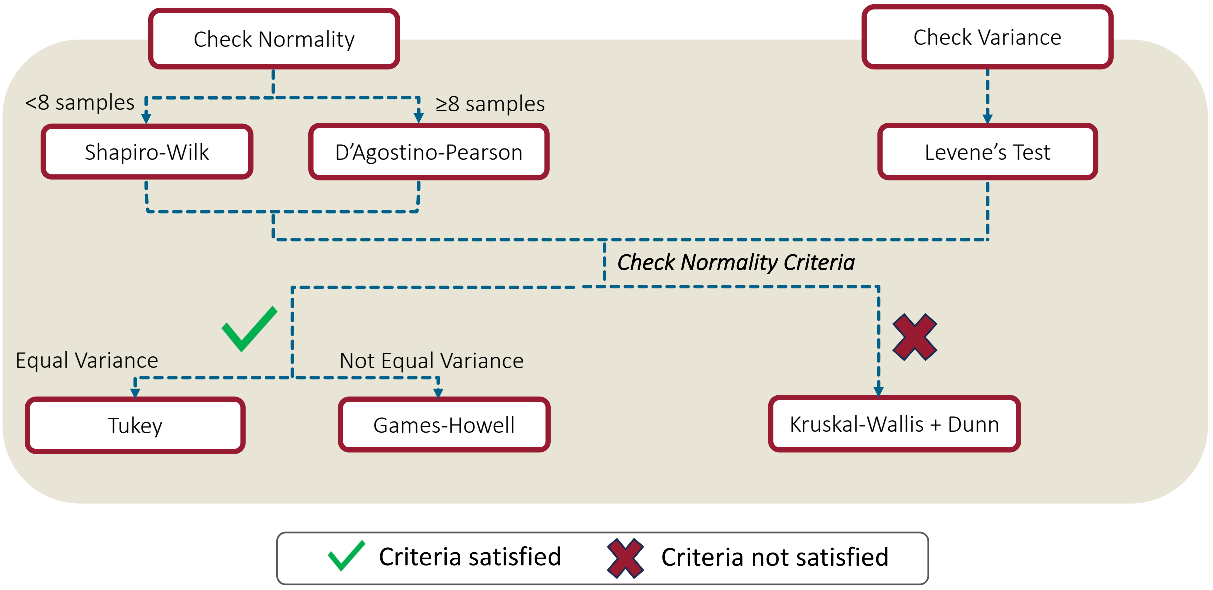

Figure S5. Flowchart of the statistical test selection prior to assessing the log K_{OC} distributions.

Normality Assessment

The distribution of log K_{OC} values within each group was evaluated depending on the number of data entries (n). The Shapiro-Wilk [31] test (conducted at $\alpha = 0.05$) was applied to the log K_{OC} populations with $n < 8$ samples due to its reliability for small datasets. Log K_{OC} populations with $n \geq 8$ were assessed using the D'Agostino-Pearson test [32] (conducted at $\alpha = 0.05$). Groups were deemed normally distributed when $p > 0.05$.

Homogeneity of Variances

To determine whether the variances among log K_{OC} populations were comparable, Levene's test [33] was applied (conducted at $\alpha = 0.05$). A $p > 0.05$ outcome from this test indicated equal variances across groups.

Statistical Testing Framework

Based on the results of both the normality and variance tests, appropriate statistical methods were selected. A one-way analysis of variance (ANOVA) combined with Tukey's honestly significant difference (HSD) [34] was conducted at $\alpha = 0.05$ and applied when all groups were normally distributed and had equal variances. The Welch's ANOVA [35] combined with the Games-Howell test [36] was conducted at $\alpha = 0.05$ and applied when all groups were normally distributed but had unequal variances. The Kruskal-Wallis test [37] combined with Dunn's test [38] was conducted at $\alpha = 0.05$ and applied when any group deviated from normality. In all cases, groups with $p \geq 0.05$ were considered statistically indistinguishable and were assigned the same alphabetical label.

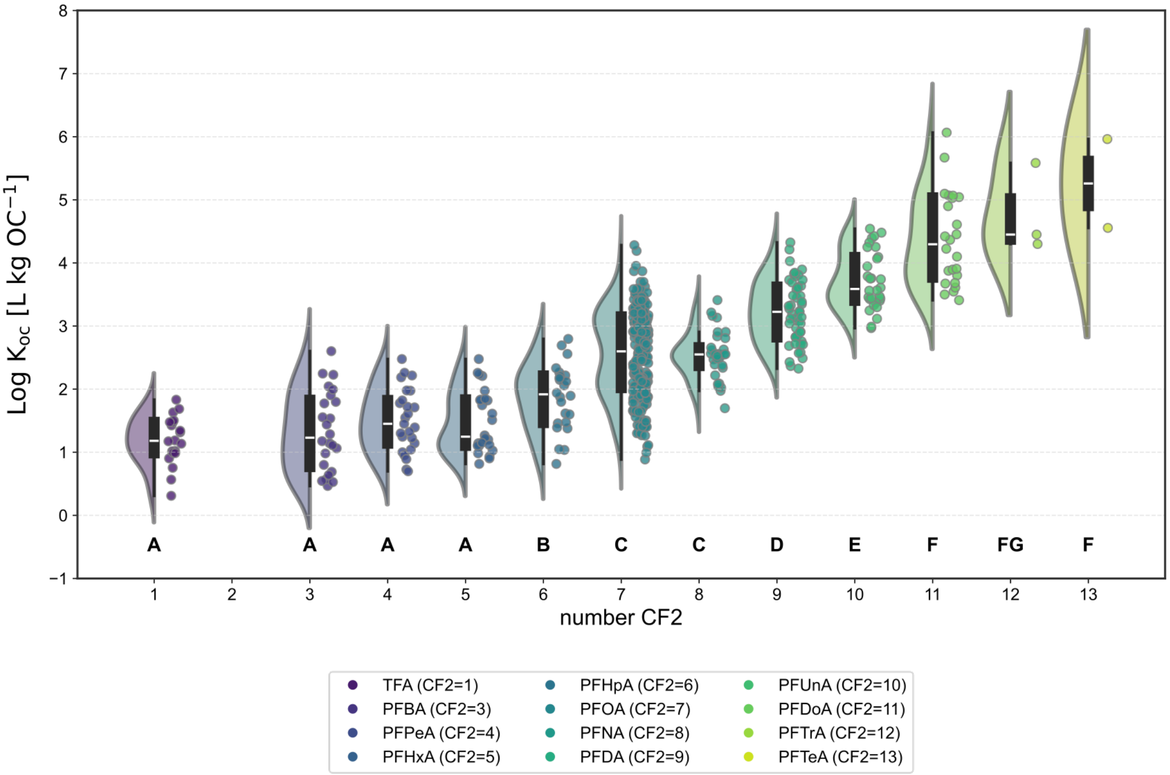

Figure S6. Effect of PFAS chain length on log K_{OC} for the different PFCA species

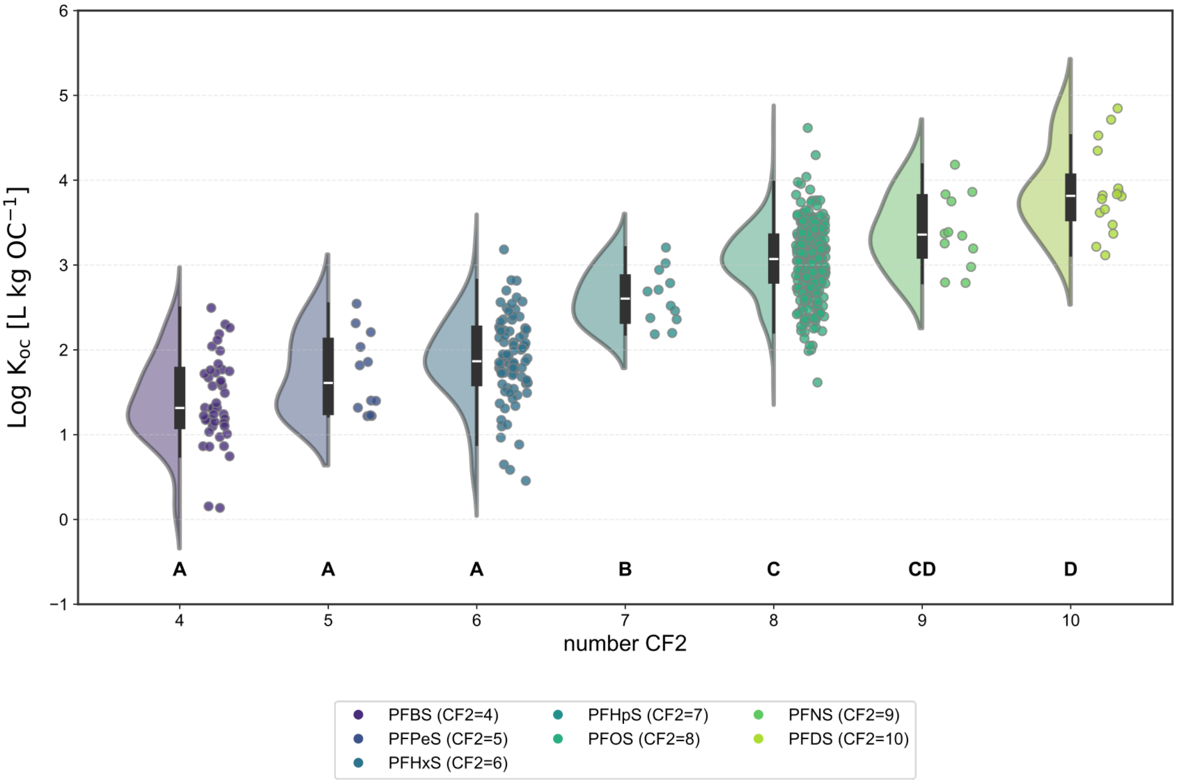

Figure S7. Effect of PFAS chain length on log K_{OC} for the different PFSA species

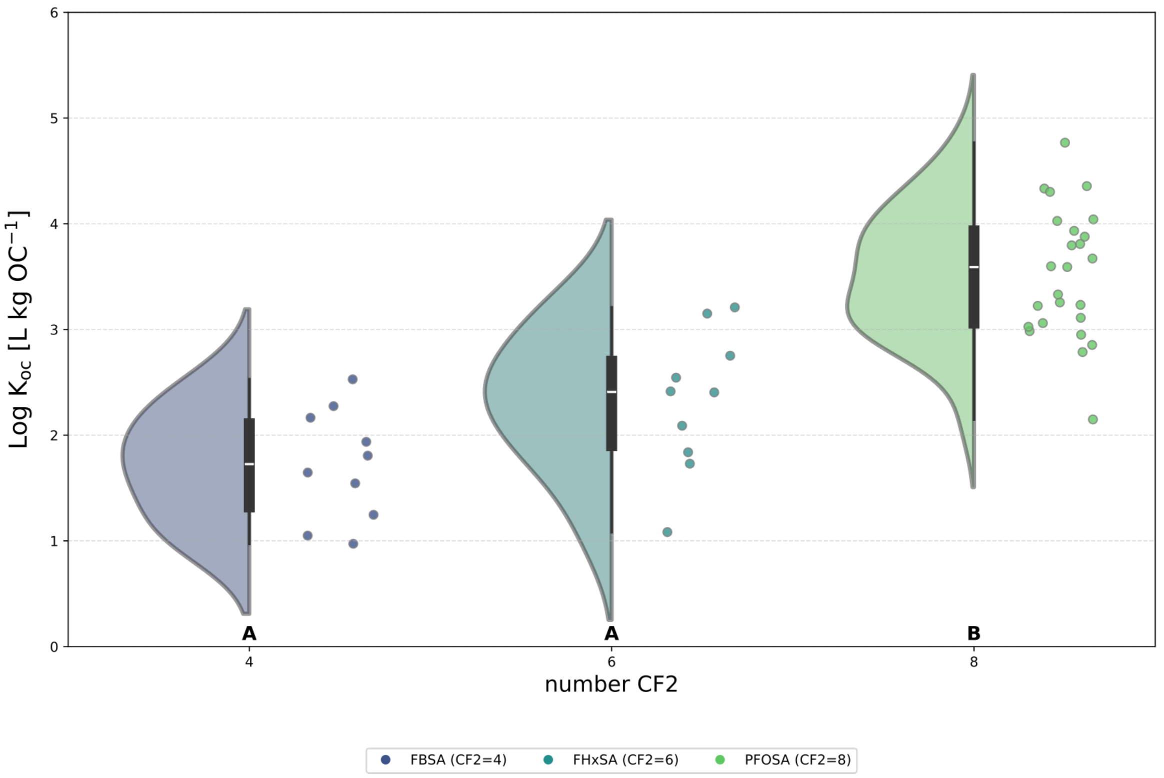

Figure S8. Effect of PFAS chain length on log K_{OC} for the different FOSA species

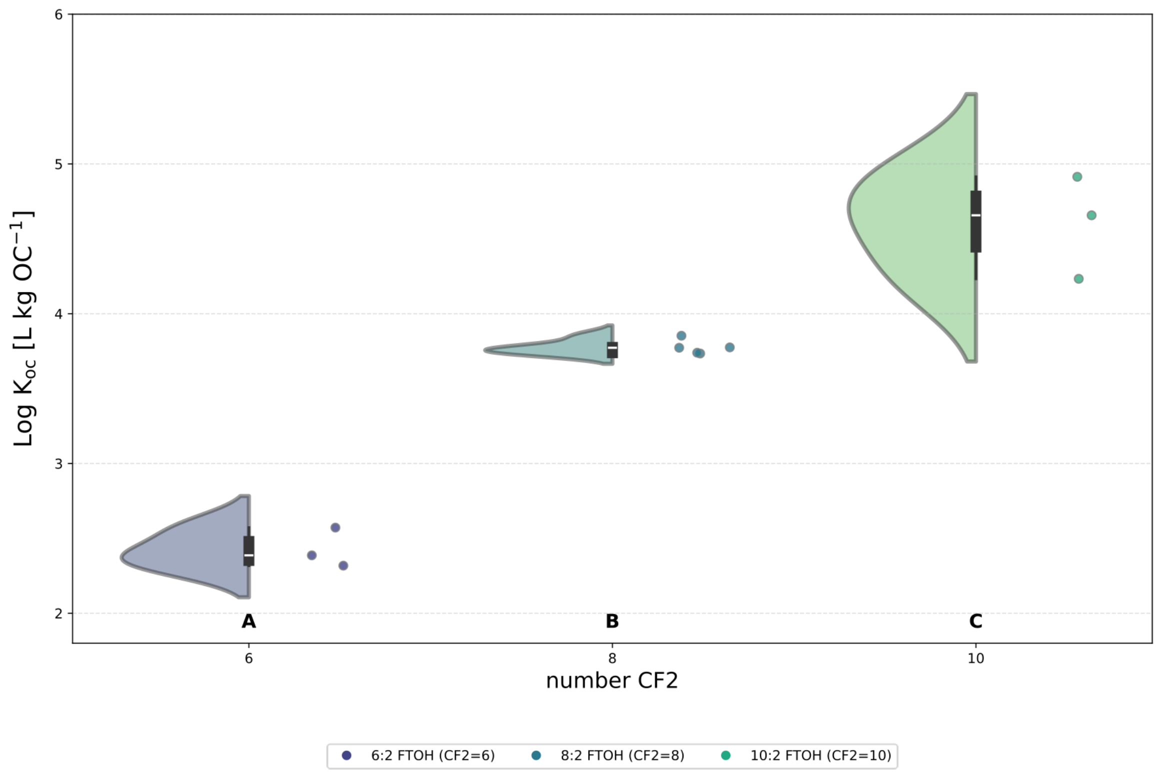

Figure S9. Effect of PFAS chain length on log K_{OC} for the different FTOH species

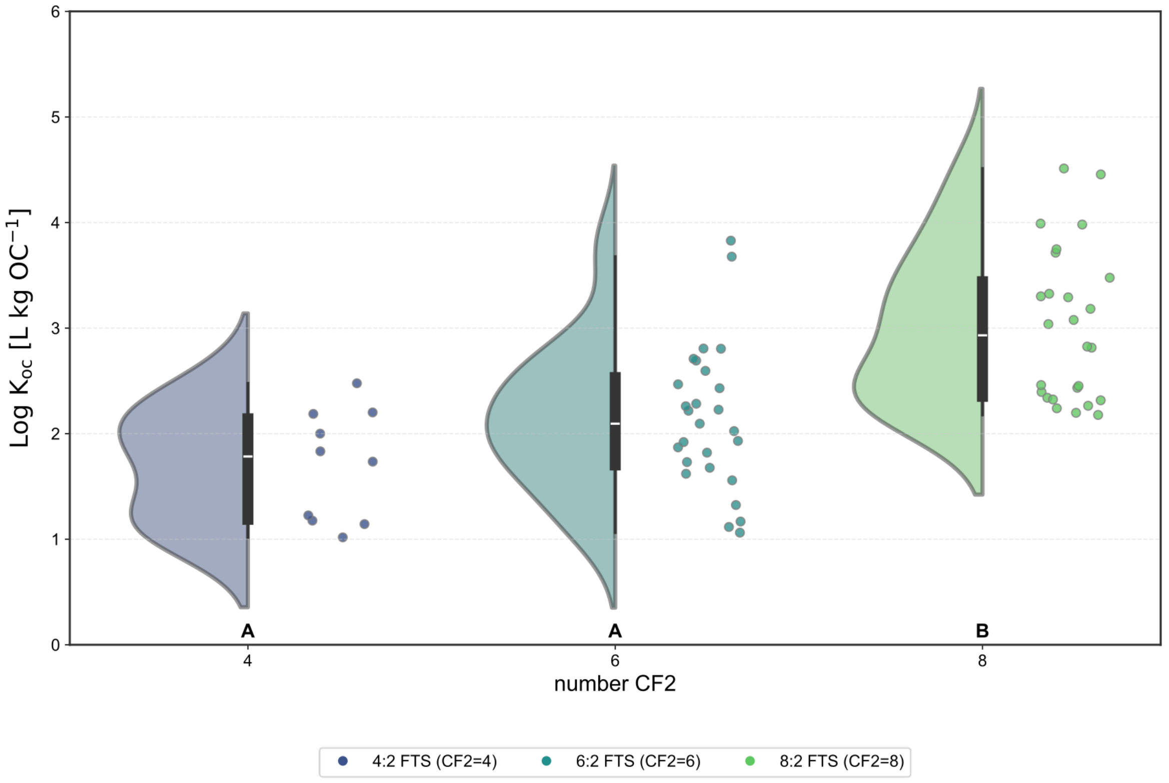

Figure S10. Effect of PFAS chain length on log K_{OC} for the different FTS species

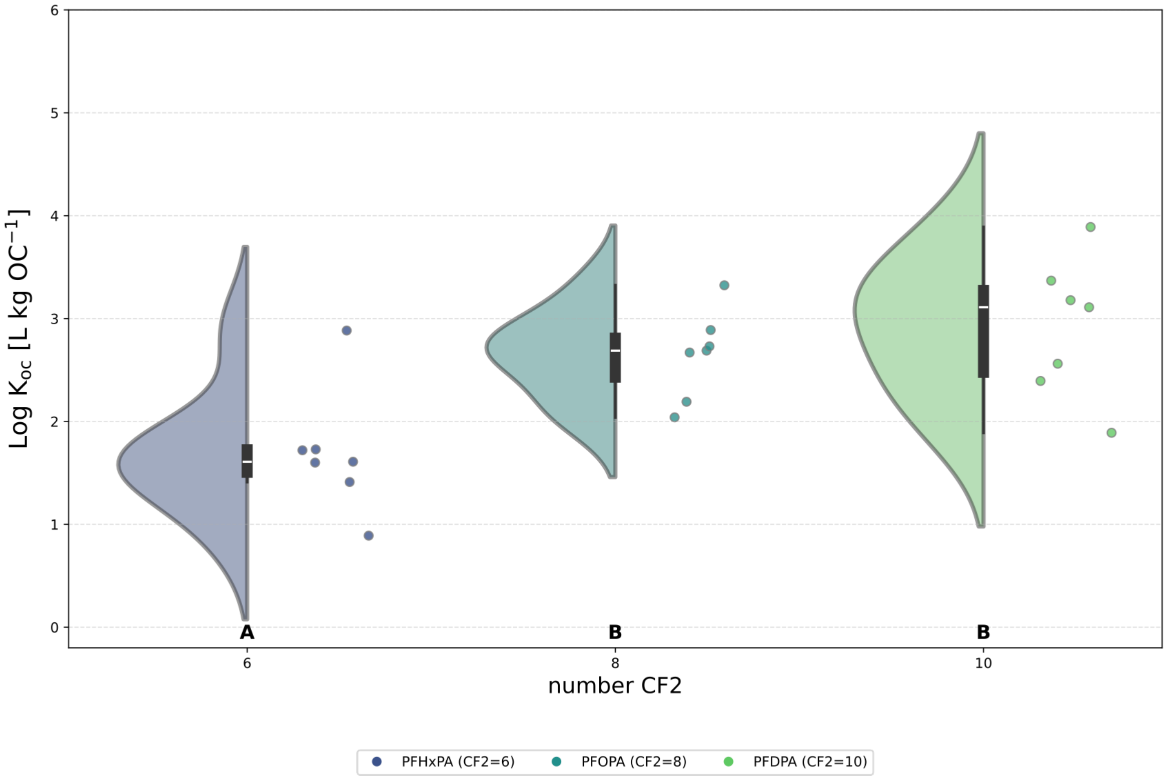

Figure S11. Effect of PFAS chain length on log K_{OC} for the different PFPA species

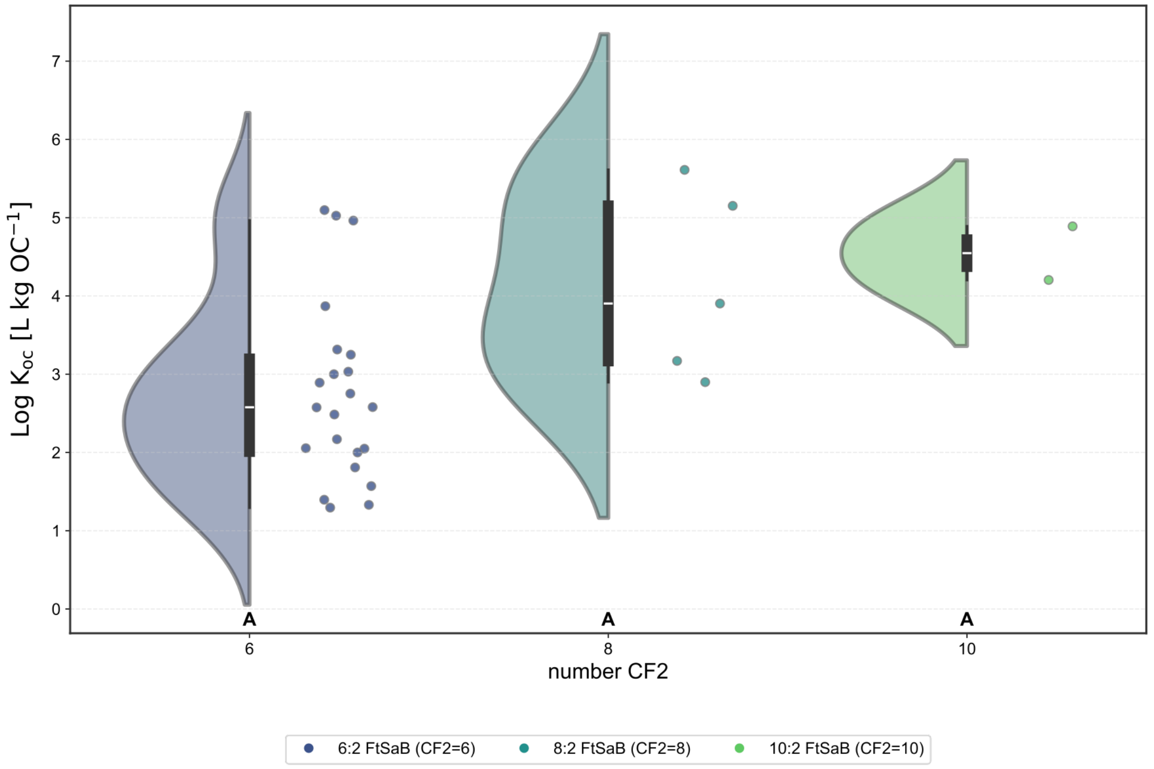

Figure S12. Effect of PFAS chain length on log K_{OC} for the different zwitterionic species

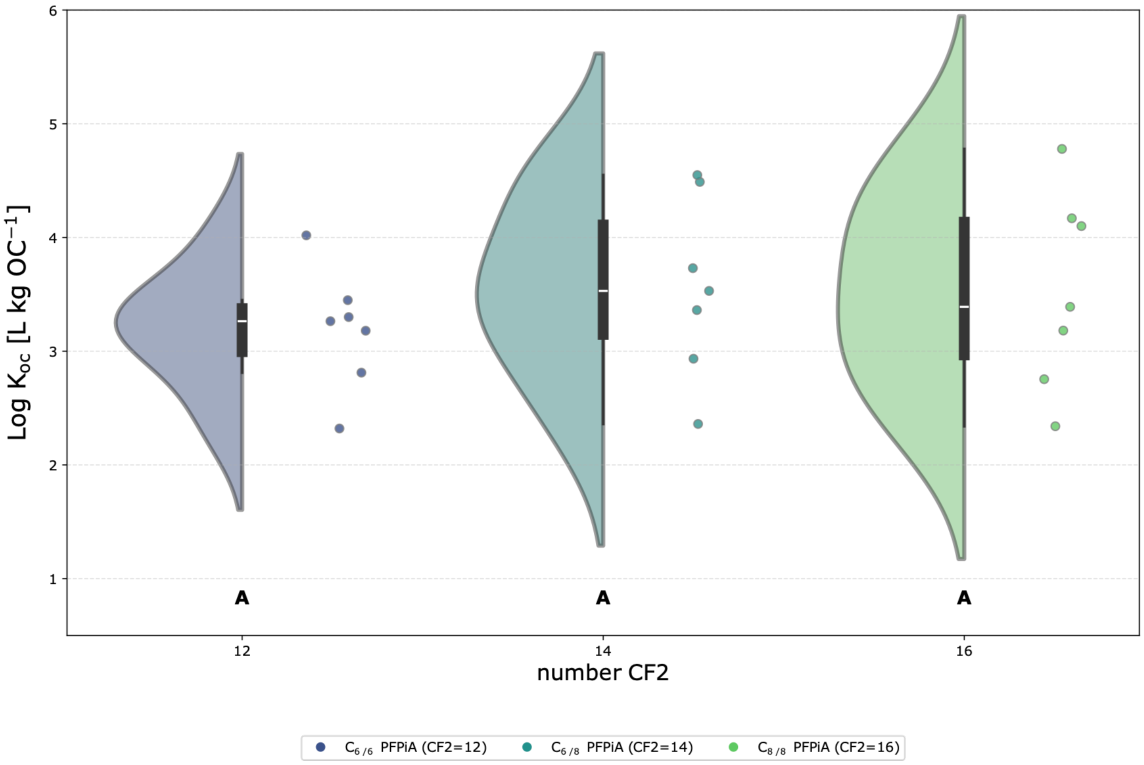

Figure S13. Effect of PFAS chain length on log K_{OC} for the different PFPiA species

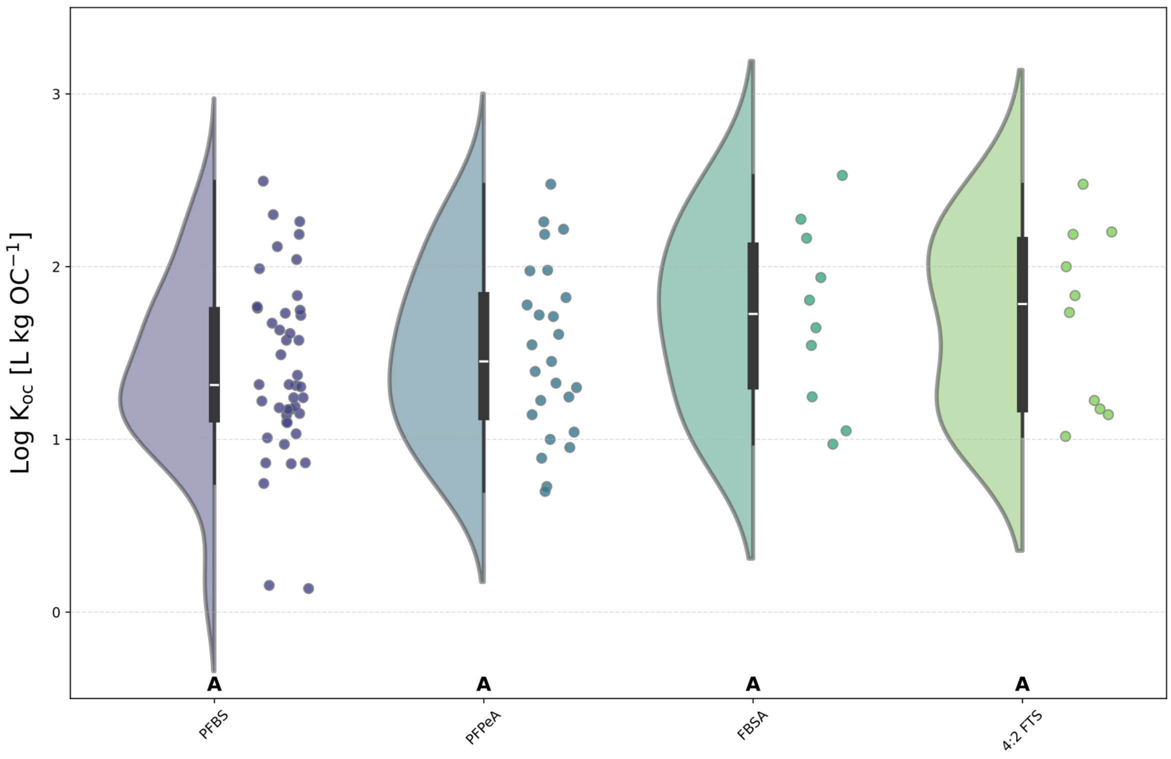

Figure S14. Effect of PFAS functional group for a chain length of 4 fluorinated carbons

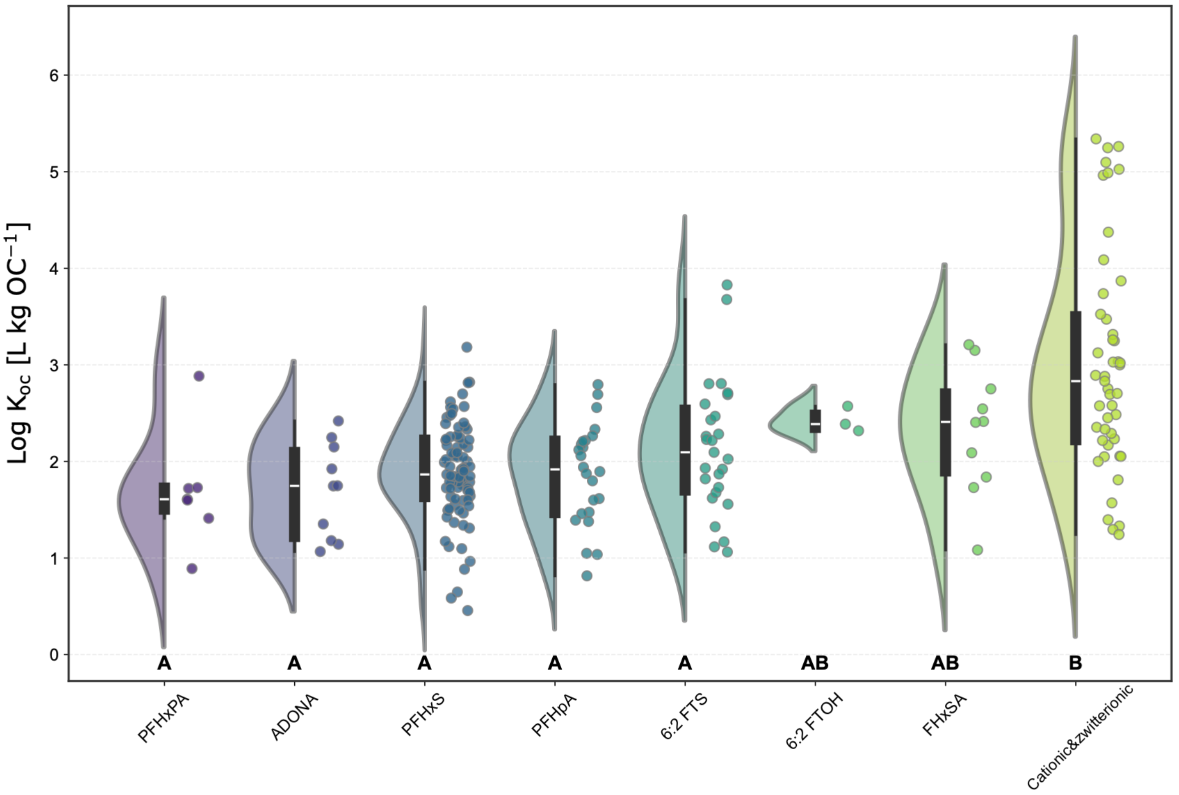

Figure S15. Effect of PFAS functional group for a chain length of 6 fluorinated carbons

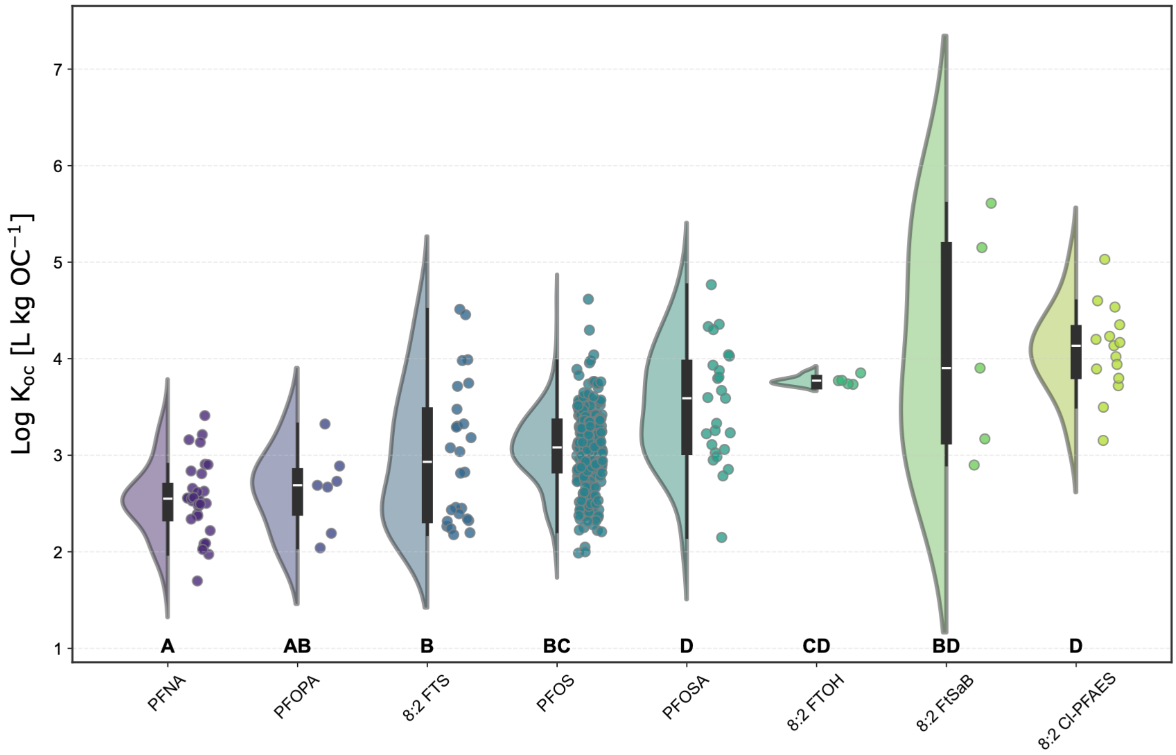

Figure S16. Effect of PFAS functional group for a chain length of 8 fluorinated carbons

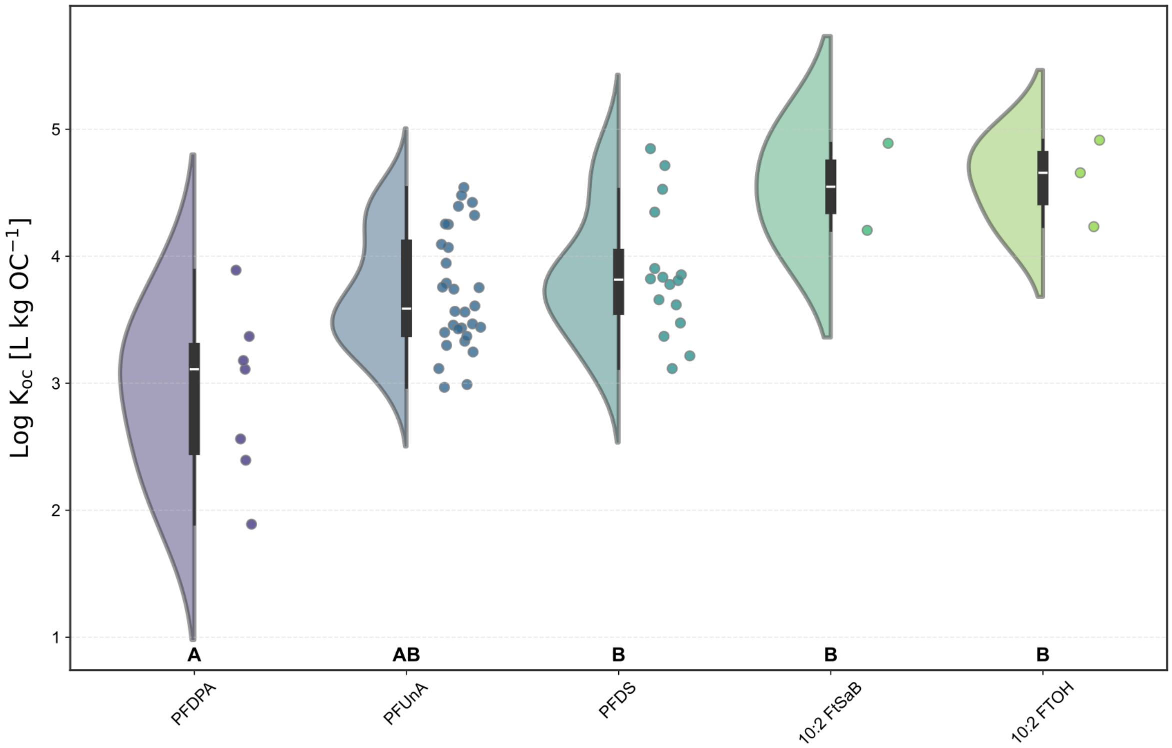

Figure S17. Effect of PFAS functional group for a chain length of 10 fluorinated carbons

S9: Comparison of model performance with other available tools

Our model's predictive metrics were compared against other available K_d (PFAS) predictive tools. EPISuite allows the derivation of K_{OC} values (estimated based on K_{OW} using KOWWIN v1.68) with the only input requirement being the CAS number and SMILES formula [26]. Therefore, it is a suitable first estimate to calculate K_d for any soil/PFAS pair as in Equation S12:

$$K_d = K_{OC} f_{OC} \qquad \text{(S12)}$$

where K_{OC} [L kg OC−1] is the result of EPISuite prediction for the tested PFAS, and f_{OC} is the fraction of soil organic carbon [kg OC kg soil−1].

The thermodynamically-based mechanistic model presented by Higgins and Luthy (2007) was developed to predict the K_d of PFCA and PFSA in five sediments with $f_{OC} \leq 10\%$ via both electrostatic and hydrophobic interactions with the organic matter [39], as in Equation S13:

$$K_d = f_{OC} \frac{F_{access} \bar{V}_W}{\rho_{OC} \bar{V}_i} exp(-1)\, exp\left(\frac{-\Delta G_{hyd,i} - z_i F \Psi_D}{R T}\right) \qquad \text{(S13)}$$

where f_{OC} is the fraction of soil organic carbon [kg OC kg soil^{-1}], ρ_{OC} is the organic matter density [kg OC L OC^{-1}], F_{access} [-] is the fraction of organic matter accessible for the sorbate *i* ($F_{access} \approx 0.34$), $\bar{V}_W$ and $\bar{V}_i$ are the molar volumes of water (0.018 L mol^{-1}) and sorbate *i*, respectively, $\Delta G_{hyd,i}$ [KJ mol^{-1}] is the hydrophobic free energy change in sorption for the sorbate *i*, z_i [-] is the charge of the sorbate, *F* is the Faraday constant [C mol^{-1}], Ψ_D is the organic matter's electrostatic potential [V], *R* is the universal gas constant [J K-1 mol-1], and *T* is temperature [K].

Fabregat-Palau and coworkers [14] developed a model applicable to any soil type to predict K_d values for PFCA and PFSA with a number of fluorinated carbons ranging 3 – 11 based on independent interactions of PFAS with soil C_{ORG} and mineral (*i.e.*, silt and clay fractions) domains, as in Equation S14:

$$K_d = K_{OC}\, f_{OC} + K_{MIN}\, f_{MIN} = 10^{(0.47\ x\ CF2-0.70)}\, f_{OC} + 10^{(0.32\ x\ CF2-1.70)}\, f_{S+C} \qquad \text{(S14)}$$

where K_{MIN} is the mineral-normalized sorption coefficient [L kg reactive mineral fraction^{-1}] and f_{S+C} is the fraction of reactive mineral fraction [kg silt+clay kg soil^{-1}].

Knight and coworkers [16] developed a K_d (PFOA) model after running a multiple linear regression to 100 soils with $f_{OC} \leq 3.5\%$, pH ranging 4.9 – 8.6, and silt+clay contents ranging 5 – 88%. These property ranges, therefore, set the model applicability range, which according to our data assessment had the following form (Equation S15):

$$K_{d,PFOA} = -0.52\, pH + 3.2\, C_{ORG} + 0.04\, (Silt + Clay) + 5.75 \qquad \text{(S15)}$$

where pH [-], C_{ORG} [%], and silt + clay [%] are the soil physicochemical properties.

Umeh and coworkers [40] developed a K_d (PFOS) model based on an artificial neural network trained on sorption data from 114 soils of contrasting properties but limited to soils with $f_{OC} \leq 10\%$. While their model did not contain an explicit equation, they developed an online platform to predict K_d (PFOS).

Similarly, Xie and coworkers [41] recently developed a K_d (PFAS) prediction model based on the application of random forest machine learning approaches to a K_d (PFAS) dataset covering a wide range of PFAS (including PFCA, PFSA, FTS, FOSA, and other novel PFAS) and soil properties. Unfortunately, their model did not contain an explicit equation, nor provided a platform to test the model.

Table S3 summarizes some of the reported metrics in each study, where we additionally include the prediction of our model and additional prediction metrics originating from EPISuite (Equation S12).

Table S3. Reported metrics evaluating the performance for different K_d (PFAS) prediction tools

Model	Type of validation	Number of validation data	RPD	Explained Variance
EPIsuit (this study)	Independent test set (20% of the total)	231	1.33	44
Higgins and Luthy	Independent test set (100% of the total)	≈ 80	N.A.	93
Fabregat-Palau et al.,	Independent test set (33% of the total)	121	1.88	76
Knight et al.,	95% of their own training set	95	1.83	62
Umeh et al.,	Cross validation of their own dataset	114	N.A.	84
Xie et al.,	Cross validation of their own dataset	2,328	N.A.	93
PSSM * (this study)	Independent test set (20% of the total)	231	3.13	89

Note: N.A. Not Available; *: PFAS Sorption Stacking Model

Based on the metrics listed in Table S3, our model outperformed the prediction quality of other currently available tools, as exemplified by the highest RPD value across studies and with a high explained variability. Furthermore, the good prediction of K_d (PFAS) data originating from various studies and obtained under various batch conditions (*i.e.*, differing nature of the contact solution) over those studies assessing sorption for specific PFAS under certain constant batch conditions (*e.g.*, Knight et al., 2019 and Umeh et al., 2021) highlights the broader applicability of our model.

Figure S18 exemplifies the prediction of our test set for three of the tools listed in Table S3: EPISuite, Fabregat-Palau et al., 2021 [14], and our developed PSSM. EPISuite and PSSM predictions were examined across the overall test set, while predictions from Fabregat-Palau et al., 2021 [14], were only applied to the test set for those PFCA and PFSA with fluorinated carbon numbers ranging from 3 to 11.

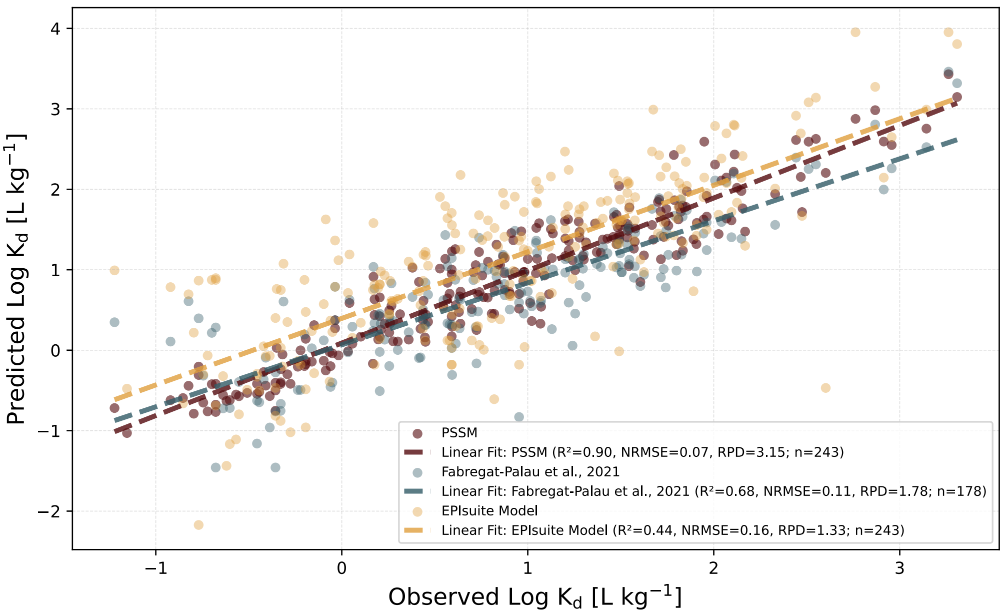

Figure S18. Assessment of different models (i.e., EPISuite, Fabregat-Palau et al., 2021 [14], and PSSM) based on our test set.

As displayed in Figure S18, the prediction accuracy originating from EPISuite was the lowest (*i.e.*, 44% of the data variability explained, RPD = 1.3, n = 243, $p < 0.001$), likely due to K_d (PFAS) output relying only on K_{OC} and f_{OC} values. Despite being useful for an early estimation, EPISuite output does not consider the sorption in reactive mineral fractions, which may be relevant for soils with low C_{ORG} and especially for compounds such as short-chain PFCA and cationic and zwitterionic PFAS [14,20]. Fabregat-Palau et al., 2021 addressed this issue by including these reactive mineral (*i.e.*, silt and clay) fractions, thus achieving a better prediction performance (*i.e.*, 68% of the data variability explained, RPD = 1.7, n = 178, $p < 0.001$) for the PFCA and PFSA species with fluorinated carbon numbers ranging from 3 to 11 [14]. Nonetheless, the developed model, which was built upon linear relationships between K_{OC} and K_{MIN} with the number of fluorinated carbons of the PFAS, did not include the effect of other properties (*e.g.*, pH) in sorption or potential non-linear relationships among variables. The PSSM model addresses this by upgrading the pool of PFAS and including other soil properties beyond C_{ORG} and reactive mineral fractions, which are modeled through non-linear approaches typical in ML, allowing the prediction performance of the overall dataset to rise (*i.e.*, 90% of the data variability explained, RPD = 3.2, n = 243, $p < 0.001$).

S10: Additional geospatial K_d (PFAS) maps

The PSSM model developed in this study was applied to each sampling location of the topsoil LUCAS 2009 repository data for European soils (European Soil Data Centre (ESDAC), https://esdac.jrc.ec.europa.eu/content/lucas-2009-topsoil-data) that had available information for all the model input parameters required (*i.e.*, pH, C_{ORG}, CEC, sand, silt, and clay contents). The total number of soil data points was 21,904. The soil characteristics of the dataset were preliminarily screened (Figure S19). C_{ORG} was generally low (≤ 5%) across southern Europe but increased (≥ 20%) in some regions of northern Europe and the Scandinavian Peninsula. CEC showed a similar pattern to C_{ORG}, with values generally spanning 10–40 $cmol_+ \ kg^{-1}$. Soil pH across central and southern Europe showed slightly alkaline conditions (*i.e.*, pH ≈ 7 – 9), but had acidic conditions (*i.e.*, pH ≈ 4 – 6) in northern Europe, Portugal, and the Scandinavian Peninsula. Regarding soil textural information, central European soils displayed higher silt contents (*i.e.*, ≈ 40 – 80%). Clay contents were generally low (*i.e.*, < 40%) but increased (*i.e.*, ≈ 50–80%) in Southeast Europe. Sand contents generally ranged 10 – 40% in central Europe but increased to contents > 60% in countries like Denmark, northern Germany, and Poland.

The developed PSSM is able to produce geospatial sorption information for PFAS. As an example, we display the outcomes for four PFAS compounds (i.e., TFA (Figure S20), PFOA (Figure S21), PFOS (Figure S22), and PFOSB (Figure S23)), although the end-user is able to access the geospatial K_d information for all 47 PFAS included in the model by using *PFASorptionML* at https://hydrogeochem.geo.uni-tuebingen.de.

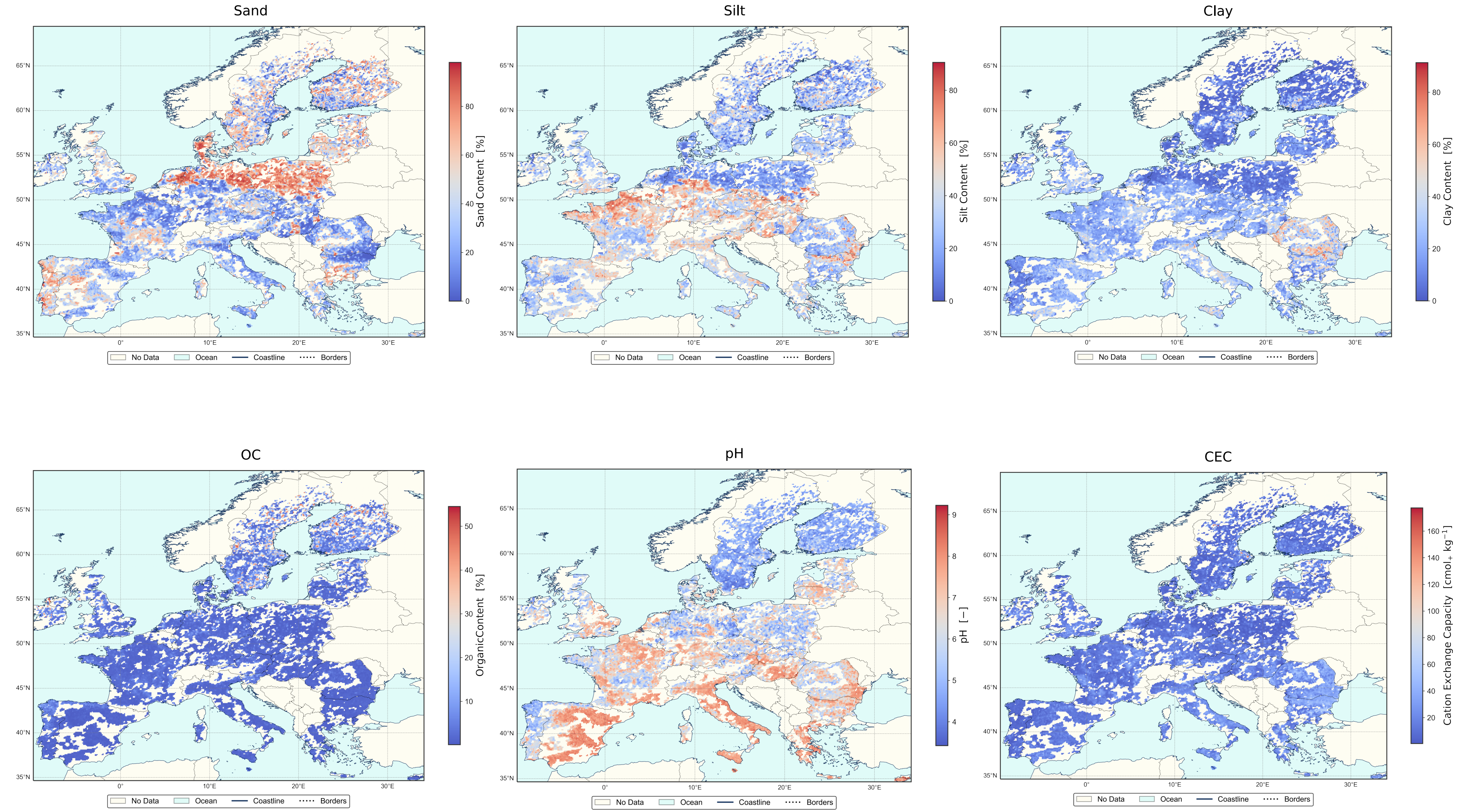

Figure S19. Geospatial characteristics of the European soils

As observed in Figure S20, the K_d values for TFA were low (≈ 0.3 L kg^{-1}) across Europe, indicating very low retention in soil and high mobility to the groundwater table, although higher values (≈ 10 L kg^{-1}) are observed in some regions of the Scandinavian Peninsula, likely as a result of higher C_{ORG} and acidic soil characteristics. TFA is considered a very persistent and very mobile (vPvM) substance that has been increasing in concentration within diverse environmental media including rain and drinking water, with concentrations one order of magnitude higher than those of other PFAS [42]. Our geospatial K_d (TFA) distribution demonstrates the vulnerability of European groundwater towards TFA leaching resulting from rain and application in topsoil.

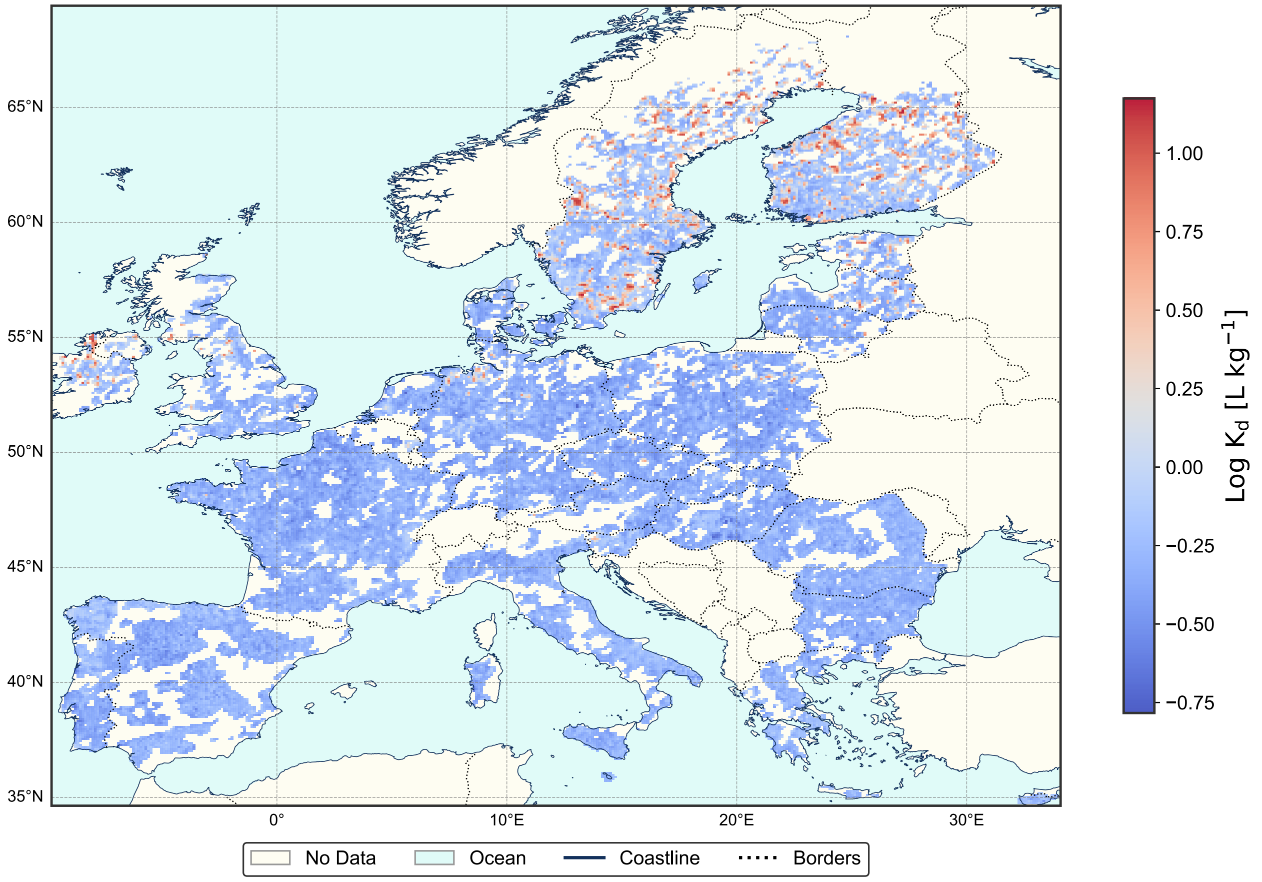

Figure S20. Predicted log K_d values for TFA across Europe using the PSSM model based on soil properties from the LUCAS 2009 repository.

Regarding PFOA, K_d values spanning ≈ 3 – 30 L kg^{-1} may be anticipated across European soils according to its properties (Figure S21). These values suggest a low to moderate sorption to soil particles and, therefore, differing mobility in seepage water. Of relevance are some regions in northern France, Belgium, Denmark, the Netherlands, and north-west Germany, which display relatively low K_d (PFOA) (≈ 3 L kg^{-1}), and therefore, lower retardation to the groundwater table. Higher PFAS concentrations are expected in topsoil in these regions due to the presence of multiple PFAS hotspots [43].

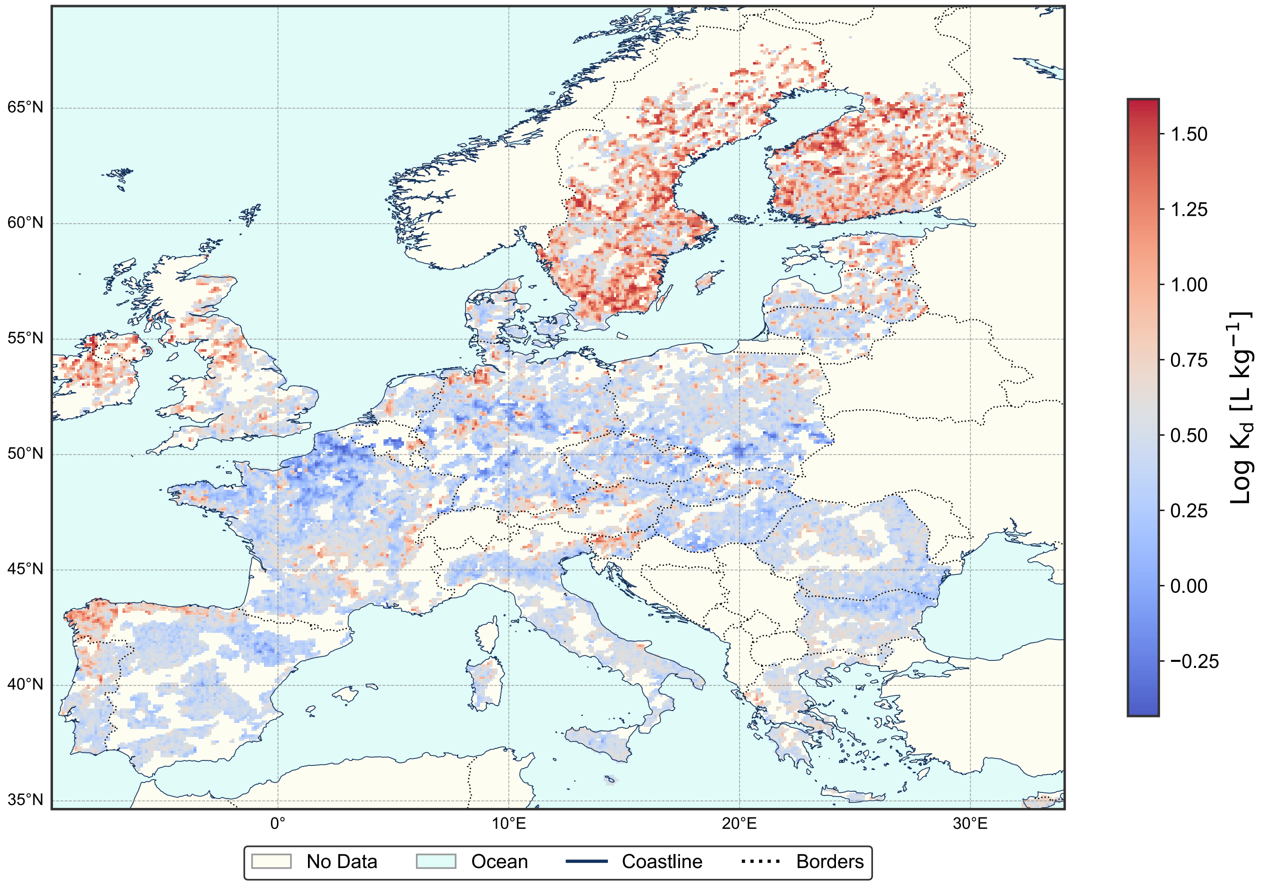

Figure S21. Predicted log K_d values for PFOA across Europe using the PSSM model based on soil properties from the LUCAS 2009 repository.

Regarding PFOS, K_d values ranging from ≈ 10 to ≈ 200 L kg^{-1} are anticipated across European soils (Figure S22), indicating higher retardation to the groundwater table than that expected for PFOA due to stronger sorption to soil particles. Geospatial K_d (PFOS) predictions agree with those observed for PFOA, highlighting a potential threat to groundwater from northern France, Belgium, Denmark, Netherlands, and northwest Germany, where higher PFAS concentrations are expected in topsoil due to the presence of multiple PFAS hotspots [43].

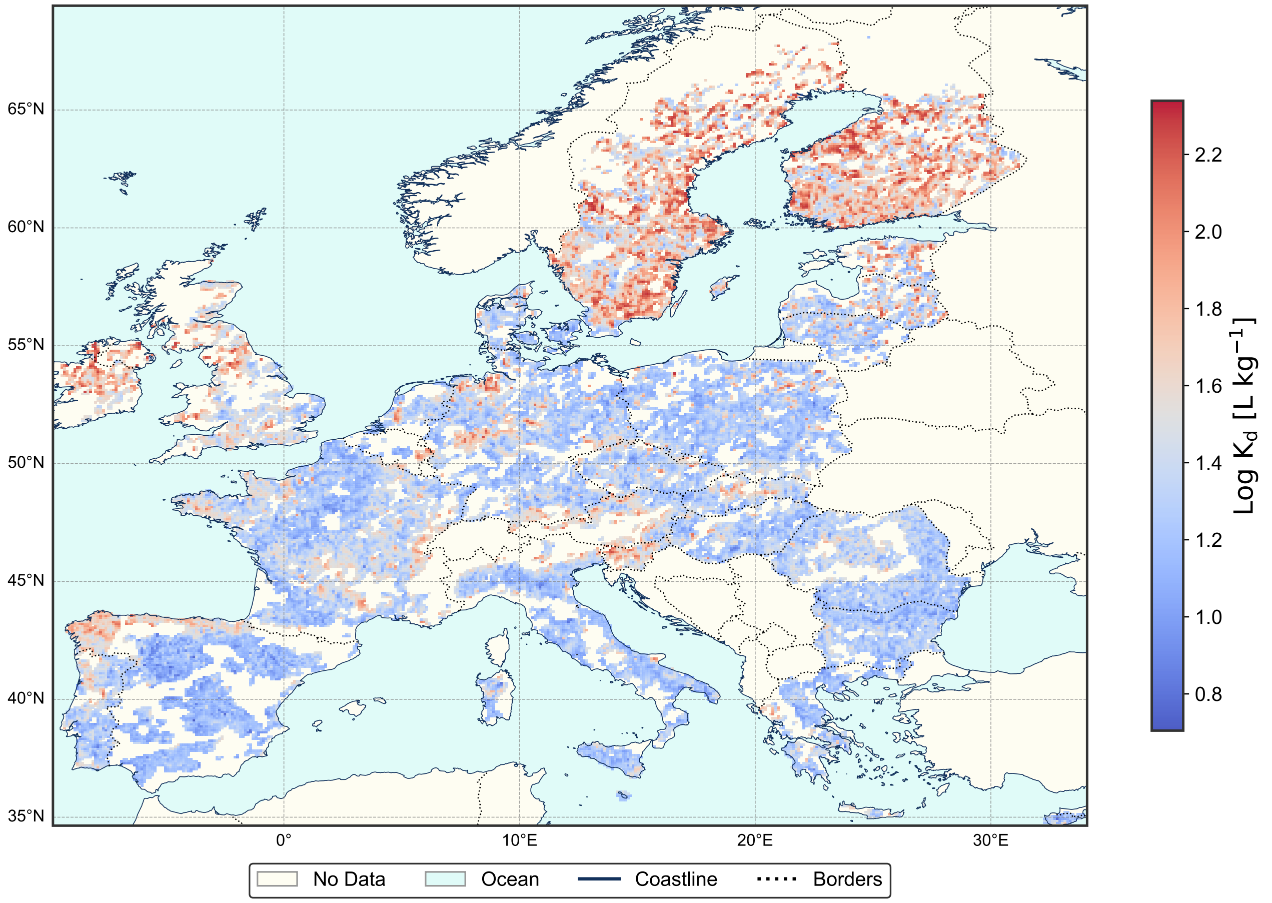

Figure S22. Predicted log K_d values for PFOS across Europe using the PSSM model based on soil properties from the LUCAS 2009 repository.

Regarding PFOSB, selected here as a representative Betaine compound, predicted K_d values across European soils span ≈ 2 to ≈ 200 L kg^{-1} (Figure S23). Sorption geospatial distribution differs from that observed for TFA, PFOA, and PFOS, with higher values observed in Central Europe and the Scandinavian Peninsula. PFOSB, as well as other Betaine-like PFAS, composes the majority of the PFAS burden at AFFF-impacted sites [44]. The higher sorption of PFOSB in locations of Central Europe, which generally have soils with slight alkalinity and relatively higher abundances of silt and clay fractions (see Figure S19), may result from the electrostatic interaction between the cationic group of PFOSB species (see Section S2) and the negatively charged clay surfaces [2]. On the other hand, the higher sorption observed in the Scandinavian Peninsula may result from the higher amount of C_{ORG} in the soil, with an implicit higher CEC and therefore a higher number of sorption sites able to interact with PFOSB through cation exchange mechanisms [2,20].

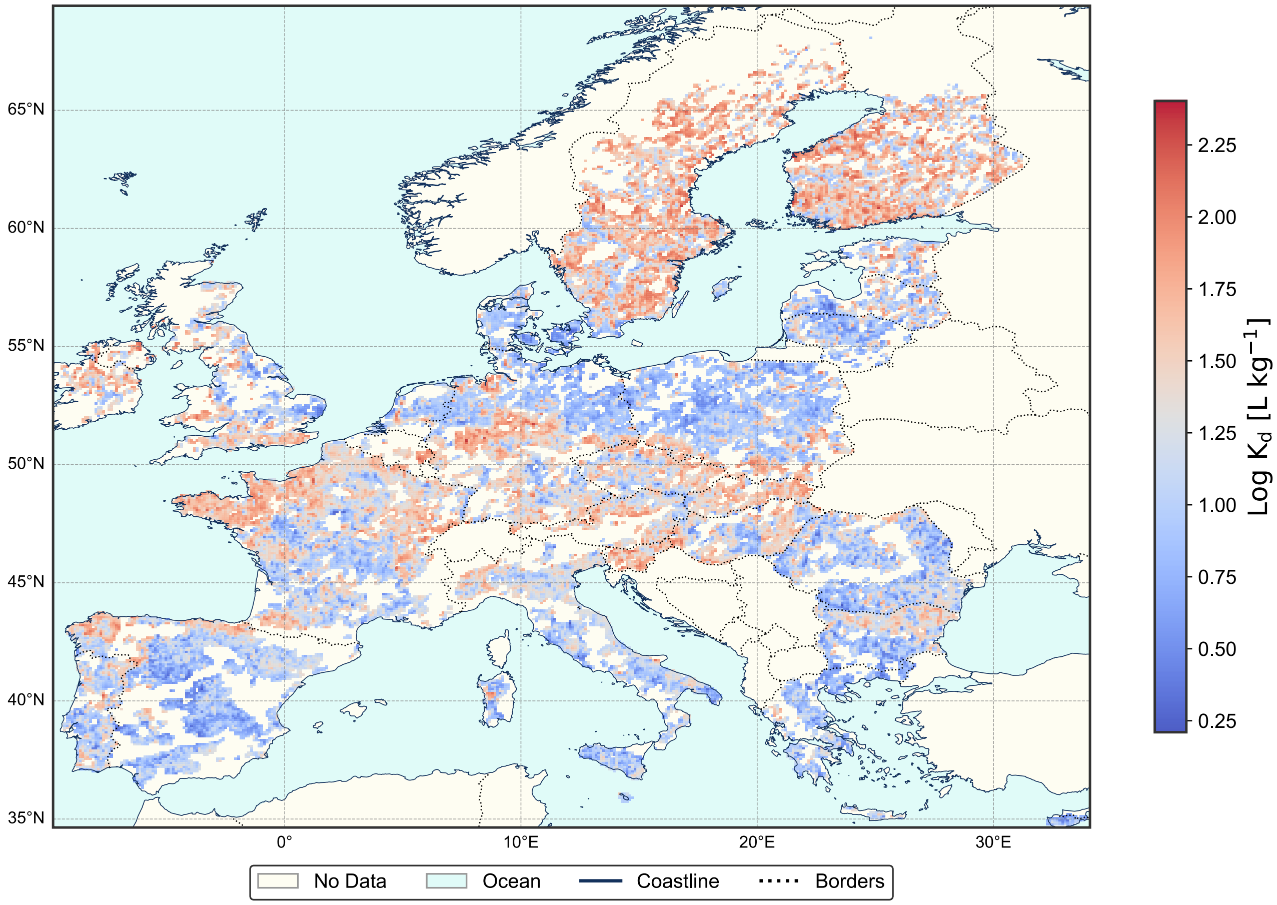

Figure S23. Predicted log K_d values for PFOSB across Europe using the PSSM model based on soil properties from the LUCAS 2009 repository.

S11: References

(1) Mejia-Avendaño, S.; Zhi, Y.; Yan, B.; Liu, J. Sorption of Polyfluoroalkyl Surfactants on Surface Soils: Effect of Molecular Structures, Soil Properties, and Solution Chemistry. *Environ. Sci. Technol.* **2020**, *54* (3), 1513–1521. https://doi.org/10.1021/acs.est.9b04989.

(2) Barzen-Hanson, K. A.; Davis, S. E.; Kleber, M.; Field, J. A. Sorption of Fluorotelomer Sulfonates, Fluorotelomer Sulfonamido Betaines, and a Fluorotelomer Sulfonamido Amine in National Foam Aqueous Film-Forming Foam to Soil. *Environ. Sci. Technol.* **2017**, *51* (21), 12394–12404. https://doi.org/10.1021/acs.est.7b03452.

(3) Gomis, M. I.; Wang, Z.; Scheringer, M.; Cousins, I. T. A Modeling Assessment of the Physicochemical Properties and Environmental Fate of Emerging and Novel Per- and Polyfluoroalkyl Substances. *Sci. Total Environ.* **2015**, *505*, 981–991. https://doi.org/10.1016/j.scitotenv.2014.10.062.

(4) Steinle-Darling, E.; Reinhard, M. Nanofiltration for Trace Organic Contaminant Removal: Structure, Solution, and Membrane Fouling Effects on the Rejection of Perfluorochemicals. *Environ. Sci. Technol.* **2008**, *42* (14), 5292–5297. https://doi.org/10.1021/es703207s.

(5) Xiao, F. Emerging Poly- and Perfluoroalkyl Substances in the Aquatic Environment: A Review of Current Literature. *Water Res.* **2017**, *124*, 482–495. https://doi.org/10.1016/j.watres.2017.07.024.

(6) Baggioli, A.; Sansotera, M.; Navarrini, W. Thermodynamics of Aqueous Perfluorooctanoic Acid (PFOA) and 4,8-Dioxa-3H-Perfluorononanoic Acid (DONA) from DFT Calculations: Insights into Degradation Initiation. *Chemosphere* **2018**, *193*, 1063–1070. https://doi.org/10.1016/j.chemosphere.2017.11.115.

(7) Rayne, S.; Forest, K. Comment on "Indirect Photolysis of Perfluorochemicals: Hydroxyl Radical-Initiated Oxidation of *N*-Ethyl Perfluorooctane Sulfonamido Acetate (*N*-EtFOSAA) and Other Perfluoroalkanesulfonamides." *Environ. Sci. Technol.* **2009**, *43* (20), 7995–7996. https://doi.org/10.1021/es9022464.

(8) Esparza, X.; Moyano, E.; De Boer, J.; Galceran, M. T.; Van Leeuwen, S. P. J. Analysis of Perfluorinated Phosponic Acids and Perfluorooctane Sulfonic Acid in Water, Sludge and Sediment by LC–MS/MS. *Talanta* **2011**, *86*, 329–336. https://doi.org/10.1016/j.talanta.2011.09.024.

(9) Emeléus, H. J.; Smith, J. D. 67. The Heptafluoropropyliodophosphines and Their Derivatives. *J. Chem. Soc.* **1959**, *0* (0), 375–381. https://doi.org/10.1039/JR9590000375.

(10) Guo, B.; Zeng, J.; Brusseau, M. L. A Mathematical Model for the Release, Transport, and Retention of Per- and Polyfluoroalkyl Substances (PFAS) in the Vadose Zone. *Water Resour. Res.* **2020**, *56* (2), e2019WR026667. https://doi.org/10.1029/2019WR026667.

(11) Nguyen, T. M. H.; Bräunig, J.; Thompson, K.; Thompson, J.; Kabiri, S.; Navarro, D. A.; Kookana, R. S.; Grimison, C.; Barnes, C. M.; Higgins, C. P.; McLaughlin, M. J.; Mueller, J. F. Influences of Chemical Properties, Soil Properties, and Solution pH on Soil–Water Partitioning Coefficients of Per- and Polyfluoroalkyl Substances (PFASs). *Environ. Sci. Technol.* **2020**, *54* (24), 15883–15892. https://doi.org/10.1021/acs.est.0c05705.

(12) Richey, D. G.; Driscoll, C. T.; Likens, G. E. Soil Retention of Trifluoroacetate. *Environ. Sci. Technol.* **1997**, *31* (6), 1723–1727. https://doi.org/10.1021/es960649x.

(13) Campos Pereira, H.; Ullberg, M.; Kleja, D. B.; Gustafsson, J. P.; Ahrens, L. Sorption of Perfluoroalkyl Substances (PFASs) to an Organic Soil Horizon – Effect of Cation Composition and pH. *Chemosphere* **2018**, *207*, 183–191. https://doi.org/10.1016/j.chemosphere.2018.05.012.

(14) Fabregat-Palau, J. Modelling the Sorption Behaviour of Perfluoroalkyl Carboxylates and Perfluoroalkane Sulfonates in Soils. *Sci. Total Environ.* **2021,** 801, 149343. https://doi.org/10.1016/j.scitotenv.2021.149343.

(15) Xiao, F.; Zhang, X.; Penn, L.; Gulliver, J. S.; Simcik, M. F. Effects of Monovalent Cations on the Competitive Adsorption of Perfluoroalkyl Acids by Kaolinite: Experimental Studies and Modeling. *Environ. Sci. Technol.* **2011**, *45* (23), 10028–10035. https://doi.org/10.1021/es202524y.

(16) Knight, E. R.; Janik, L. J.; Navarro, D. A.; Kookana, R. S.; McLaughlin, M. J. Predicting Partitioning of Radiolabelled 14C-PFOA in a Range of Soils Using Diffuse Reflectance Infrared Spectroscopy. *Sci. Total Environ.* **2019**, *686*, 505–513. https://doi.org/10.1016/j.scitotenv.2019.05.339.

(17) Milinovic, J.; Lacorte, S.; Vidal, M.; Rigol, A. Sorption Behaviour of Perfluoroalkyl Substances in Soils. *Sci. Total Environ.* **2015**, *511*, 63–71. https://doi.org/10.1016/j.scitotenv.2014.12.017.

(18) Li, F.; Fang, X.; Zhou, Z.; Liao, X.; Zou, J.; Yuan, B.; Sun, W. Adsorption of Perfluorinated Acids onto Soils: Kinetics, Isotherms, and Influences of Soil Properties. *Sci. Total Environ.* **2019**, *649*, 504–514. https://doi.org/10.1016/j.scitotenv.2018.08.209.

(19) Wei, C.; Song, X.; Wang, Q.; Liu, Y.; Lin, N. Influence of Coexisting Cr(VI) and Sulfate Anions and Cu(II) on the Sorption of F-53B to Soils. *Chemosphere* **2019**, *216*, 507–515. https://doi.org/10.1016/j.chemosphere.2018.10.098.

(20) Fabregat-Palau, J.; Rigol, A.; Grathwohl, P.; Vidal, M. Assessing Sorption of Fluoroquinolone Antibiotics in Soils from a Kd Compilation Based on Pure Organic and Mineral Components. *Ecotox. Environ. Safe.* **2024**, *280*, 116535. https://doi.org/10.1016/j.ecoenv.2024.116535.

(21) Fabregat-Palau, J.; Yu, Z.; Zeng, X.; Vidal, M.; Rigol, A. Deriving Parametric and Probabilistic K_d Values for Fluoroquinolones in Soils. *Sci. Total Environ.* **2023**, *861*, 160266. https://doi.org/10.1016/j.scitotenv.2022.160266.

(22) Fabregat-Palau, J.; Vidal, M.; Rigol, A. Examining Sorption of Perfluoroalkyl Substances (PFAS) in Biochars and Other Carbon-Rich Materials. *Chemosphere* **2022**, *302*, 134733. https://doi.org/10.1016/j.chemosphere.2022.134733.

(23) Saeidi, N.; Lai, A.; Harnisch, F.; Sigmund, G. A FAIR Comparison of Activated Carbon, Biochar, Cyclodextrins, Polymers, Resins, and Metal Organic Frameworks for the Adsorption of per- and Polyfluorinated Substances. *Chem. Eng. J.* **2024**, *498*, 155456. https://doi.org/10.1016/j.cej.2024.155456.

(24) Kleineidam, S.; Schüth, C.; Grathwohl, P. Solubility-Normalized Combined Adsorption-Partitioning Sorption Isotherms for Organic Pollutants. *Environ. Sci. Technol.* **2002**, *36* (21), 4689–4697. https://doi.org/10.1021/es010293b.

(25) Brusseau, M. L.; Anderson, R. H.; Guo, B. PFAS Concentrations in Soils: Background Levels versus Contaminated Sites. *Sci. Total Environ.* **2020**, *740*, 140017. https://doi.org/10.1016/j.scitotenv.2020.140017.

(26) Card, M. L.; Gomez-Alvarez, V.; Lee, W.-H.; Lynch, D. G.; Orentas, N. S.; Lee, M. T.; Wong, E. M.; Boethling, R. S. History of EPI Suite™ and Future Perspectives on Chemical Property Estimation in US Toxic Substances Control Act New Chemical Risk Assessments. *Environ. Sci.: Processes Impacts* **2017**, *19* (3), 203–212. https://doi.org/10.1039/C7EM00064B.

(27) Inoue, Y.; Hashizume, N.; Yakata, N.; Murakami, H.; Suzuki, Y.; Kikushima, E.; Otsuka, M. Unique Physicochemical Properties of Perfluorinated Compounds and Their Bioconcentration in Common Carp Cyprinus Carpio L. *Arch. Environ. Contam. Toxicol.* **2012**, *62* (4), 672–680. https://doi.org/10.1007/s00244-011-9730-7.

(28) Wang, Z.; MacLeod, M.; Cousins, I. T.; Scheringer, M.; Hungerbühler, K. Using COSMOtherm to Predict Physicochemical Properties of Poly- and Perfluorinated Alkyl Substances (PFASs). *Environ. Chem.* **2011**, *8* (4), 389. https://doi.org/10.1071/EN10143.

(29) Poggio, L.; De Sousa, L. M.; Batjes, N. H.; Heuvelink, G. B. M.; Kempen, B.; Ribeiro, E.; Rossiter, D. SoilGrids 2.0: Producing Soil Information for the Globe with Quantified Spatial Uncertainty. *Soil* **2021**, *7* (1), 217–240. https://doi.org/10.5194/soil-7-217-2021.

(30) Troyanskaya, O.; Cantor, M.; Sherlock, G.; Brown, P.; Hastie, T.; Tibshirani, R.; Botstein, D.; Altman, R. B. Missing Value Estimation Methods for DNA Microarrays. *Bioinformatics* **2001**, *17* (6), 520–525. https://doi.org/10.1093/bioinformatics/17.6.520.

(31) Shapiro, S. S.; Wilk, M. B. An Analysis of Variance Test for Normality (Complete Samples). *Biometrika* **1965**, 52, 591-611.

(32) D'Agostino, R.; Pearson, E. S. Tests for Departure from Normality. Empirical Results for the Distributions of b_2 and $\sqrt{b_1}$. *Biometrika* **1973**, 60, 613.

(33) Zimmerman, D. W. A Note on Preliminary Tests of Equality of Variances. *Brit. J. Math. Statis.* **2004**, *57* (1), 173–181. https://doi.org/10.1348/000711004849222.

(34) Tukey, J. W. Comparing Individual Means in the Analysis of Variance. *Biometrics* **1949**, *5* (2), 99. https://doi.org/10.2307/3001913.

(35) Welch, B. L. On the Comparison of Several Mean Values: An Alternative Approach. *Biometrika* **1951**, 38, 330-336.

(36) Games, P. A.; Howell, J. F. Pairwise multiple comparison procedures with unequal N's and/or variances: A Monte Carlo study. *J. Educ. Stat.* **1976**, 1, 113-125.

(37) McKight, P. E.; Najab, J. Kruskal-Wallis Test. *The Corsini Encyclopedia of Psychology*; Weiner, I. B., Craighead, W. E., Eds.; Wiley, 2010; pp 1–1. https://doi.org/10.1002/9780470479216.corpsy0491.

(38) Dinno, A. Nonparametric Pairwise Multiple Comparisons in Independent Groups Using Dunn's Test. *The Stata Journal: Promoting communications on statistics and Stata* **2015**, *15* (1), 292–300. https://doi.org/10.1177/1536867X1501500117.

(39) Higgins, C. P.; Luthy, R. G. Modeling Sorption of Anionic Surfactants onto Sediment Materials: An a Priori Approach for Perfluoroalkyl Surfactants and Linear Alkylbenzene Sulfonates. *Environ. Sci. Technol.* **2007**, *41* (9), 3254–3261. https://doi.org/10.1021/es062449j.

(40) Umeh, A. C.; Naidu, R.; Shilpi, S.; Boateng, E. B.; Rahman, A.; Cousins, I. T.; Chadalavada, S.; Lamb, D.; Bowman, M. Sorption of PFOS in 114 Well-Characterized Tropical and Temperate Soils: Application of Multivariate and Artificial Neural Network Analyses. *Environ. Sci. Technol.* **2021**, *55* (3), 1779–1789. https://doi.org/10.1021/acs.est.0c07202.

(41) Xie, J.; Liu, S.; Su, L.; Zhao, X.; Wang, Y.; Tan, F. Elucidating Per- and Polyfluoroalkyl Substances (PFASs) Soil-Water Partitioning Behavior through Explainable Machine Learning Models. *Sci. Total Environ.* **2024**, *954*, 176575. https://doi.org/10.1016/j.scitotenv.2024.176575.

(42) Arp, H. P. H.; Gredelj, A.; Glüge, J.; Scheringer, M.; Cousins, I. T. The Global Threat from the Irreversible Accumulation of Trifluoroacetic Acid (TFA). *Environ. Sci. Technol.* **2024**, acs.est.4c06189. https://doi.org/10.1021/acs.est.4c06189.

(43) Moghadasi, R.; Mumberg, T.; Wanner, P. Spatial Prediction of Concentrations of Per- and Polyfluoroalkyl Substances (PFAS) in European Soils. *Environ. Sci. Technol. Lett.* **2023**, *10* (11), 1125–1129. https://doi.org/10.1021/acs.estlett.3c00633.

(44) Schüßler, M.; Capitain, C.; Bugsel, B.; Zweigle, J.; Zwiener, C. Non-Target Screening Reveals 124 PFAS at an AFFF-Impacted Field Site in Germany Specified by Novel Systematic Terminology. *Anal. Bioanal. Chem.* **2024**. https://doi.org/10.1007/s00216-024-05611-3.
